# Metal-free photo-induced sulfidation of aryl iodide and other chalcogenation

**DOI:** 10.3389/fchem.2022.941016

**Published:** 2022-07-26

**Authors:** Shuai Mao, Yahao Zhao, Zixuan Luo, Ruizhe Wang, Bo Yuan, Jianping Hu, Linghao Hu, San-Qi Zhang, Xiaoxing Ye, Mingliang Wang, Zhengkai Chen

**Affiliations:** ^1^ Department of Medicinal Chemistry, School of Pharmacy, Xi’an Jiaotong University, Xi’an, SN, China; ^2^ Xi’an Changqing Chemical Group Co., Ltd, Xi’an, SN, China; ^3^ Qingyuan Edible Fungi Research Center, Lishui, ZJ, China; ^4^ Institute of Materia Medica, Chinese Academy of Sciences, Shanghai, China; ^5^ Zhongshan Institute for Drug Discovery, Shanghai Institute of Materia Medica, Chinese Academy of Sciences, Zhongshan, China; ^6^ Key Laboratory of Surface & Interface Science of Polymer Materials of Zhejiang Province, Department of Chemistry, Zhejiang Sci-Tech University, Hangzhou, China

**Keywords:** metal-free, photo-induced, aryl sulfides, cross-coupling, disulfides

## Abstract

A photo-induced C-S radical cross-coupling of aryl iodides and disulfides under transition-metal and external photosensitizer free conditions for the synthesis of aryl sulfides at room temperature has been presented, which features mild reaction conditions, broad substrate scope, high efficiency, and good functional group compatibility. The developed methodology could be readily applied to forge C-S bond in the field of pharmaceutical and material science.

## Introduction

**FIGURE 1:**
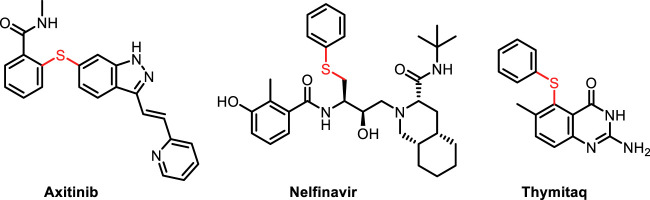
Representative drugs containing aryl sulfide motif.

**FIGURE 2:**
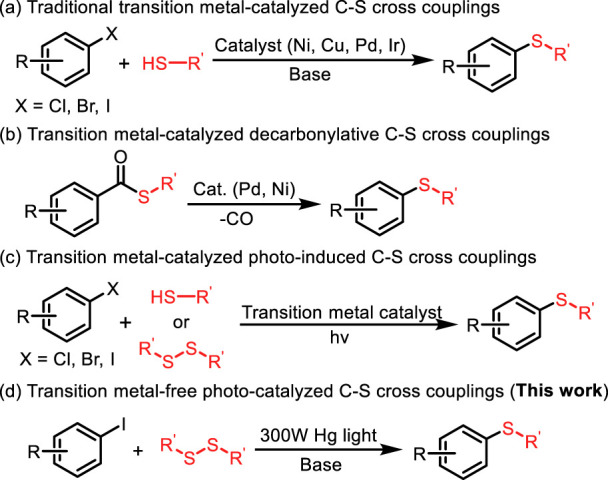
The C‐S formation methods with aryl halides and thiols/disulfides.

Aryl sulfides, as a ubiquitous structural motif in functional molecules, plays a unique role in pharmaceutical (Le Grand et al., 2008; Ilardi et al., 2014; Xiao et al., 2019; Golosov et al., 2021; Mourtas et al., 2020; Hai et al., 2021; Liu and Jiang, 2013; Barce Ferro et al., 2020) and material science (Figure 1). (Boyd 2016; Lu et al., 2017; Zhang et al., 2018; Hou et al., 2022) Hence, the development of efficient methods for the construction of C-S bond has attracted considerable attentions from chemical researchers. Traditionally, transition metal-catalyzed cross coupling of aryl halides and thiol/thiophenol constitutes the mainstream route to aryl sulfides (Figure 2A) (Fernández-Rodríguez et al., 2006; Alvaro and Hartwig, 2009; Timpa et al., 2014; Fu et al., 2015; Amiri et al., 2016a; Guzmán-Percástegui et al., 2016; Shiri et al., 2016; Chen et al., 2019; Gupta 2022; Jones et al., 2018; Liu et al., 2019; Isshiki et al., 2021; Cheng-Yi Wang et al., 2021; Yu et al., 2021). However, several troublesome drawbacks still exist, including expensive catalysts and ligands, high temperature and narrow substrate scope. In addition, the metal catalyst-poisoning enabled by thiols further limited the applicability of these reactions, so several alternatives to thiols had been explored (Clark et al., 1989; Akkilagunta and Kakulapati, 2011; Ke et al., 2011; Park et al., 2011; Prasad and Sekar, 2011; Reddy et al., 2011; Singh et al., 2013; Zhao et al., 2013; Firouzabadi et al., 2015; Tber et al., 2016; Ge et al., 2019; Liu et al., 2020). Although the use of thiol substitutes can eliminate the difficulties caused by thiols, these methods also have certain disadvantage, such as the tedious procedures for the synthesis of *S-*Alkylisothiouronium salt (Zhao et al., 2007), 1,3-propanedithiol equivalent (Liu et al., 2003) and 2-[bis (alkylthio) methylene]-3-oxo-*N*-*o*-tolylbutanamides (Dong et al., 2006). The pursuit of more efficient and environment-friendly approaches for the formation of C-S bond is of great urgency. Consequently, numerous newly developed methods emerge to replace the traditional methods. For instance, transition metal-catalyzed decarbonylation of thioester from readily available carboxylic acids has been regarded as another noteworthy strategy for achieving C–S bond formation (Figure 2B). (Ichiishi et al., 2018; Bie et al., 2021; Han Cao et al., 2021; Liu and Szostak, 2021) Reduction of sulfoxides also serves as a direct route to sulfides (Shiri and Kazemi, 2017; Lin et al., 2021; Zhang 2022). The synthesis of aryl sulfides using phenylboronic acid as substrate requires the participation of magnetic nanoparticles or expensive transition metals (Xu et al., 2012; Rostami et al., 2015a; Rostami et al., 2015b; Amiri et al., 2016a; Amiri et al., 2016b; Rostami et al., 2017; Wang et al., 2017; Cheng et al., 2018; Farzin et al., 2018; Atashkar et al., 2019; Gavhane et al., 2019; Khakyzadeh et al., 2019). With the development of photocatalytic reaction, photocatalytic synthesis of aryl sulfide has become an effective way. In the past few years, the photoredox transition metal-catalyzed C–S cross-coupling between aryl halides and thiols/disulfides have been widely developed, in which a series of Cu, Ni, Pd, and Rh transition metals are still utilized as catalysts (Figure 2C). (Uyeda et al., 2013; Wang et al., 2013; Johnson et al., 2016; Jouffroy et al., 2016; Jouffroy et al., 2016; Oderinde et al., 2016; Li et al., 2020; Sandfort et al., 2020; Brahmachari et al., 2021; Qin et al., 2021; Yang et al., 2021)

Meanwhile, A series of photo-induced transition-metal and photosensitizer free C−S cross-coupling methods has been developed (Bunnett and Creary, 1974; Liu et al., 2017; Pramanik et al., 2020; Dawei Cao et al., 2021; Nandy et al., 2021; Saroha et al., 2021; Shun Wang et al., 2021; Uchikura et al., 2021; Wang et al., 2022). For the metal-free synthesis of aryl sulfides, Hong and co-workers developed a convergent, organocatalytic visible-light-mediated process for the synthesis of diaryl sulfides (Hong et al., 2017). Kibriya’s group developed a metal-free visible-light-promoted oxidative coupling between thiols and arylhydrazines to afford diaryl sulfides using a catalytic amount of rose bengal as photocatalyst under aerobic conditions (Kibriya et al., 2018). Inspired by the aforementioned seminal studies and our pursuit of developing greener and more sustainable methods to forge C-S bonds, we have developed a metal-free photo-catalyzed C-S cross-coupling of aryl iodide and disulfides for the efficient synthesis of aryl sulfides under mild conditions (Figure 2D). It is worth noting that the present reaction features many advantages, including the use of clean and renewable light source, no participation of metal and photosensitizer, high efficiency, and excellent functional group compatibility, providing an environmentally friendly and expedient approach for the construction of aryl sulfides and congeners.

## Results and discussion

The C-S cross-coupling between 4-iodophenol (**1e**) and 1,2- diphenyldisulfide (**2a**) was selected as the model reaction for the optimization of reaction conditions. The reaction parameters, including the equivalent of disulfide, light, solvent, base, reaction time, were examined and the results were summarized in Table 1; Supplementary Table S1, S2. The investigation towards the amounts of disulfides was first conducted, as demonstrated that 0.5 equiv. of diphenyldisulfide **2a** enabled the formation of 4-(phenylthio) phenol (**3e**) in 56% yield (Table 1, Entry 1). The further increase of the disulfide amounts failed to improve the reaction yields (Table 1, Entry 2-3 and Supplementary Table S2). Then, the base effect of the reaction was investigated by using diverse organic and inorganic bases, which indicated that 50 mol% of TMG (1,1,3,3-Tetramethylguanidine) could give the best result (Table 1, entries 4-7 and Supplementary Table S2). Other additives were also tested and inferior yields were obtained (Supplementary Table S2). Subsequently, the screening of organic solvents revealed DMSO and ethyl acetate acted as the good medium, whereas other protic solvents and non-polar solvents led to the yield decline Table 1, entries 8-10 and Supplementary Table S2). Furthermore, shortening the reaction time had a detrimental impact on the reaction, whereas prolonging the reaction time to 18 h or 24 h could not obviously promote the reaction yield (Table 1, entries 12-14). The sources of light were also evaluated by the employment of 500 W Xe lamp, blue and green LED, 35 W white fluorescent lamp and in the dark, but no product **3e** was detected under the above conditions. (Table 1, entry 14 and Supplementary Table S1). Finally, the air atmosphere could sharply inhibit the reaction. (Table 1, entry 15 and Supplementary Table S2).

**TABLE 1:** Optimization of the reaction conditions^a^.

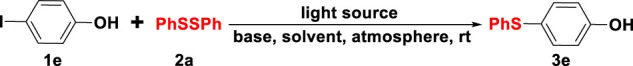							
**Entry**	**2a (equiv)**	**Light**	**Solvent**	**Base (equiv)**	**Time (h)**	**Yield [%]** ^b^	
1	0.5	300WHg	CH_3_CN	DBU (1)	12	56	
2	1	300WHg	CH_3_CN	DBU (1)	12	47	
3	2	300WHg	CH_3_CN	DBU (1)	12	43	
4	0.5	300WHg	CH_3_CN	DBU (0.5)	12	63	
5	0.5	300WHg	CH_3_CN	o-Anisidine (0.5)	12	79	
6	0.5	300WHg	CH_3_CN	TMG (0.5)	12	82	
7^c^	0.5	300WHg	CH_3_CN	Others	12	<80	
8	**0.5**	**300WHg**	**EA**	**TMG (0.5)**	12	**89 (87)**	
9	0.5	300WHg	DMSO	TMG (0.5)	12	81	
10^d^	0.5	300WHg	Others	TMG (0.5)	12	<80	
11	0.5	300WHg	EA	TMG (0.5)	6	37	
12	0.5	300WHg	EA	TMG (0.5)	18	88	
13	0.5	300WHg	EA	TMG (0.5)	24	85	
14^e^	0.5	Others	EA	TMG (0.5)	12	NR	
15^f^	0.5	300WHg	EA	TMG (0.5)	12	28	

a Reaction conditions: **1e** (0.1 mmol, c = 0.1 mol/L), **2a** (0.05 mmol), RT, N_2_, 12 h.

b Yield was determined by ^1^H NMR, with 1,3,5-trimethoxybenzene as an internal standard and the isolated yields were given in parenthesis.

c See the supporting Information.

d See the supporting Information.

e See the supporting Information.

f Reaction was carried out under air. TMEDA: *N, N, N, N*-tetramethylethylenediamine; DIPEA: *N, N*-Diisopropylethyl-amine; DBU: 1,8-Diazabicyclo [5.4.0] undec-7-ene; TMG: *N, N′, N′*-tetramethyl-guanidine; DABCO: 1,4-Diazabicyclo [2.2.2]octane; DMAP: *N*-(4-Pyridyl) dimethylamine; THF: tetrahydrofuran; DMF: *N, N*-Dimethylformamide; EA: ethyl acetate.

The bold values (Entry 8) is the best optimized reaction condition.

With the optimized reaction conditions in hand, the scope of the photo-catalyzed C-S cross-coupling was investigated with a variety of (hetero) aryl iodides and disulfides and the results were summarized in Table 2. Various substituted phenyl iodides reacted smoothly with diphenyldisulfide to deliver the corresponding aryl sulfides **3a–i** in good yields, irrespective of the electronic effect of the substituents. Noteworthy is that several sensitive functional groups, such as −OH, −NO_2_ and allyl, were all tolerant in the current reaction system (**3e–g**, **3ab**). The transformation is also applicable to the aryl iodides with strong electron-withdrawing groups, producing the sulfide products **3h–i** in 68%–80% yields. In addition, *p*-tolyl disulfide and 4,4′- dithiodiphenol were viable substrates to participate in the reaction to lead to the desired products **3aa** and **3ab** with high efficiency.

**TABLE 2:** Scope of the C-S coupling of (hetero)aryl iodides with diphenyldisulfide.

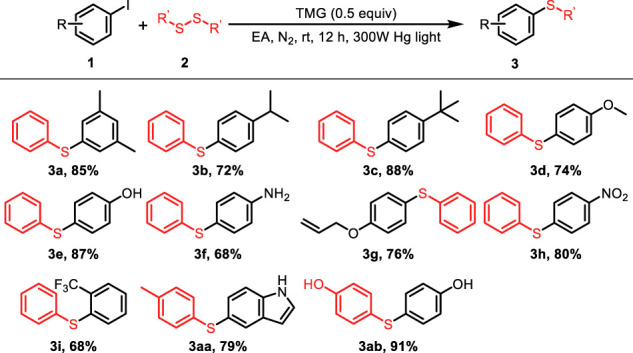

General conditions: 1 (0.1 mmol), 2 (0.05 mmol), TMG (0.05 mmol), EA (1.0 ml) at 25 C for 12 h under N_2_.

The substrate scope of the photo-catalyzed C-S cross-coupling was further extended with different (hetero) aryl iodides and dimethyl disulfide. As shown in Table 3, the cross coupling of (hetero) aryl iodides with dimethyl disulfide proceeded to afford the methyl (aryl) sulfane products **3j-z** in moderate to excellent yields. Various substituents, including −CN, −CO_2_Me, −CF_3_, naphthalene and heterocyclic scaffolds, were all compatible with the reaction system. The electron effect and the steric hindrance of the (hetero) aryl iodides exerted a marginal influence on the reaction, as verified by the comparable yields of the obtained products. The observation depicted in Tables 2, 3 exhibited the broad substrate scope and excellent functional group tolerance of the photo-catalyzed C-S bond cross coupling reaction.

**TABLE 3:** Scope of the C-S coupling of (hetero)aryl iodides with dimethyl disulfide.

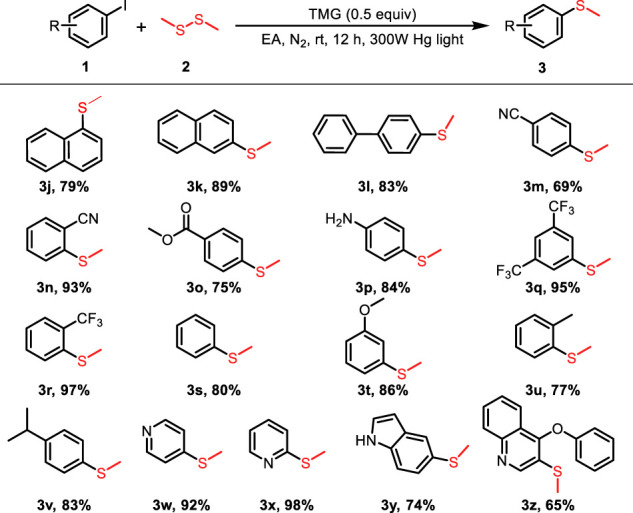

General conditions: 1 (0.1 mmol), 2 (0.05 mmol), TMG (0.05mmol), EA (1.0 ml) at 25 C for 12 h under N2.

The protocol could also be applied to construct C-Se and C-Te bond by using diselenide and ditelluride as coupling partners (Table 4). A series of aryl selenoethers 3ac-am were obtained in moderate to excellent yields, in which several sensitive functional groups and strong electron-withdrawing substituents were tolerant. In addition, two diaryltellane products **3an** and **3ao** were furnished with high efficiency under the current photo-reduced reaction system.

**TABLE 4:** Scope of the C-Se/C-Te coupling of (hetero)aryl iodides with diselenide/ditelluride.

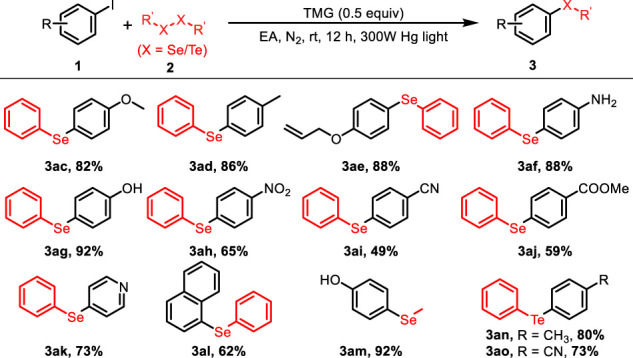

General conditions: 1 (0.1 mmol), 2 (0.05 mmol), TMG (0.05mmol), EA (1.0 ml) at 25°C for 12 h under N2.

**FIGURE 3:**
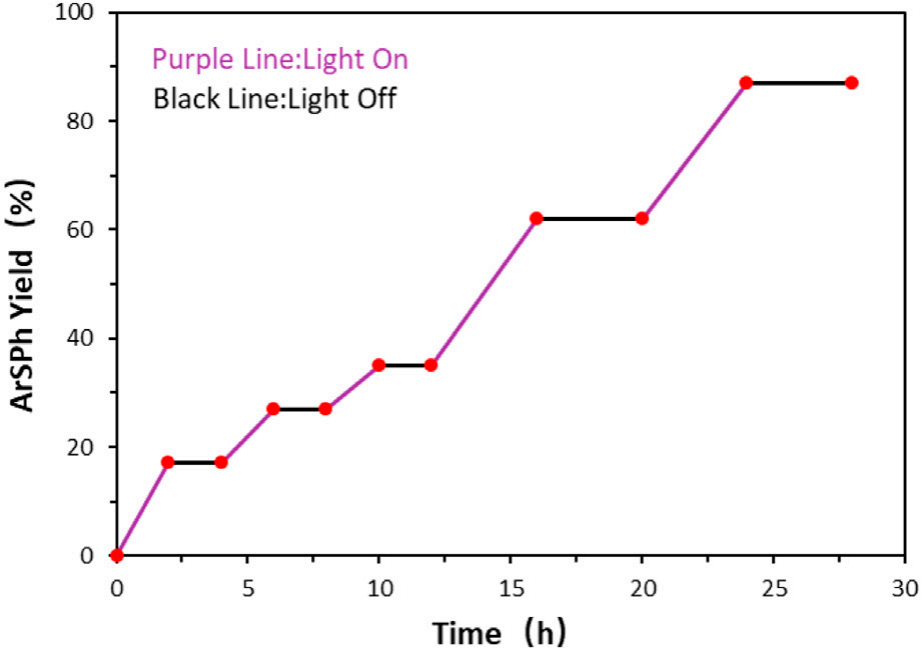
On-off experiment kinetic profile.

To investigate the mechanism of sulfuration reaction, the on-off experiment was carried out and no product formation was observed during the dark conditions in this experiment (Figure 3). It demonstrated that light irradiation is crucial for this sulfuration reaction and a radical chain propagation pathway is possibly not involved in the reaction.

**FIGURE 4:**
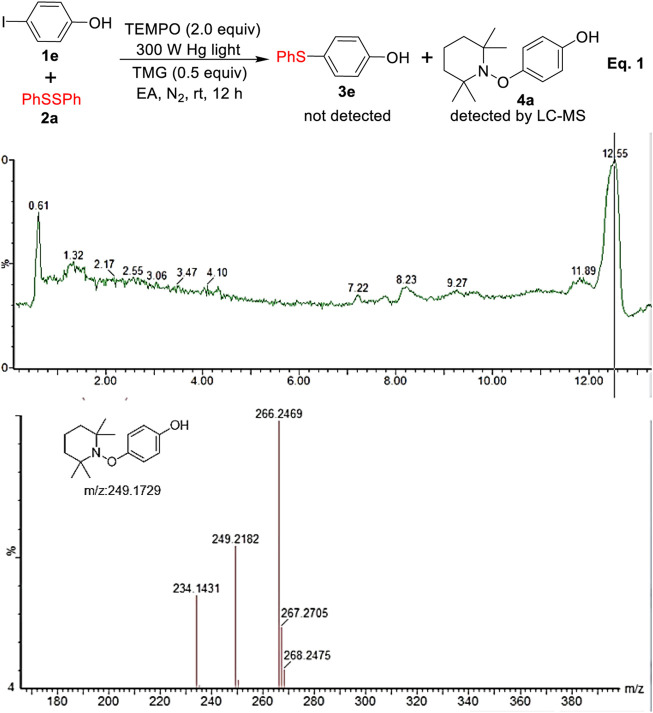
The radical trapping experiment.

To gain mechanistic understanding of this transformation, we turned our attention toward exploring the key intermediate and the nature of the reaction pathway. As depicted in Eq. 1 and Figure 4, when the radical scavengers of TEMPO (2,2,6,6-tetramethylpiperidine-*N*-oxide) was added into the reaction, the desired product **3e** was not detected at all and the coupling product **4** of aryl radical with TEMPO was successfully detected by LC-MS, which indicated that the reaction possibly involved a radical process.

**FIGURE 5:**
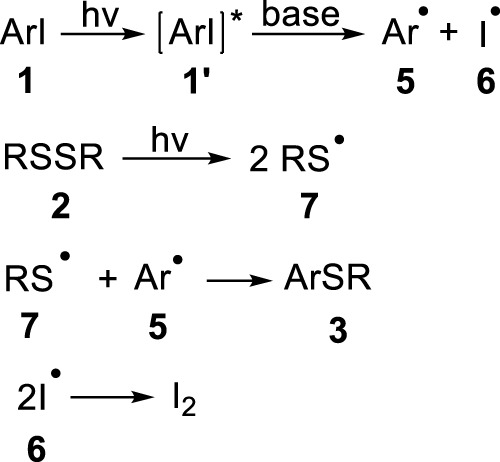
Proposed reaction mechanism.

On the basis of the above experimental results and related literatures (Discekici et al., 2015; Dong et al., 2019; Wu et al., 2019), a plausible mechanism of this reaction has been proposed (Figure 5). Under the irradiation of ultraviolet light, the aryl iodide **1** absorbs energy to reach the excited state **1**’, and the bond of disulfide subsequently undergoes homolysis process to produce sulfur radical **7** (Schmidt et al., 1964; Ogawa et al., 1998). Then, the strong base cleaves the carbon-iodine bond uniformly to produce aryl radical **5** and iodine radical **6**. Finally, the cross coupling of free radicals **7** and **5** delivers the desired aryl sulfoether product **3**. In addition, the remaining iodine radicals are transformed into elemental iodine through homocoupling reaction^1^.

## Conclusion

In conclusion, an efficient and transition metal-free photo-catalyzed C-S cross-coupling reaction have been developed. An array of (hetero) aryl sulfides could be accessed from the readily available aryl/hetero iodides and disulfides in an easily-operative and environment-friendly manner. A series of aryl selenoethers and diaryltellanes were also delivered by the developed method. The protocol is also charactered by no participation of metal catalyst or photosensitizer, broad substrate scope and good functional group tolerance. It is expected that this methodology will have wide application in the synthesis of functional sulfur-containing molecules in the field of pharmaceutical and material science.

## Data Availability

The original contributions presented in the study are included in the article/Supplementary Material, further inquiries can be directed to the corresponding authors.

## Footnotes

1 According to standard conditions, the system was dark-brown after the reaction was completed. When an appropriate amount of sodium thiosulfate solution was added, the system became colourless and transparent. The comparison before and after the reaction can be found in the SI.
