# Supplementary material for: Metal-free photo-induced sulfidation of aryl iodide and other chalcogenation
Source: Front Chem. 2022 Jul 26;10:941016. doi: 10.3389/fchem.2022.941016 (PMC9360480; doi:10.3389/fchem.2022.941016)

Contents

1. General Information.. S2

2. Setup of the Photochemical Reactions Instruments.. S3

3. Optimization of the Reaction Conditions... S3

4. General Procedures for the Photoinduced Sulfuration Reactions S6

5. Characterization Data for the Products S7

6. Mechanism of the Sulfuration Reaction..S22

7. References S24

8. NMR Spectra for the Products………….. S27

1. General Information

Experimental. Air- and moisture-sensitive reactions were carried out in sealed tube or glassware sealed with a rubber septum under an atmosphere of dry nitrogen. Air- and moisture-sensitive liquids and solutions were transferred by syringe. Reactions were stirred using Teflon-coated magnetic stir bars. All photochemical reactions carried out in the Bilon photoreactor (BL-GHX-V). Organic solutions were concentrated using a rotary evaporator with vacuum pump. The progress of the reactions was monitored by TLC with silica gel plates (GF_{254}), and the visualization was carried out under ultraviolet light (λ=254 nm). Purification of products was accomplished by flash column chromatography on silica gel (HAIYANG silica gel 3, 100-200 mesh).

Chemicals. Commercial reagents were purchased from Energy, Sigma Aldrich, Alfa Aesar, Acros Organics, Aladdin, Strem Chemicals, TCI, Bide Pharmatech Ltd. and used as received or purified according to Purification of Common Laboratory Chemicals. MeCN, MeOH and Acetone were purchased from Acros Organics and used directly without further purification. Distilled water was degassed with sonication under vacuum and then backfilled with nitrogen. Substrates 1-(allyloxy)-4-iodobenzene (1g)[1] was synthesized following the published procedures.

Analytical. NMR spectra were recorded on a Bruker AVANCE III 400 MHz and AVANCE III HD 600 MHz spectrometer and chemical shifts (δ) are reported in parts per million (ppm). The ^{1}H NMR were calibrated against the peak of tetramethylsilane (TMS, 0 ppm). The ^{13}C NMR were calibrated against the peak of the solvent (77.16 ppm for $CDCl_3$). It referenced to corresponding solvent resonance, coupling constants are reported in Hz with multiplicities denoted as s (singlet), d (doublet), t (triplet), q (quartet), m (multiplet) and br (broad). GC analysis was performed on a Shimadzu GC-MS-QP 2010 ultra-instrument equipped with a FID detector using nitrogen as the carrier gas.

2. Setup of the Photochemical Reactions Instruments

All photochemical reactions were performed in a 10 mL sealed tube made from boronsilicate glass, and the tube was placed onto magnetic stirrer fitted with a 300 W high-pressure mercury lamp plate, which is driven by a controllable current power supply (0-100 V, 0-5 A). The quartz immersion well, connected cooling liquid circulating pump, was utilized to keep the reaction at room temperature (25 °C). This reaction setup is detailed in Figure S1

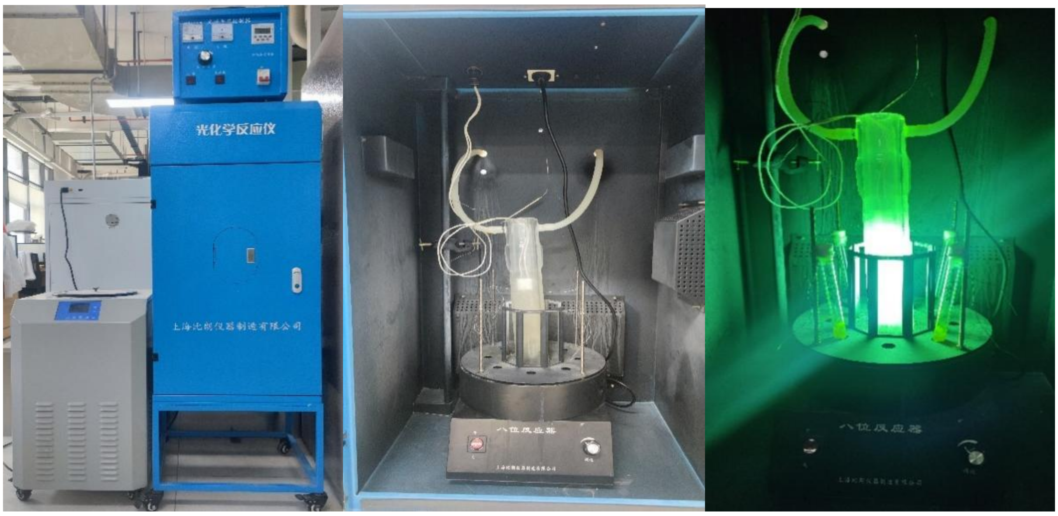

Figure S1. Photochemical batch reactor.

3. Reaction optimization

General procedure: A 10 mL reaction quartz tube was charged with a magnetic stir bar, **1e** (22.0 mg, 0.1 mmol), base (0.1 mmol), sulfur source (0.1 mmol) and 1 mL of solvent. The tube was sealed with a screw cap and put into the photoreactor for 12 h (the experiment setup is shown in Figure S1). After then, a standard solution of 1,3,5-trimethoxybenzene in toluene was added as the internal standard, followed by water. After shaking for 1 min, the organic layer was separated and analyzed by ^{1}H NMR to obtain yield of the reaction. The data was listed in the following table.

Table S1. Optimization of the reaction light sources.

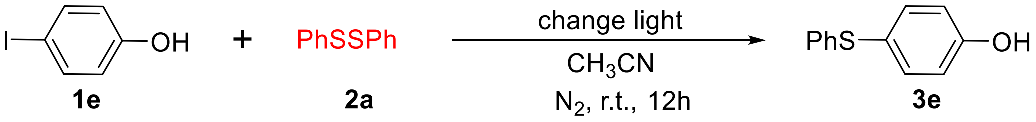

Entry	Light source	Solvent	Additive (mol %)	Time (h)	Yield [%]
1	300 W Hg lamp	CH_3CN	None	12	20
2	500 W Xe lamp	CH_3CN	None	12	NR
3	Green LED	CH_3CN	None	12	NR
4	Blue LED	CH_3CN	None	12	NR
5	35W CoolDaylight	CH_3CN	None	12	NR
6	Dark	CH_3CN	None	12	NR

Reaction conditions: **1e** (0.1 mmol, c = 0.1 mol/L), **2a** (0.1 mmol), RT, N_2, 12 h;

Table S2. Optimization of the reaction conditions.

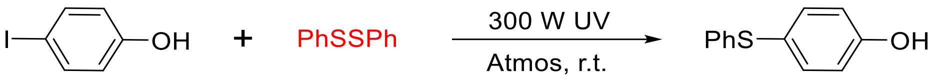

1e **2a** **3e**

Entry	2 (eq)	Solvent	Additive (eq)	Atmos	Time (h)	Yield [%] [b]
**1**	0.1	CH_3CN	DBU (1)	N_2	12	5
**2**	0.25	CH_3CN	DBU (1)	N_2	12	12
**3**	0.5	CH_3CN	DBU (1)	N_2	12	56
**4**	1	CH_3CN	DBU (1)	N_2	12	47
**5**	2	CH_3CN	DBU (1)	N_2	12	43
**6**	0.5	CH_3CN	DBU (0.5)	N_2	12	63
**7**	0.5	CH_3CN	DBU (2)	N_2	12	52
**8**	0.5	CH_3CN	KOH (0.5)	N_2	12	34
**9**	0.5	CH_3CN	$CsCO_3$ (0.5)	N_2	12	20
**10**	0.5	CH_3CN	*t*-BuOLi (0.5)	N_2	12	68
**11**	0.5	CH_3CN	CH_3ONa (0.5)	N_2	12	58
**12**	0.5	CH_3CN	$(C_2H_5)_3N$ (0.5)	N_2	12	57
**13**	0.5	CH_3CN	DIPEA (0.5)	N_2	12	25
**14**	0.5	CH_3CN	TMEDA (0.5)	N_2	12	19
**15**	0.5	CH_3CN	DABCO (0.5)	N_2	12	45
**16**	0.5	CH_3CN	Diisopropylamine (0.5)	N_2	12	26

**17**	0.5	CH_3CN	1,4-butanediamine (0.5)	N_2	12	31
**18**	0.5	CH_3CN	*N, N*-Dimethyl-1,2-ethanediamine (0.5)	N_2	12	27
**19**	0.5	CH_3CN	*o*-Anisidine (0.5)	N_2	12	79
**20**	0.5	CH_3CN	*p*-Toluenesulfonamide (0.5)	N_2	12	16
**21**	0.5	CH_3CN	TMG (0.5)	N_2	12	82
**22**	0.5	CH_3CN	Pyridine (0.5)	N_2	12	NR
**23**	0.5	CH_3CN	DMAP (0.5)	N_2	12	18
**24**	0.5	CH_3CN	PPh_3 (0.5)	N_2	12	16
**25**	0.5	H_2O	TMG (0.5)	N_2	12	NR
**26**	0.5	CH_3CN/H_2O (1:1)	TMG (0.5)	N_2	12	72
**27**	0.5	CH_3CN/H_2O (1:2)	TMG (0.5)	N_2	12	76
**28**	0.5	CH_3CN/H_2O (1:5)	TMG (0.5)	N_2	12	75
**29**	0.5	1,4-Dioxane	TMG (0.5)	N_2	12	30
**30**	0.5	EA	TMG (0.5)	N_2	12	87
**31**	0.5	CH_2Cl_2	TMG (0.5)	N_2	12	55
**32**	0.5	CH_3CH_2OH	TMG (0.5)	N_2	12	41
**33**	0.5	CH_3OH	TMG (0.5)	N_2	12	36
**34**	0.5	Toluene	TMG (0.5)	N_2	12	48
**35**	0.5	THF	TMG (0.5)	N_2	12	13
**36**	0.5	DMF	TMG (0.5)	N_2	12	5
**37**	0.5	DMSO	TMG (0.5)	N_2	12	81
**38**	0.5	EA	———	N_2	12	20
**39**[a]	**0.5**	**EA**	**TMG (0.5)**	$\mathbf{N_2}$	**12**	**89**
**40**	0.5	EA	TMG (0.5)	N_2	6	37

**41**	0.5	EA	TMG (0.5)	N_2	18	88
**42**	0.5	EA	TMG (0.5)	N_2	24	85
**43**	0.5	EA	TMG (0.5)	Air	12	28

[a] Reaction conditions: **1e** (0.1 mmol, c = 0.1 mol/L), **2a** (0.05mmol), RT, N_2, 12 h.

[b] Yield was determined by ^{1}H NMR with 1,3,5-trimethoxybenzene as an internal standard.

TMEDA: *N, N, N, N*-tetramethylethylenediamine;

DIPEA: *N, N*-Diisopropylethylamine;

DBU: 1,8-Diazabicyclo[5.4.0]undec-7-ene;

TMG: *N, N', N'*-tetramethyl-guanidine;

DABCO: 1,4-Diazabicyclo[2.2.2]octane;

DMAP: *N*-(4-Pyridyl)dimethylamine；

THF: Tetrahydrofuran；

DMF: *N, N*-Dimethylformamide;

EA: Ethyl acetate.

4. General Procedures for the Photo-induced Sulfuration Reactions

A solution of the aryl iodides (0.1 mmol), PhSSPh (**2a**)/MeSSMe (**2b**)/BnSSBn (**2c**)/4,4'-thiobis-phenol (**2d**)/PhSeSePh (**2e**)/MeSeSeMe (**2f**)/ PhTeTePh (**2g**) (0.05 mmol, 0.5 equiv), TMG (0.05 mmol/ 0.5 equiv) and the additive reagents in EA (1.0 mL) was added into a quartz test tube containing a magnetic stirring bar and the mixture was purged with nitrogen for 10 min. The tube was then capped with a septum. The reaction mixture was irradiated using a 300 W high-pressure mercury lamp through a water-colled quartz immersion well for 12 h. The reaction mixture was then spin-dried in vacuo, then dissolved in ethyl acetate, washed three times with deionized water, and the organic layer was separated. It was washed three times with saturated brine, and the organic layer was dried with an appropriate amount of anhydrous Na_2SO_4, filtered, and concentrated in vacuo. The crude product was purified by silica gel column chromatography (petroleum ether/ethyl acetate) to obtain target aryl sulfide.

5. Characterization Data for the Products

1-(allyloxy)-4-iodobenzene (1d)

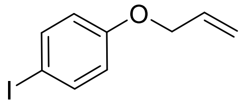

1**d** was obtained as colorless oil in 85% yield (22.1 mg) from 4-iodophenol (**1e**, 22.0 mg), allyl bromide (10.3 mg), potassium carbonate (13.8 mg) and acetonitrile (1ml) under reflux condition reaction 2h, then using 2:1 petroleum ether / Dichloromethane as eluent. ^{1}H NMR (400 MHz, Chloroform-d) δ 7.61 – 7.49 (m, 2H), 6.73 – 6.62 (m, 2H), 6.02 (ddt, J = 17.3, 10.5, 5.3 Hz, 1H), 5.47 – 5.22 (m, 2H), 4.49 (dt, J = 5.2, 1.6 Hz, 2H). ^{13}C NMR (101 MHz, Chloroform-d) δ 158.53, 138.29, 132.90, 118.07, 117.25, 83.01, 68.93. The NMR data were in consistent with the reported data[1].

(3,5-dimethylphenyl)(phenyl)sulfane (3a)

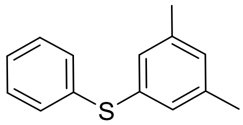

Following general procedure, **3a** was obtained as colorless oil in 85% yield (18.2 mg) from 1-iodo-3,5-dimethylbenzene (**1a**, 23.2 mg) and Diphenyl disulfide (**2a**,10.9 mg) using petroleum ether as eluent. ^{1}H NMR (400 MHz, Chloroform-d) δ 7.33 – 7.25 (m, 4H), 7.25 – 7.18 (m, 1H), 6.99 (d, J = 1.6 Hz, 2H), 6.89 (s, 1H), 2.26 (s, 6H). ^{13}C NMR (101 MHz, Chloroform-d) δ 138.97, 136.46, 134.80, 130.59, 129.22, 129.16, 126.75, 21.25. The NMR data were in consistent with the reported data[2].

(4-isopropylphenyl)(phenyl)sulfane (3b)

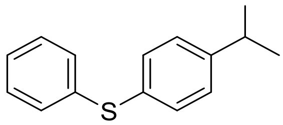

Following general procedure, **3b** was obtained as colorless oil in 72% yield (16.4 mg) from 1-iodo-4-isopropylbenzene (**1b**, 24.6 mg) and Diphenyl disulfide (**2a**,10.9 mg) using petroleum ether as eluent. ^{1}H NMR (400 MHz, Chloroform-d) δ 7.32 – 7.29 (m, 2H), 7.29 – 7.20 (m, 5H), 7.17 (d, J = 8.3 Hz, 2H), 2.88 (p, J = 6.9 Hz, 1H), 1.23 (d, J = 6.9 Hz, 6H). ^{13}C NMR (101 MHz, Chloroform-d) δ 148.51, 136.94, 132.08, 131.81, 130.15, 129.14, 127.59, 127.52, 126.60, 33.87, 23.98. The NMR data

were in consistent with the reported data[3].

(4-(tert-butyl)phenyl)(phenyl)sulfane (3c)

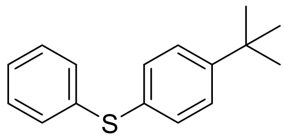

Following general procedure, **3c** was obtained as colorless oil in 88% yield (21.3 mg) from (4-iodophenyl)(phenyl)sulfane (**1c**, 26.0 mg) and Diphenyl disulfide (**2a**, 10.9 mg) using petroleum ether as eluent. ^{1}H NMR (400 MHz, Chloroform-d) δ 7.36 – 7.18 (m, 9H), 1.31 (s, 9H). ^{13}C NMR (101 MHz, Chloroform-d) δ 150.70, 136.74, 131.58, 130.36, 129.18, 127.59, 126.70, 126.42, 34.68, 31.37. The NMR data were in consistent with the reported data[4].

(4-methoxyphenyl)(phenyl)sulfane (3d)

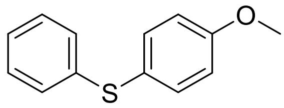

Following general procedure, **3d** was obtained as colorless oil in 74% yield (16.0 mg) from 1-iodo-4-methoxybenzene (**1d**, 23.4 mg) and Diphenyl disulfide (**2a**, 10.9 mg) using 100:1 petroleum ether/EtOAc as eluent. ^{1}H NMR (400 MHz, Chloroform-d) δ 7.42 (d, J = 8.7 Hz, 2H), 7.22 (d, J = 7.3 Hz, 2H), 7.19 – 7.10 (m, 3H), 6.93 – 6.86 (m, 2H), 3.82 (s, 3H). ^{13}C NMR (101 MHz, Chloroform-d) δ 159.93, 138.72, 135.51, 129.04, 128.26, 125.85, 124.34, 115.09, 55.47. The NMR data were in consistent with the reported data[5].

4-(Phenylthio)phenol (3e)

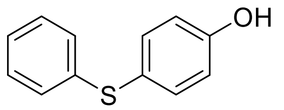

Following general procedure, **3e** was obtained as colorless oil in 92% yield (18.6 mg) from 4-iodophenol (**1e**, 22.0 mg) and Diphenyl disulfide (**2a**,10.9mg) using 10:1 petroleum ether/EtOAc as eluent. ^{1}H NMR (400 MHz, Chloroform-d) δ 7.37 (d, J=8.6 Hz, 2H), 7.23 (d, J=7.5 Hz, 2H), 7.20–7.10 (m, 3H), 6.83 (d, J=8.6 Hz, 2H), 4.99 (s, 1H). ^{13}C NMR (101 MHz, Chloroform-d) δ = 155.8, 138.4, 135.5, 128.9, 128.3, 125.8, 124.7, 116.5. The NMR data were in consistent with the reported

data[2].

4-(phenylthio)aniline (3f)

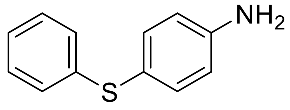

Following general procedure, **3f** was obtained as brown solid in 68% yield (13.7 mg) from 4-iodoaniline (**1f**, 21.9 mg) and Diphenyl disulfide (**2a**,10.9mg) using 10:1 petroleum ether/EtOAc as eluent. ^{1}H NMR (400 MHz, Chloroform-d) δ 7.34 – 7.30 (m, 2H), 7.22 (dd, J = 8.2, 7.0 Hz, 2H), 7.18 – 7.02 (m, 3H), 6.71 – 6.66 (m, 2H), 3.72 (s, 2H). ^{13}C NMR (101 MHz, Chloroform-d) δ 147.00, 139.75, 136.20, 128.92, 127.38, 125.36, 120.65, 116.04. The NMR data were in consistent with the reported data[6].

(4-(allyloxy)phenyl)(phenyl)sulfane (3g)

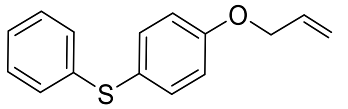

Following general procedure, **3g** was obtained as colorless oil in 76% yield (18.4 mg) from 1-(allyloxy)-4-iodobenzene (**1g**, 26.0 mg) and Diphenyl disulfide (**2a**,10.9mg) using petroleum ether as eluent. ^{1}H NMR (400 MHz, Chloroform-d) δ 7.43 – 7.36 (m, 2H), 7.25 – 7.19 (m, 2H), 7.19 – 7.10 (m, 3H), 6.94 – 6.87 (m, 2H), 6.05 (ddt, J = 17.3, 10.6, 5.3 Hz, 1H), 5.42 (dq, J = 17.3, 1.6 Hz, 1H), 5.30 (dq, J = 10.4, 1.4 Hz, 1H), 4.54 (dt, J = 5.3, 1.5 Hz, 2H). ^{13}C NMR (101 MHz, Chloroform-d) δ 158.91, 138.60, 135.38, 133.00, 129.04, 128.36, 125.90, 124.60, 118.09, 115.83, 68.99.

(4-Nitrophenyl)(phenyl)sulfane (3h)

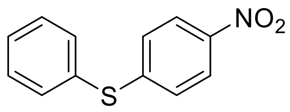

Following general procedure, **3h** was obtained as yellow oil in 50% yield (18.4 mg) from 1-iodo-4-nitrobenzene (**1h**, 24.9 mg) and Diphenyl disulfide (**2a**,10.9mg) using 100:1 petroleum ether/EtOAc as eluent. ^{1}H NMR (400 MHz, Chloroform-d) δ 8.13–7.97 (m, 2H), 7.70–7.50 (m, 2H), 7.50–7.42 (m, 3H), 7.21–7.10 (m, 2H). ^{13}C NMR (101 MHz, Chloroform-d) δ 148.5, 145.3, 134.8, 130.5, 130.1, 129.7, 126.7, 124.1. The NMR data were in consistent with the reported data[4].

phenyl(2-(trifluoromethyl)phenyl)sulfane (3i)

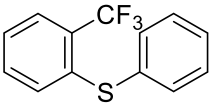

Following general procedure, **3i** was obtained as colorless oil in 68% yield (17.3 mg) from 1-iodo-2-(trifluoromethyl)benzene (**1i**, 27.2 mg) and Diphenyl disulfide (**2a**,10.9mg) using 100:1 petroleum ether/EtOAc as eluent. ^{1}H NMR (400 MHz, Chloroform-d) δ 7.67 (dd, *J* = 7.8, 1.6 Hz, 1H), 7.43 – 7.38 (m, 2H), 7.37 – 7.32 (m, 4H), 7.29 (dd, *J* = 2.6, 1.0 Hz, 1H), 7.18 (d, *J* = 7.9 Hz, 1H). ^{13}C NMR (101 MHz, Chloroform-d) δ 133.14, 132.42, 132.17, 129.62, 128.29, 126.34.The NMR data were in consistent with the reported data[5].

5-(p-tolylthio)-1H-indole (3aa)

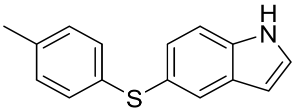

Following general procedure, **3aa** was obtained as brown solid in 79% yield (18.9 mg) from 5-iodo-1H-indole (**1aa**, 24.3 mg) and *p*-Tolyl Disulfide (**2c**, 12.3mg) using petroleum ether as eluent. ^{1}H NMR (400 MHz, Chloroform-d) δ 8.22 (s, 1H), 7.80 (dd, J = 1.7, 0.9 Hz, 1H), 7.35 (dt, J = 8.4, 0.8 Hz, 1H), 7.28 (dd, J = 8.5, 1.7 Hz, 1H), 7.22 (dd, J = 3.3, 2.5 Hz, 1H), 7.15 – 7.09 (m, 2H), 7.06 – 7.00 (m, 2H), 6.52 (ddd, J = 3.1, 2.1, 1.0 Hz, 1H), 2.28 (s, 3H). ^{13}C NMR (101 MHz, Chloroform-d) δ 135.66, 135.53, 129.74, 128.80, 127.68, 126.55, 125.14, 124.32, 112.09, 102.84, 21.06. The NMR data were in consistent with the reported data[7].

4,4'-thiodiphenol (3ab)

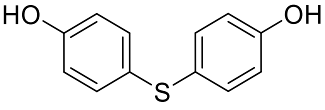

Following general procedure, **3ab** was obtained as white solid in 91% yield (19.9 mg) from 4-iodophenol (**1ab**, 22.0 mg) and 4,4'-thiodiphenol (**2d**, 12.5mg) using 10:1 petroleum ether/EtOAc as eluent. ^{1}H NMR (400 MHz, DMSO-d6) δ 9.60 (s, 2H), 7.12 – 7.07 (m, 4H), 6.72 – 6.68 (m, 4H). ^{13}C NMR (101 MHz, DMSO-d6) δ 157.53, 133.28, 125.18, 116.83. The NMR data were in consistent with the reported data[8].

methyl(naphthalen-1-yl)sulfane (3j)

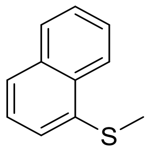

Following general procedure, **3j** was obtained as colorless oil in 79% yield (13.8 mg) from 1-iodonaphthalene (**1j**, 25.4mg) and Dimethyl Disulfide (**2b**, 4.7mg) using 100:1 petroleum ether/EtOAc as eluent. ^{1}H NMR (400 MHz, Chloroform-d) δ 8.32 (dd, J = 8.4, 1.5 Hz, 1H), 7.86 (dd, J = 7.7, 1.8 Hz, 1H), 7.70 (dt, J = 7.8, 1.2 Hz, 1H), 7.60 – 7.51 (m, 2H), 7.47 – 7.38 (m, 2H), 2.59 (s, 3H). ^{13}C NMR (101 MHz, Chloroform-d) δ 135.95, 133.74, 131.75, 128.67, 126.37, 126.27, 125.94, 125.82, 124.40, 123.67, 16.29. The NMR data were in consistent with the reported data[9].

methyl(naphthalen-2-yl)sulfane (3k)

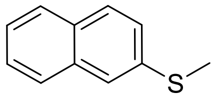

Following general procedure, **3k** was obtained as white solid in 89% yield (15.5 mg) from 2-iodonaphthalene (**1k**, 25.4mg) and Dimethyl Disulfide (**2b**, 4.7mg) using 100:1 petroleum ether/EtOAc as eluent. ^{1}H NMR (400 MHz, Chloroform-d) δ 7.76 (dd, J = 16.5, 7.7 Hz, 3H), 7.60 (d, J = 2.0 Hz, 1H), 7.50 – 7.35 (m, 3H), 2.59 (s, 3H). ^{13}C NMR (101 MHz, Chloroform-d) δ 136.17, 133.97, 131.34, 128.30, 127.38, 126.91, 126.67, 125.72, 124.85, 123.31, 15.85. The NMR data were in consistent with the reported data[10].

[1,1'-biphenyl]-4-yl(methyl)sulfane (3l)

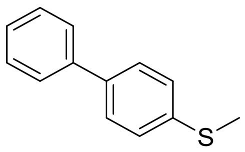

Following general procedure, **3l** was obtained as white solid in 83% yield (16.6 mg) from 4-iodo-1,1'-biphenyl (**1l**, 28.0 mg) and Dimethyl Disulfide (**2b**, 4.7mg) using 100:1 petroleum ether/EtOAc as eluent. ^{1}H NMR (400 MHz, Chloroform-d) δ 7.61 – 7.50 (m, 4H), 7.44 (t, J = 7.6 Hz, 2H), 7.39 – 7.29 (m, 3H), 2.52 (s, 3H).^{13}C NMR (101 MHz, Chloroform-d) δ 140.64, 138.13, 137.69, 128.93, 127.60, 127.32, 127.03, 126.95, 15.99. The NMR data were in consistent with the reported data[11].

4-(methylthio)benzonitrile (3m)

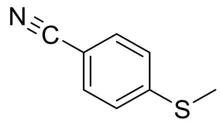

Following general procedure, **3m** was obtained as pale yellow solid in 69% yield (10.3 mg) from 4-Iodobenzonitrile (**1m**, 22.9 mg) and Dimethyl Disulfide (**2b**, 4.7mg) using 50:1 petroleum ether/EtOAc as eluent. ^{1}H NMR (400 MHz, Chloroform-d) δ 7.58 – 7.49 (m, 2H), 7.27 (s, 1H), 7.25 (d, J = 1.9 Hz, 1H), 2.51 (s, 3H). ^{13}C NMR (101 MHz, Chloroform-d) δ 146.21, 132.27, 125.58, 119.08, 107.76, 14.77. The NMR data were in consistent with the reported data[12].

2-(methylthio)benzonitrile (3n)

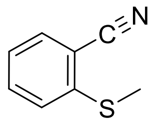

Following general procedure, **3n** was obtained as yellow solid in 93% yield (13.9 mg) from 2-Iodobenzonitrile (**1n**, 22.9 mg) and Dimethyl Disulfide (**2b**, 4.7mg) using 50:1 petroleum ether/EtOAc as eluent. ^{1}H NMR (400 MHz, Chloroform-d) δ 7.55 – 7.45 (m, 2H), 7.26 (dd, J = 8.4, 1.1 Hz, 1H), 7.16 (td, J = 7.6, 1.1 Hz, 1H), 2.51 (s, 3H). ^{13}C NMR (101 MHz, Chloroform-d) δ 143.70, 133.52, 133.15, 126.08, 125.16, 117.10, 111.34, 15.73. The NMR data were in consistent with the reported data[13].

methyl 4-(methylthio)benzoate (3o)

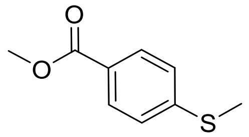

Following general procedure, **3o** was obtained as white solid in 75% yield (13.6 mg) from 4-iodobenzoate (**1o**, 26.2 mg) and Dimethyl Disulfide (**2b**, 4.7mg) using 40:1 petroleum ether/EtOAc as eluent. ^{1}H NMR (400 MHz, Chloroform-d) δ 8.31–7.63 (m, 2H), 7.25 (dd, *J*=9.0, 2.2, 2H), 3.90 (s, 3H), 2.51 (s, 3H). ^{13}C NMR (101 MHz, Chloroform-d) δ 166.9, 145.4, 129.9, 126.3, 124.9, 52.0, 14.8. The NMR data were in consistent with the reported data[12].

4-(methylthio)aniline (3p)

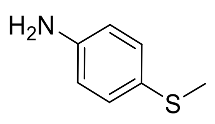

Following general procedure, **3p** was obtained as yellow oil in 84% yield (11.7 mg) from 4-iodoaniline (**1p**, 21.9 mg) and Dimethyl Disulfide (**2b**, 4.7mg) using 8:1 petroleum ether/EtOAc as eluent. ^{1}H NMR (400 MHz, Chloroform-d) δ 7.18 (d, *J*=8.5 Hz, 2H), 6.64 (d, *J*=8.5 Hz, 2H), 2.41 (s, 3H). ^{13}C NMR (101 MHz, Chloroform-d) δ 145.1, 131.1, 125.9, 115.8, 18.8. The NMR data were in consistent with the reported data[14].

(3,5-bis(trifluoromethyl)phenyl)(methyl)sulfane (3q)

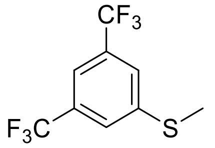

Following general procedure, **3q** was obtained as colorless oil in 95% yield (24.7 mg) from 1-iodo-3,5-bis(trifluoromethyl)benzene (**1q**, 34.0 mg) and Dimethyl Disulfide (**2b**, 4.7mg) using petroleum ether as eluent. ^{1}H NMR (400 MHz, Chloroform-d) δ 7.60 (d, J = 4.1 Hz, 3H), 2.56 (s, 3H). ^{13}C NMR (101 MHz, Chloroform-d) δ 142.46, 132.60, 132.26, 131.94, 131.60, 125.58, 124.58, 121.86, 118.50, 118.46, 118.42, 15.32. The NMR data were in consistent with the reported data[15].

methyl(2-(trifluoromethyl)phenyl)sulfane (3r)

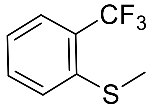

Following general procedure, **3r** was obtained as colorless oil in 97% yield (18.6 mg) from 1-iodo-2-(trifluoromethyl)benzene (**1r**, 27.2 mg) and Dimethyl Disulfide (**2b**, 4.7mg) using 50:1 petroleum ether/EtOAc as eluent. ^{1}H NMR (400 MHz, Chloroform-d) δ 7.61 (dd, J = 7.8, 1.4 Hz, 1H), 7.47 (td, J = 7.8, 1.7 Hz, 1H), 7.36 (d, J = 8.0 Hz, 1H), 7.25 – 7.18 (m, 1H), 2.51 (s, 3H). ^{13}C NMR (101 MHz, Chloroform-d) δ 138.32, 132.10, 128.58, 128.28, 127.98, 127.41, 126.84, 126.78, 126.72, 126.67, 125.38, 124.75, 122.65, 16.37. The NMR data were in consistent with the reported data[16].

methyl(phenyl)sulfane (3s)

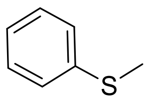

Following general procedure, **3s** was obtained as colorless oil in 80% yield (9.9 mg) from

iodobenzene (**1s**, 20.4 mg) and Dimethyl Disulfide (**2b**, 4.7mg) using 50:1 petroleum ether/EtOAc as eluent. ^{1}H NMR (400 MHz, Chloroform-d) δ 7.31 – 7.24 (m, 4H), 7.13 (tt, J = 7.1, 2.1 Hz, 1H), 2.48 (s, 3H). ^{13}C NMR (101 MHz, Chloroform-d) δ 138.49, 128.92, 126.68, 125.11, 77.47, 77.16, 76.84, 15.91. The NMR data were in consistent with the reported data[17].

methyl(phenyl)sulfane (3t)

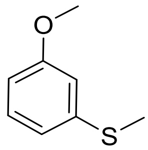

Following general procedure, **3t** was obtained as yellow oil in 86% yield (13.3 mg) from 1-iodo-3-methoxybenzene (**1t**, 23.4 mg) and Dimethyl Disulfide (**2b**, 4.7mg) using 50:1 petroleum ether/EtOAc as eluent. ^{1}H NMR (400 MHz, Chloroform-d) δ 7.19 (t, J = 8.0 Hz, 1H), 6.86 – 6.75 (m, 2H), 6.67 (ddd, J = 8.2, 2.5, 0.8 Hz, 1H), 3.79 (s, 3H), 2.47 (s, 3H). ^{13}C NMR (101 MHz, Chloroform-d) δ 159.96, 139.92, 129.75, 118.80, 112.14, 110.65, 55.33, 15.77. The NMR data were in consistent with the reported data[18].

methyl(o-tolyl)sulfane (3u)

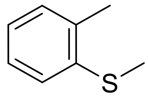

Following general procedure, **3u** was obtained as yellow oil in 77% yield (10.6 mg) from 1-iodo-2-methylbenzene (**1u**, 21.8 mg) and Dimethyl Disulfide (**2b**, 4.7mg) using 50:1 petroleum ether/EtOAc as eluent. ^{1}H NMR (400 MHz, Chloroform-d) δ 7.22 – 7.12 (m, 3H), 7.06 (td, J = 7.2, 1.7 Hz, 1H), 2.46 (s, 3H), 2.33 (s, 3H). ^{13}C NMR (101 MHz, Chloroform-d) δ 137.72, 135.80, 129.86, 126.56, 124.66, 124.57, 20.07, 15.30. The NMR data were in consistent with the reported data[19].

(4-isopropylphenyl)(methyl)sulfane (3v)

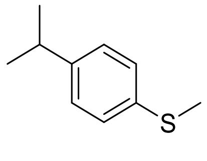

Following general procedure, **3v** was obtained as colorless oil in 83% yield (13.8 mg) from 1-

iodo-4-isopropylbenzene (**1v**, 24.6 mg) and Dimethyl Disulfide (**2b**, 4.7mg) using 50:1 petroleum ether/EtOAc as eluent. ^{1}H NMR (400 MHz, Chloroform-d) δ 7.23 – 7.18 (m, 2H), 7.15 (d, J = 8.3 Hz, 2H), 2.87 (p, J = 6.9 Hz, 1H), 2.46 (s, 3H), 1.23 (d, J = 6.9 Hz, 6H). ^{13}C NMR (101 MHz, Chloroform-d) δ 146.21, 135.15, 127.29, 127.11, 33.73, 24.07, 16.48. The NMR data were in consistent with the reported data[21].

4-(methylthio)pyridine (3w)

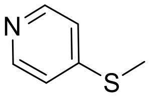

Following general procedure, **3w** was obtained as yellow oil in 92% yield (11.5 mg) from 4-iodopyridine (**1w**, 20.5 mg) and Dimethyl Disulfide (**2b**, 4.7mg) using 5:1 petroleum ether/EtOAc as eluent. ^{1}H NMR (400 MHz, Chloroform-d) δ 8.41 – 8.31 (m, 2H), 7.19 – 6.90 (m, 2H), 2.45 (s, 3H). ^{13}C NMR (101 MHz, Chloroform-d) δ 150.35, 149.18, 119.90, 13.72. The NMR data were in consistent with the reported data[20].

2-(methylthio)pyridine (3x)

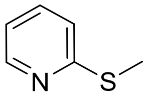

Following general procedure, **3x** was obtained as yellow oil in 98% yield (12.2 mg) from 2-iodopyridine (**1x**, 20.5 mg) and Dimethyl Disulfide (**2b**, 4.7mg) using 50:1 petroleum ether/EtOAc as eluent. ^{1}H NMR (400 MHz, Chloroform-d) δ 8.39 (dt, J = 5.0, 1.4 Hz, 1H), 7.43 (td, J = 7.7, 1.9 Hz, 1H), 7.19 – 7.08 (m, 1H), 6.93 (ddd, J = 7.4, 5.0, 1.1 Hz, 1H), 2.52 (s, 3H). ^{13}C NMR (101 MHz, Chloroform-d) δ 160.04, 149.50, 135.88, 121.52, 119.17, 13.31. The NMR data were in consistent with the reported data[12].

5-(methylthio)-1H-indole (3y)

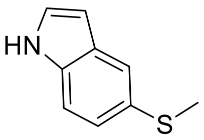

Following general procedure, **3y** was obtained as colorless oil in 74% yield (12.1mg) from 5-iodo-1H-indole (**1y**, 24.3 mg) and Dimethyl Disulfide (**2b**, 4.7mg) using 100:1 petroleum

ether/EtOAc as eluent. ^{1}H NMR (400 MHz, Chloroform-d) δ 8.12 (d, J = 21.9 Hz, 1H), 7.64 (d, J = 1.9 Hz, 1H), 7.32 (d, J = 8.5 Hz, 1H), 7.24 – 7.18 (m, 2H), 6.53 – 6.45 (m, 1H), 2.51 (s, 3H). ^{13}C NMR (101 MHz, Chloroform-d) δ 134.58, 128.74, 128.04, 124.98, 124.10, 121.42, 111.64, 102.37, 18.84.

3-(methylthio)-4-phenoxyquinoline (3z)

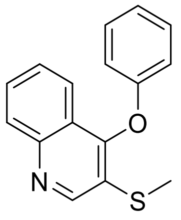

Following general procedure, **3z** was obtained as colorless oil in 65% yield (17.4 mg) from 3-iodo-4-phenoxyquinoline (**1z**, 34.7mg) and Dimethyl Disulfide (**2b**, 4.7mg) using 8:1 petroleum ether/EtOAc as eluent. ^{1}H NMR (400 MHz, Chloroform-d) δ 8.91 (s, 1H), 8.11 (d, J = 8.5 Hz, 1H), 7.87 (dd, J = 8.5, 1.4 Hz, 1H), 7.67 (ddd, J = 8.4, 6.8, 1.4 Hz, 1H), 7.47 (ddd, J = 8.2, 6.8, 1.1 Hz, 1H), 7.28 (dd, J = 8.7, 7.3 Hz, 2H), 7.05 (t, J = 7.3 Hz, 1H), 6.89 – 6.77 (m, 2H), 2.52 (s, 3H). ^{13}C NMR (101 MHz, Chloroform-d) δ 157.47, 155.25, 151.12, 148.56, 129.93, 129.56, 128.66, 128.05, 127.46, 125.12, 124.07, 123.51, 122.82, 121.87, 115.50, 16.14.

(4-methoxyphenyl)(phenyl)selane (3ac)

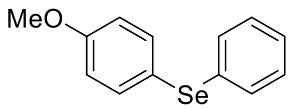

Following general procedure, **3ac** was obtained as colorless oil in 82% yield (21.6 mg) from 1-iodo-4-methoxybenzene (**1ac**, 23.4 mg) and Diphenyl diselenide（**2e**, 15.6mg）using petroleum ether as eluent. ^{1}H NMR (400 MHz, Chloroform-d) δ 7.54 – 7.49 (m, 2H), 7.37 – 7.31 (m, 2H), 7.24 – 7.15 (m, 3H), 6.91 – 6.81 (m, 2H), 3.81 (s, 3H). 13C NMR (101 MHz, Chloroform-d) δ 159.80, 136.54, 133.20, 130.92, 129.15, 126.46, 119.96, 115.14, 55.31. The NMR data were in consistent with the reported data.[22]

phenyl(p-tolyl)selane (3ad)

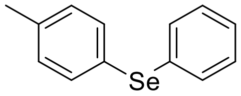

Following general procedure, **3ad** was obtained as colorless oil in 86% yield (21.3 mg) from 1-iodo-4-methylbenzene (**1ad**, 21.8 mg) and Diphenyl diselenide（**2e**, 15.6mg）using petroleum ether as eluent. 1H NMR (600 MHz, Chloroform-d) δ 7.40 (td, J = 4.4, 4.0, 1.9 Hz, 4H), 7.24 – 7.22 (m, 3H), 7.10 (d, J = 7.8 Hz, 2H), 2.33 (s, 3H). 13C NMR (151 MHz, Chloroform-d) δ 137.70, 133.93, 132.13, 132.09, 130.23, 129.25, 126.90, 126.82, 21.18. The NMR data were in consistent with the reported data.[23]

(4-(allyloxy)phenyl)(phenyl)selane (3ae)

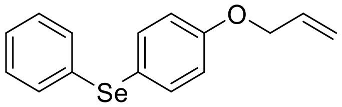

Following general procedure, **3ae** was obtained as white solid in 81% yield (23.5 mg) from 1-(allyloxy)-4-iodobenzene (**1ae**, 26.0 mg) and Diphenyl diselenide（**2e**, 15.6mg）using 50:1 petroleum ether/EtOAc as eluent. ^{1}H NMR (400 MHz, Chloroform-d) δ 7.51 – 7.47 (m, 2H), 7.36 – 7.31 (m, 2H), 7.24 – 7.16 (m, 3H), 6.90 – 6.84 (m, 2H), 6.05 (ddt, J = 17.4, 10.6, 5.3 Hz, 1H), 5.41 (dd, J = 17.3, 1.5 Hz, 1H), 5.30 (dd, J = 10.5, 1.3 Hz, 1H), 4.56 – 4.51 (m, 2H).13C NMR (101 MHz, Chloroform-d) δ 158.79, 136.44, 133.11, 132.97, 131.00, 129.16, 126.50, 120.20, 117.91, 115.91, 68.86.

4-(phenylselanyl)aniline (3af)

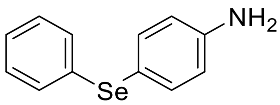

Following general procedure, **3af** was obtained as colorless soild in 88% yield (21.9 mg) from 4-iodoaniline (**1af**, 21.9 mg) and Diphenyl diselenide（**2e**, 15.6mg）using 10:1 petroleum ether/EtOAc as eluent. ^{1}H NMR (400 MHz, Chloroform-d) δ 7.42 – 7.38 (m, 2H), 7.30 – 7.26 (m, 2H), 7.21 – 7.13 (m, 3H), 6.66 – 6.60 (m, 2H), 3.76 (s, 2H).^{13}C NMR (101 MHz, Chloroform-d) δ 146.86, 137.14, 134.12, 130.15, 129.06, 126.05, 116.45, 116.04. The NMR data were in consistent with the reported data.[24]

4-(phenylselanyl)phenol (3ag)

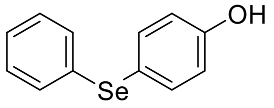

Following general procedure, **3ag** was obtained as colorless soild in 92% yield (23.0 mg) from 4-iodophenol (**1ag**, 22.0 mg) and Diphenyl diselenide（**2e**, 15.6mg）using 10:1 petroleum ether/EtOAc as eluent. ^{1}H NMR (400 MHz, Chloroform-d) δ 7.48 – 7.43 (m, 2H), 7.36 – 7.31 (m, 2H), 7.21 (dtd, J = 11.5, 5.8, 5.2, 2.2 Hz, 3H), 6.81 – 6.76 (m, 2H), 5.03 (s, 1H). ^{13}C NMR (101 MHz, Chloroform-d) δ 155.8, 136.7, 133.1, 131.0, 129.2, 126.6, 120.2, 116.6. The NMR data were in consistent with the reported data.[25]

(4-nitrophenyl)(phenyl)selane (3ah)

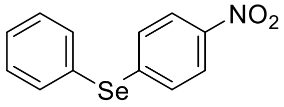

Following general procedure, **3ah** was obtained as yellow oil in 65% yield (18.1 mg) from 1-iodo-4-nitrobenzene (**1ah**, 24.9 mg) and Diphenyl diselenide（**2e**, 15.6mg）using 100:1 petroleum ether/EtOAc as eluent. ^{1}H NMR (400 MHz, Chloroform-d) δ 8.06 – 8.01 (m, 2H), 7.66 – 7.61 (m, 2H), 7.46 – 7.40 (m, 3H), 7.37 – 7.34 (m, 2H).^{13}C NMR (101 MHz, Chloroform-d) δ = 146.2, 143.9, 135.9, 130.1, 129.7, 129.4, 127.2, 124.0. The NMR data were in consistent with the reported data.[26]

4-(phenylselanyl)benzonitrile (3ai)

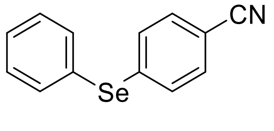

Following general procedure, **3ai** was obtained as colorless oil in 49% yield (12.6 mg) from 4-lodobenzonitrile (**1ai**, 22.9 mg) and Diphenyl diselenide（**2e**, 15.6mg）using 50:1 petroleum ether/EtOAc as eluent. ^{1}H NMR (400 MHz, Chloroform-d) δ 7.65–7.53 (m, 2H), 7.43 (t, *J*=5.5 Hz, 3H), 7.39 (dd, *J*=7.5, 4.5 Hz, 2H), 7.35–7.29 (m, 2H). ^{13}C NMR (101 MHz, Chloroform-d) δ 141.0, 135.7, 132.4, 130.3, 130.0, 129.2, 127.5, 118.8, 109.6. The NMR data were in consistent with the

reported data.[27]

methyl 4-(phenylselanyl)benzoate (3aj)

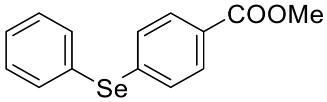

Following general procedure, **3aj** was obtained as colorless solid in 59% yield (17.3 mg) from 4-iodobenzoate (**1aj**, 26.2 mg) and Diphenyl diselenide（**2e**, 15.6mg）using 50:1 petroleum ether/EtOAc as eluent. ^{1}H NMR (400 MHz, Chloroform-d) δ 7.87 (d, *J*=8.4 Hz, 2H), 7.58 (dd, *J*=7.6, 1.8 Hz, 2H), 7.37 (dd, *J*=10.3, 4.0 Hz, 5H), 3.89 (s, 3H). ^{13}C NMR (101 MHz, Chloroform-d) δ 166.8, 139.7, 134.9, 130.4, 130.2, 129.7, 128.7, 128.5, 128.2, 52.1. The NMR data were in consistent with the reported data.[27]

4-(phenylselanyl)pyridine (3ak)

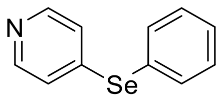

Following general procedure, **3ak** was obtained as colorless oil in 73% yield (17.2 mg) from 4-iodopyridine (**1ak**, 20.5 mg) and Diphenyl diselenide（**2e**, 15.6mg）using 50:1 petroleum ether/EtOAc as eluent. ^{1}H NMR (400 MHz, Chloroform-d) δ 8.34 – 8.29 (m, 2H), 7.68 – 7.61 (m, 2H), 7.47 – 7.38 (m, 3H), 7.10 (dd, J = 4.7, 1.4 Hz, 2H).^{13}C NMR (101 MHz, Chloroform-d) δ 149.6, 145.8, 136.3, 130.0, 129.4, 126.2, 123.9. The NMR data were in consistent with the reported data.[28]

naphthalen-1-yl(phenyl)selane (3al)

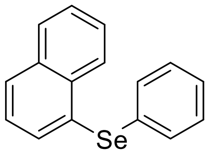

Following general procedure, **3al** was obtained as colorless oil in 62% yield (17.7 mg) from 1-iodonaphthalene (**1al**, 25.4mg) and Diphenyl diselenide（**2e**, 15.6mg）using petroleum ether as

eluent. ^1H NMR (400 MHz, Chloroform-d) δ 8.36 – 8.30 (m, 1H), 7.85 – 7.80 (m, 2H), 7.78 – 7.73 (m, 1H), 7.52 – 7.46 (m, 2H), 7.37 – 7.31 (m, 3H), 7.20 – 7.15 (m, 3H). ^{13}C NMR (101 MHz, Chloroform-d) δ 134.2, 133.9, 131.8, 129.3, 128.6, 127.7, 126.9, 126.4, 126.1. The NMR data were in consistent with the reported data.[29]

4-(methylselanyl)phenol (3am)

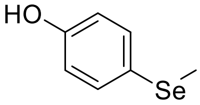

Following general procedure, **3am** was obtained as colorless soild in 92% yield (17.3 mg) from 4-iodophenol (**1am**, 22.0 mg) and Dimethyl diselenide (**2f**, 9.4mg) using 10:1 petroleum ether/EtOAc as eluent. ^1H NMR (400 MHz, Chloroform-d) δ 7.37 (d, J = 8.4 Hz, 2H), 6.76 (d, J = 8.4 Hz, 2H), 5.30 (s, 1H), 2.30 (s, 3H). ^{13}C NMR (101 MHz, Chloroform-d) δ 154.8, 133.7, 121.6, 116.3, 8.7. The NMR data were in consistent with the reported data.[22]

phenyl(p-tolyl)tellane (3an)

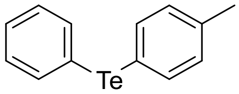

Following general procedure, **3an** was obtained as yellow oil in 80% yield (23.9 mg) from 4-iodobenzonitrile (**1an**, 21.8 mg) and Diphenyl ditellane (**2g**, 20.5mg) using 100:1 petroleum ether/EtOAc as eluent. ^1H NMR (400 MHz, Chloroform-d) δ 7.63 (dq, J = 6.9, 1.5 Hz, 4H), 7.23 – 7.17 (m, 3H), 7.05 (d, J = 7.8 Hz, 2H), 2.34 (s, 3H). ^{13}C NMR (101 MHz, Chloroform-d) δ 138.78, 138.10, 137.28, 130.45, 129.37, 127.44, 115.23, 110.23, 21.34. The NMR data were in consistent with the reported data.[30]

4-(phenyltellanyl)benzonitrile (3ao)

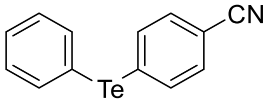

Following general procedure, **3ao** was obtained as yellow oil in 73% yield (22.6 mg) from 4-

iodobenzonitrile (**1ao**, 22.9 mg) and Diphenyl ditellane (**2g**, 20.5mg) using 50:1 petroleum ether/EtOAc as eluent. ^{1}H NMR (400 MHz, Chloroform-d) δ 7.87 – 7.81 (m, 2H), 7.58 – 7.53 (m, 2H), 7.45 – 7.37 (m, 3H), 7.32 (dd, J = 8.2, 6.8 Hz, 2H). ^{13}C NMR (101 MHz, Chloroform-d) δ 140.20, 135.74, 132.34, 130.16, 129.29, 124.50, 118.87, 112.83, 110.72. The NMR data were in consistent with the reported data.[30]

4-((2,2,6,6-tetramethylpiperidin-1-yl)oxy)phenol (4a)

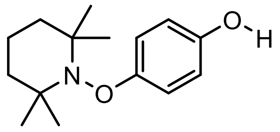

Following procedure below, **4a** was obtained as yellow oil in 9% yield (22.5 mg). A 10 mL reaction quartz tube was charged with a magnetic stir bar, **1e** (22.0 mg, 0.1 mmol), TMG (0.05 mmol), PhSSPh (0.05 mmol), TEMPO (0.2 mmol) and 1 mL EA. The tube was sealed with a screw cap and put into the photoreactor for 12 h. After then, it was added water and ethyl acetate to dilute the organic phase. After shaking for 1 min, the organic layer was separated and analyzed using 10:1 petroleum ether/EtOAc as eluent. ^{1}H NMR (400 MHz, Chloroform-d) δ 7.70 – 7.64 (m, 2H), 7.47 – 7.41 (m, 2H), 1.66 (d, J = 7.8 Hz, 6H), 1.52 (d, J = 25.8 Hz, 8H), 1.29 (d, J = 19.7 Hz, 2H), 0.89 (s, 2H). ^{13}C NMR (101 MHz, Chloroform-d) δ 150.28, 129.40, 128.61, 126.06, 61.38, 58.95, 43.54, 41.46, 35.44, 32.68, 28.04, 17.36. HRMS (ESI) m/z: [M + H]+ Calcd. for $C_{16}H_{14}N_2O_2$: 249.1729; Found 249.1733.

6. Mechanism of the Sulfuration Reaction

6.1 On-Off Experiment

In order to better understand the effect of light in this radical sulfuration reaction. On-off experiment was carried out. No product formation was observed during the dark periods in this experiment, it demonstrated that light is important for this sulfuration reaction.

A 10 mL reaction quartz tube was charged with a magnetic stir bar, **1e** (22.0 mg, 0.1 mmol), TMG (0.05 mmol), PhSSPh(0.05 mmol) and 1 mL EA. The tube was sealed with a screw cap and put into the photoreactor for 12 h. After then, it was added water and ethyl acetate to dilute the organic phase. After shaking for 1 min, the organic layer was separated and analyzed by GC.

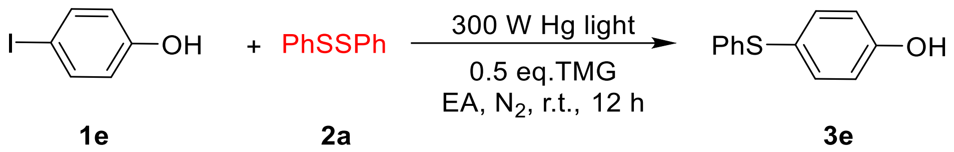

Table S3. Raw data of GC yields in on-off experiment.

Time/h	0	2	4	6	8	10	12	16	20	24	28
Yield/%	0	17	17	27	27	35	35	62	62	87	87

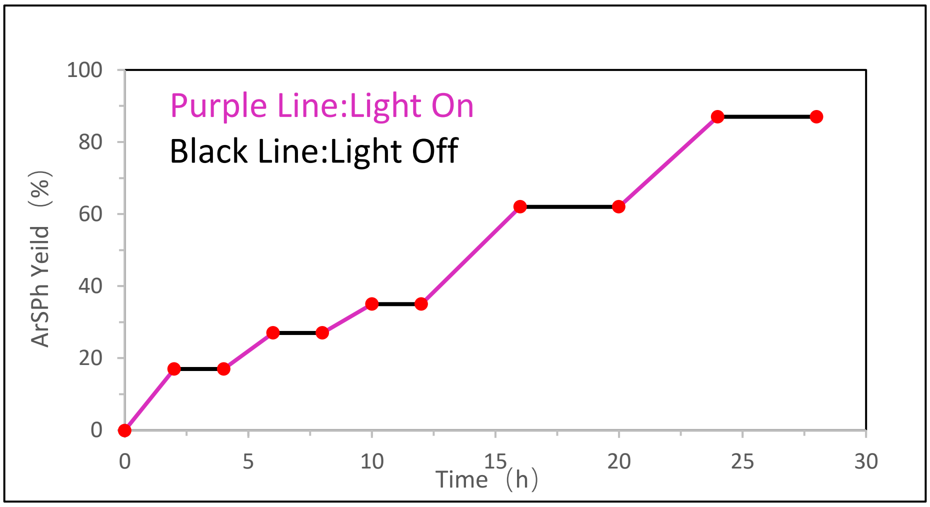

Figure S2. On-off experiment kinetic profile.

6.2 TEMPO Capture Experiment

A 10 mL reaction quartz tube was charged with a magnetic stir bar, **1e** (22.0 mg, 0.1 mmol), TMG (0.05 mmol), PhSSPh (0.05 mmol), TEMPO (0.2 mmol) and 1 mL EA. The tube was sealed with a screw cap and put into the photoreactor for 12 h. After then, it was added water and ethyl acetate to dilute the organic phase. After shaking for 1 min, the organic layer was separated and analyzed by LC-MS.

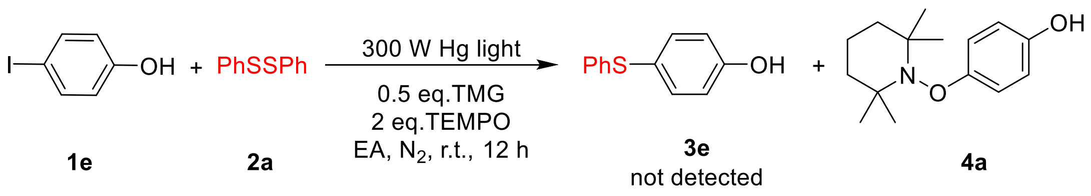

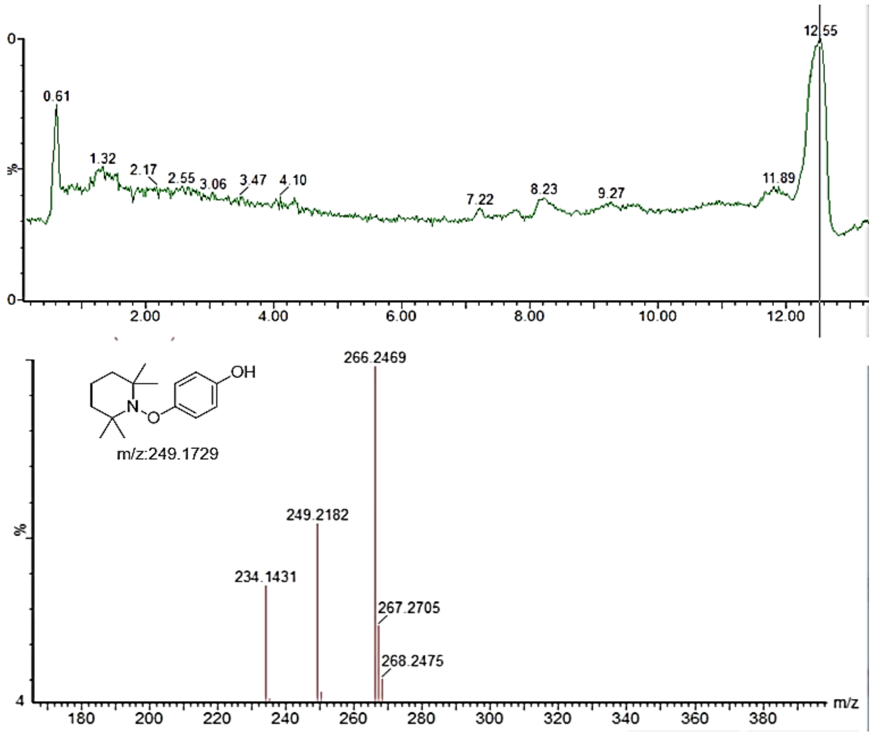

Figure S3. TEMPO Capture Experiment Result.

6.3 I_2 Verification Experiment

According to standard conditions, the system was dark-brown after the reaction was completed. When an appropriate amount of sodium thiosulfate solution was added, the system became colourless and transparent.

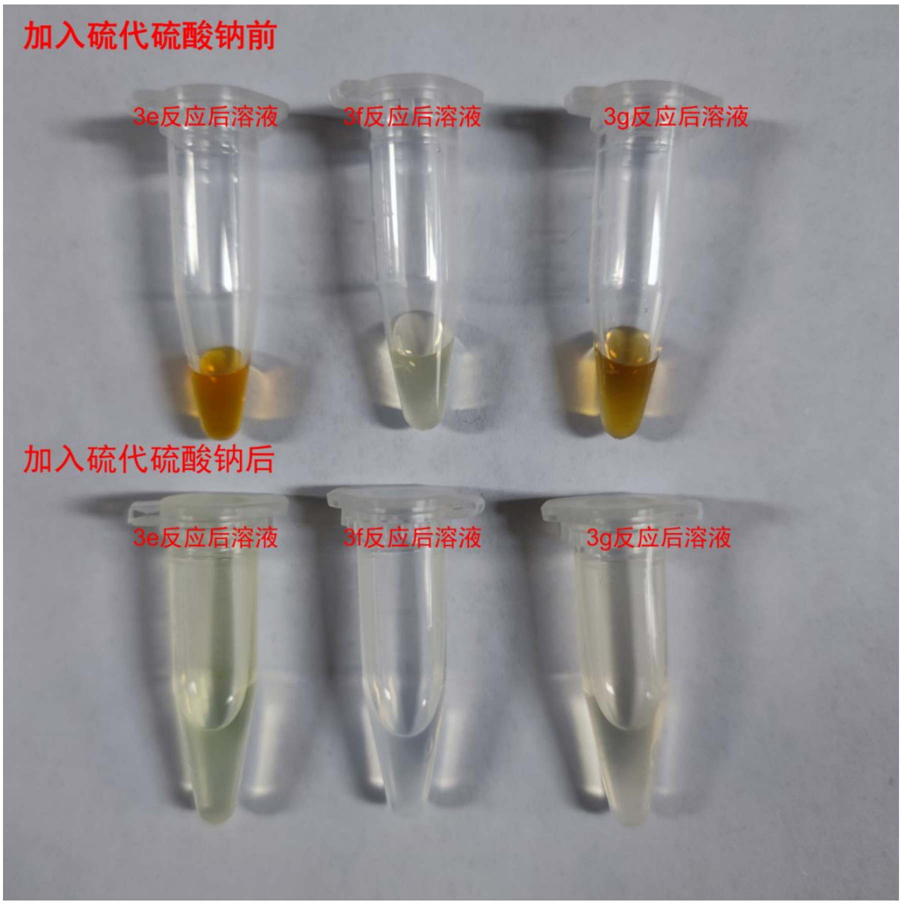

Figure S4. I_2 Verification Experiment Result.

7. References

[1] Lin, Y.-L.; Cheng, J.-Y.; Chu, Y.-H., Microwave-accelerated Claisen rearrangement in bicyclic imidazolium [b-3C-im][NTf2] ionic liquid. *Tetrahedron* **2007,** *63* (45), 10949-10957.

[2] Martín, M. T.; Marín, M.; Maya, C.; Prieto, A.; Nicasio, M. C., Ni(II) Precatalysts Enable Thioetherification of (Hetero)Aryl Halides and Tosylates and Tandem C−S/C−N Couplings. *Chemistry – A European Journal* **2021,** *27* (48), 12320-12326.

[3] Lin, Y.; Cai, M.; Fang, Z.; Zhao, H., A highly efficient heterogeneous copper-catalyzed Chan-Lam coupling between thiols and arylboronic acids leading to diaryl sulfides under mild conditions. *Tetrahedron* **2016,** *72* (23), 3335-3343.

[4] Samanta, P. K.; Biswas, R.; Bhaduri, S. N.; Ray, S.; Biswas, P., Copper(0) nanoparticles immobilized on SBA-15: A versatile recyclable heterogeneous catalyst for solvent and ligand free C–S coupling reaction from diverse substrates. *Microporous and Mesoporous Materials* **2021,** *323*, 111198.

[5] Lam, L. Y.; Ma, C., Chan–Lam-Type C–S Coupling Reaction by Sodium Aryl Sulfinates and Organoboron Compounds. *Organic Letters* **2021,** *23* (15), 6164-6168.

[6] Unglaube, F.; Kreyenschulte, C. R.; Mejía, E., Development and Application of Efficient Ag-

based Hydrogenation Catalysts Prepared from Rice Husk Waste. *ChemCatChem* **2021,** *13* (11), 2583-2591.

[7] Zhang, W.; Huang, M.; Zou, Z.; Wu, Z.; Ni, S.; Kong, L.; Zheng, Y.; Wang, Y.; Pan, Y., Redox-active benzimidazolium sulfonamides as cationic thiolating reagents for reductive cross-coupling of organic halides. *Chemical Science* **2021,** *12* (7), 2509-2514.

[8] Mitamura, K.; Yatabe, T.; Yamamoto, K.; Yabe, T.; Suzuki, K.; Yamaguchi, K., Heterogeneously Ni–Pd nanoparticle-catalyzed base-free formal C–S bond metathesis of thiols. *Chemical Communications* **2021,** *57* (31), 3749-3752.

[9] Li, Y.; Bao, G.; Wu, X.-F., Palladium-catalyzed intermolecular transthioetherification of aryl halides with thioethers and thioesters. *Chemical Science* **2020,** *11* (8), 2187-2192.

[10] Jin, L.; Zhang, P.; Li, Y.; Yu, X.; Shi, B.-F., Atroposelective Synthesis of Conjugated Diene-Based Axially Chiral Styrenes via Pd(II)-Catalyzed Thioether-Directed Alkenyl C–H Olefination. *Journal of the American Chemical Society* **2021,** *143* (31), 12335-12344.

[11] Dorval, C.; Tricoire, M.; Begouin, J.-M.; Gandon, V.; Gosmini, C., Cobalt-Catalyzed C(sp2)–CN Bond Activation: Cross-Electrophile Coupling for Biaryl Formation and Mechanistic Insight. *ACS Catalysis* **2020,** *10* (21), 12819-12827.

[12] Delcaillau, T.; Boehm, P.; Morandi, B., Nickel-Catalyzed Reversible Functional Group Metathesis between Aryl Nitriles and Aryl Thioethers. *Journal of the American Chemical Society* **2021,** *143* (10), 3723-3728.

[13] Wang, X.; Tang, Y.; Long, C.-Y.; Dong, W.-K.; Li, C.; Xu, X.; Zhao, W.; Wang, X.-Q., Nucleophilic Amination and Etherification of Aryl Alkyl Thioethers. *Organic Letters* **2018,** *20* (16), 4749-4753.

[14] Gutiérrez-Tarriño, S.; Rojas-Buzo, S.; Lopes, C. W.; Agostini, G.; Calvino, J. J.; Corma, A.; Oña-Burgos, P., Cobalt nanoclusters coated with N-doped carbon for chemoselective nitroarene hydrogenation and tandem reactions in water. *Green Chemistry* **2021,** *23* (12), 4490-4501.

[15] Zhang, B.; Fan, Z.; Guo, Z.; Xi, C., Reduction of CO2 with NaBH4/I2 for the Conversion of Thiophenols to Aryl Methyl Sulfides. *The Journal of Organic Chemistry* **2019,** *84* (13), 8661-8667.

[16] Li, H. L.; Kuninobu, Y.; Kanai, M., Lewis Acid–Base Interaction-Controlled ortho-Selective C−H Borylation of Aryl Sulfides. *Angewandte Chemie International Edition* **2017,** *56* (6), 1495-1499.

[17] Petsi, M.; Zografos, A. L., 2,5-Diketopiperazine Catalysts as Activators of Dioxygen in Oxidative Processes. *ACS Catalysis* **2020,** *10* (13), 7093-7099.

[18] Wang, M.; Qiao, Z.; Zhao, J.; Jiang, X., Palladium-Catalyzed Thiomethylation via a Three-Component Cross-Coupling Strategy. *Organic Letters* **2018,** *20* (19), 6193-6197.

[19] Yanagi, T.; Somerville, R. J.; Nogi, K.; Martin, R.; Yorimitsu, H., Ni-Catalyzed Carboxylation of C(sp2)–S Bonds with CO2: Evidence for the Multifaceted Role of Zn. *ACS Catalysis* **2020,** *10* (3), 2117-2123.

[20] Glasnov, T. N.; Holbrey, J. D.; Kappe, C. O.; Seddon, K. R.; Yan, T., Methylation using dimethylcarbonate catalysed by ionic liquids under continuous flow conditions. *Green Chemistry* **2012,** *14* (11), 3071-3076.

[21] Chakraborty, U.; Reyes-Rodriguez, E.; Demeshko, S.; Meyer, F.; Jacobi von Wangelin, A., A

Manganese Nanosheet: New Cluster Topology and Catalysis. *Angewandte Chemie International Edition* **2018,** *57* (18), 4970-4975.

[22] Fu, Z.; Yin, J.; He, D.; Yi, X.; Guo, S.; Cai, H., An electrochemical method for deborylative selenylation of arylboronic acids under metal- and oxidant-free conditions. *Green Chemistry* **2022,** *24* (1), 130-135.

[23] Sun, N.; Zheng, K.; Sun, P.; Chen, Y.; Jin, L.; Hu, B.; Shen, Z.; Hu, X., Trichloroisocyanuric Acid-Promoted Synthesis of Arylselenides and Aryltellurides from Diorganyl Dichalcogenides and Arylboronic Acids at Ambient Temperature. *Advanced Synthesis & Catalysis* **2021,** *363* (14), 3577-3584.

[24] Ielo, L.; Castoldi, L.; Touqeer, S.; Lombino, J.; Roller, A.; Prandi, C.; Holzer, W.; Pace, V., Halogen-Imparted Reactivity in Lithium Carbenoid Mediated Homologations of Imine Surrogates: Direct Assembly of bis-Trifluoromethyl-β-Diketiminates and the Dual Role of LiCH2I. *Angewandte Chemie International Edition* **2020,** *59* (47), 20852-20857.

[25] Saba, S.; Rafique, J.; Braga, A. L., DMSO/iodine-catalyzed oxidative C–Se/C–S bond formation: a regioselective synthesis of unsymmetrical chalcogenides with nitrogen- or oxygen-containing arenes. *Catalysis Science & Technology* **2016,** *6* (9), 3087-3098.

[26] Liu, J.; Tian, M.; Li, Y.; Shan, X.; Li, A.; Lu, K.; Fagnoni, M.; Protti, S.; Zhao, X., Metal-Free Synthesis of Unsymmetrical Aryl Selenides and Tellurides via Visible Light-Driven Activation of Arylazo Sulfones. *European Journal of Organic Chemistry* **2020,** *2020* (47), 7358-7367.

[27] Bai, J.-H.; Qi, X.-J.; Sun, W.; Yu, T.-Y.; Xu, P.-F., Nickel-Catalyzed Intramolecular Decarbonylative Coupling of Aryl Selenol Esters. *Advanced Synthesis & Catalysis* **2021,** *363* (8), 2084-2088.

[28] Mandal, T.; Das, S.; De Sarkar, S., Nickel(II) Tetraphenylporphyrin as an Efficient Photocatalyst Featuring Visible Light Promoted Dual Redox Activities. *Advanced Synthesis & Catalysis* **2019,** *361* (13), 3200-3209.

[29] Pan, L.; Cooke, M. V.; Spencer, A.; Laulhé, S., Dimsyl Anion Enables Visible-Light-Promoted Charge Transfer in Cross-Coupling Reactions of Aryl Halides. *Advanced Synthesis & Catalysis* **2022,** *364* (2), 420-425.

[30] Kumar, A.; Kumar, S., A convenient and efficient copper-catalyzed synthesis of unsymmetrical and symmetrical diaryl chalcogenides from arylboronic acids in ethanol at room temperature. *Tetrahedron* **2014,** *70* (9), 1763-1772.

8. NMR Spectra for the Products

1d ^{1}H NMR

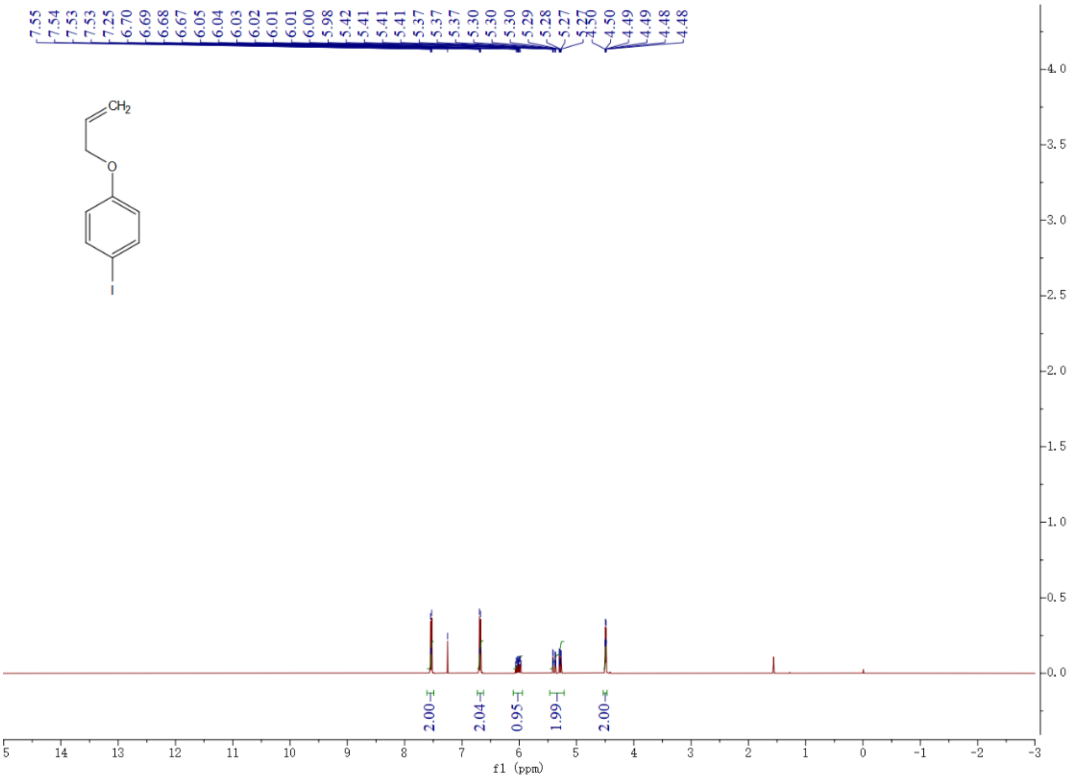

1d ^{13}C NMR

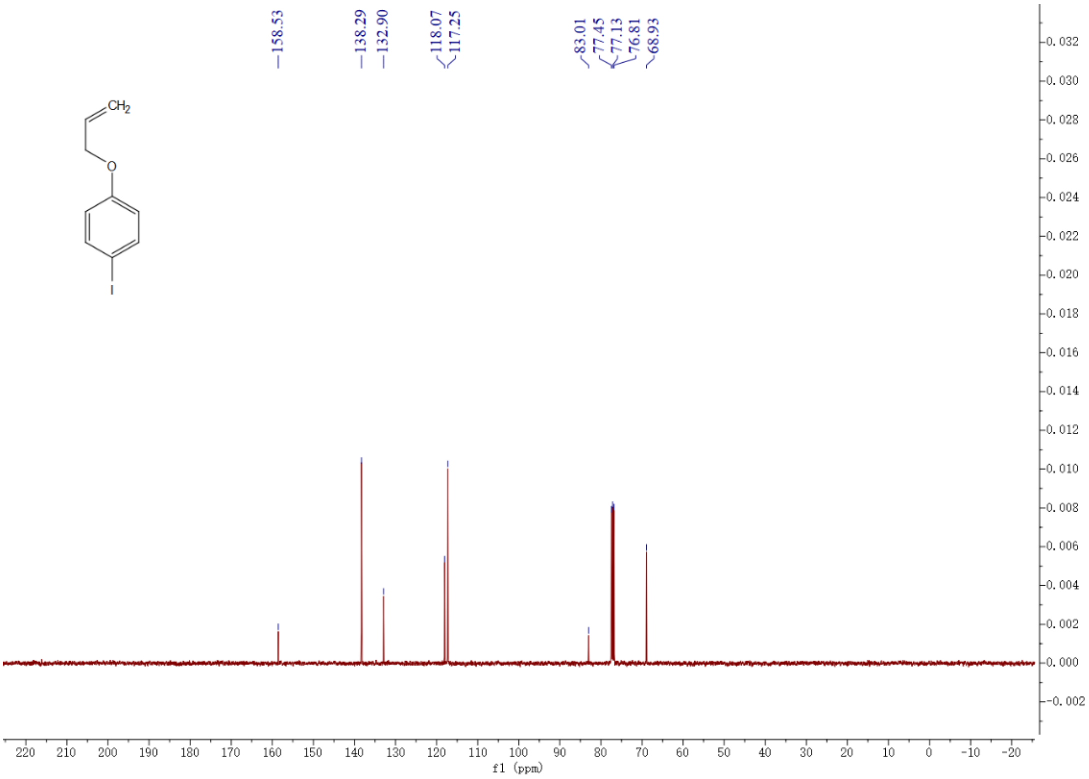

3a ^{1}H NMR

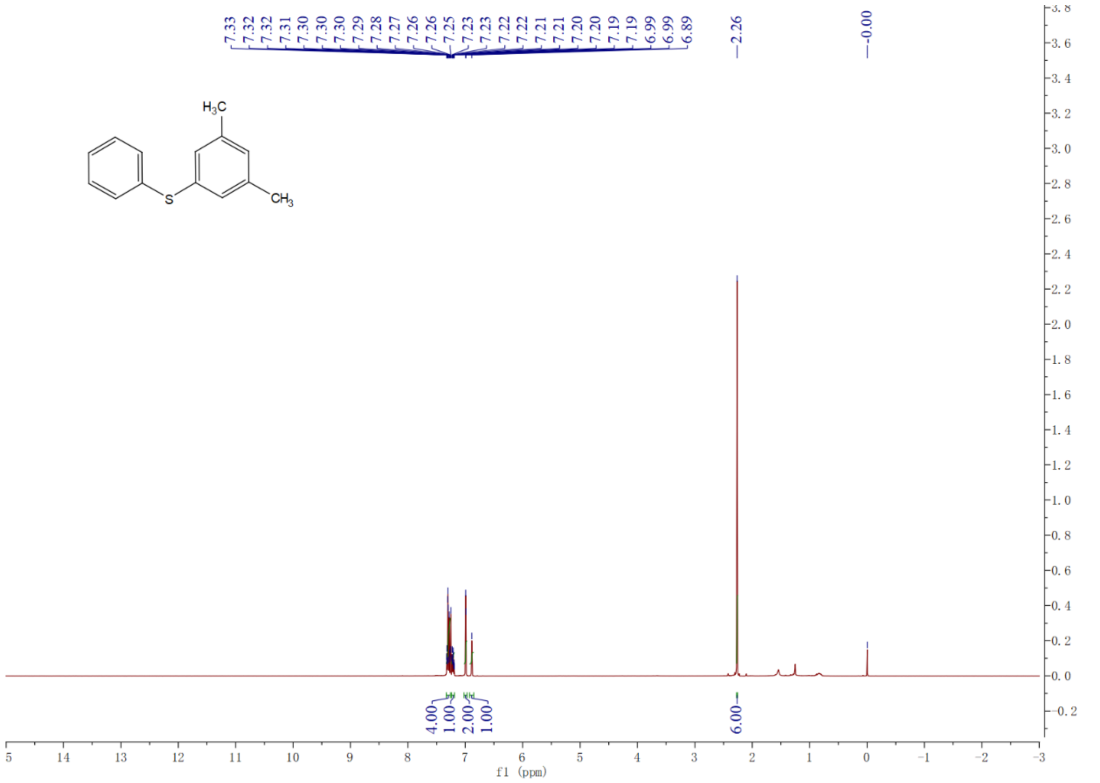

3a ^{13}C NMR

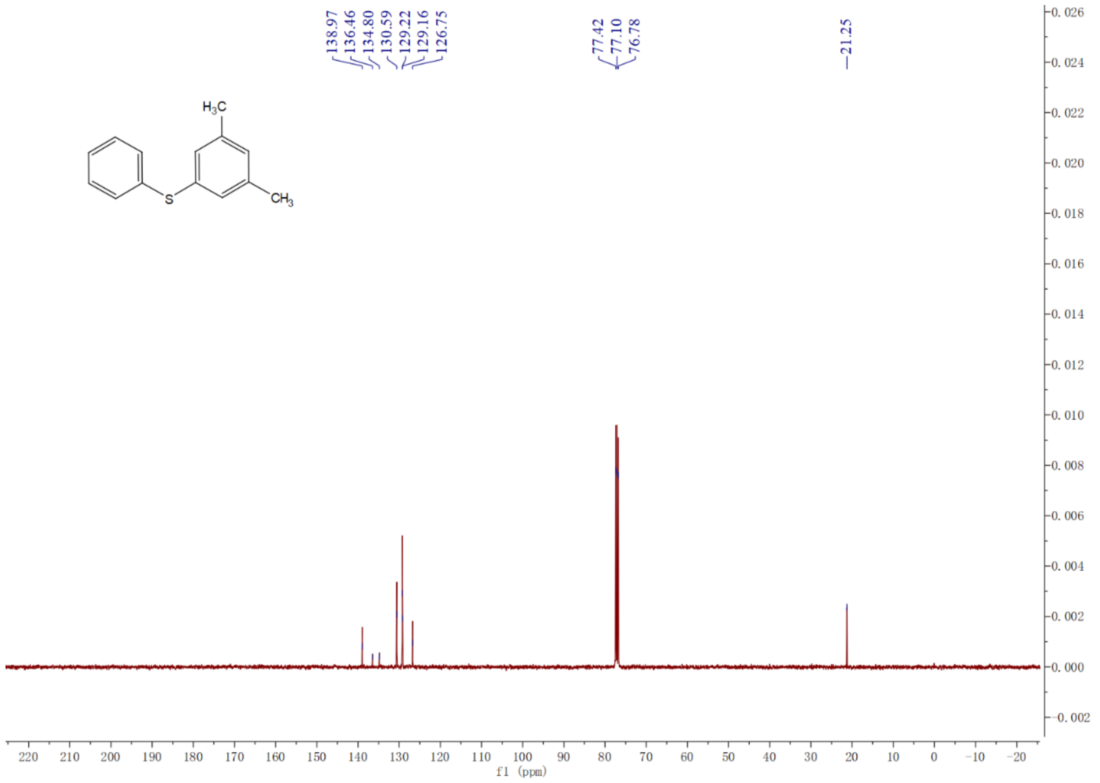

3b ^{1}H NMR

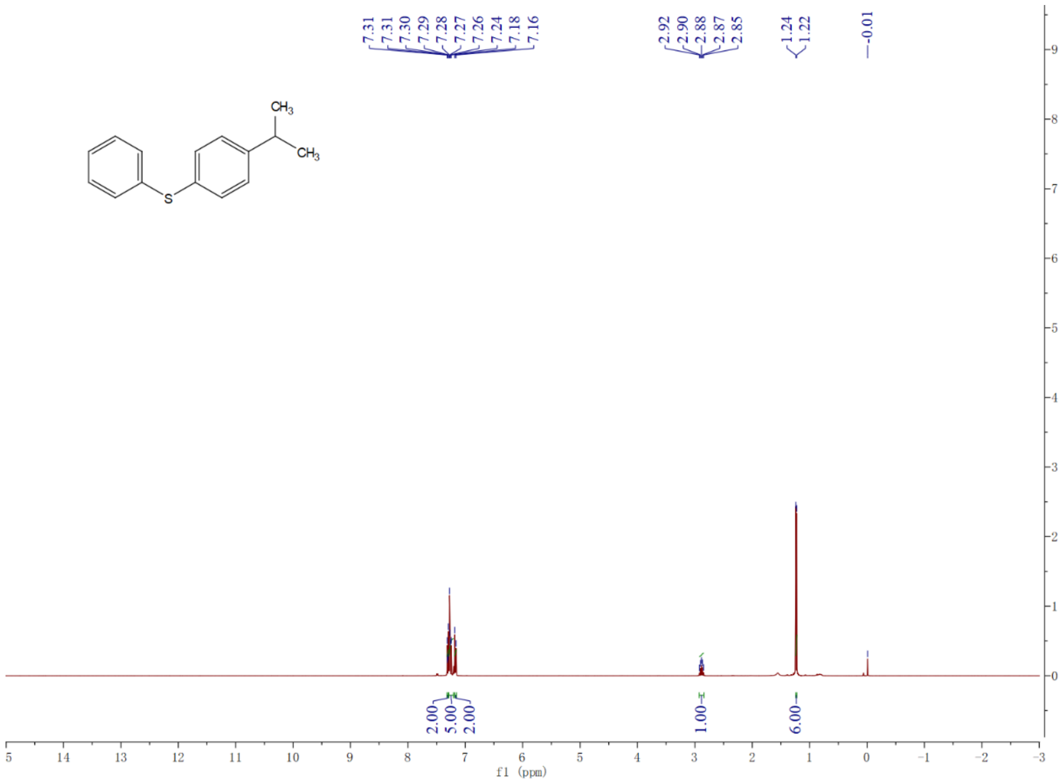

3b ^{13}C NMR

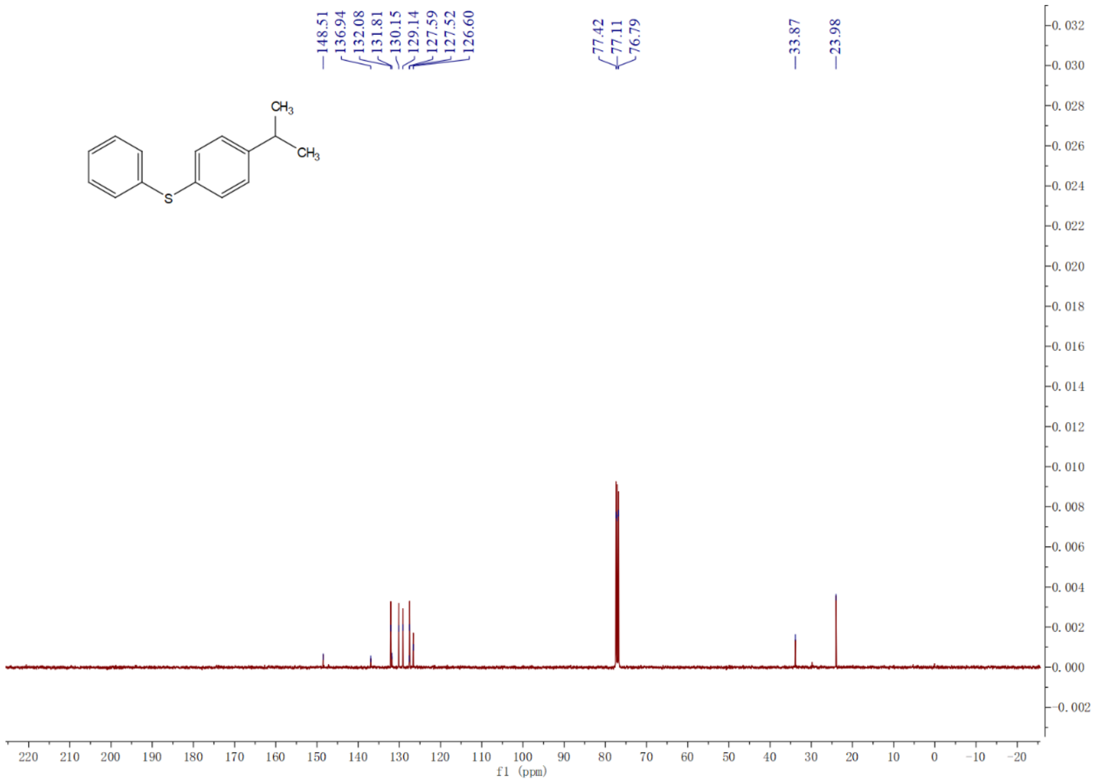

3c ^{1}H NMR

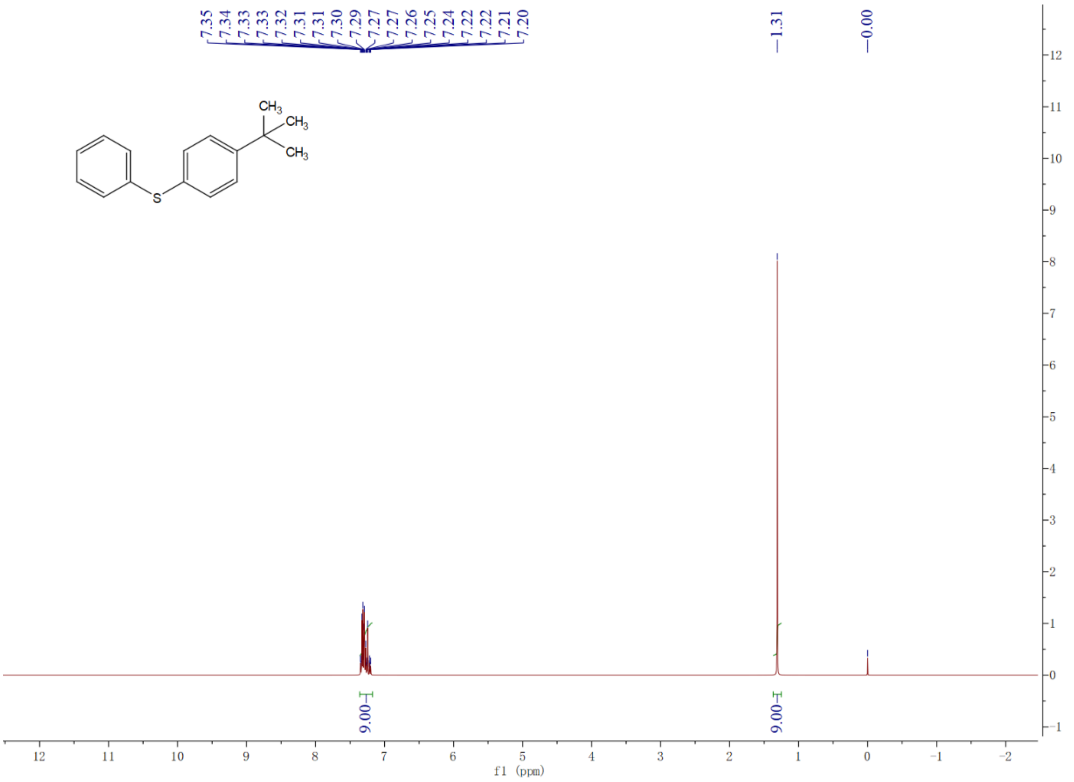

3c ^{13}C NMR

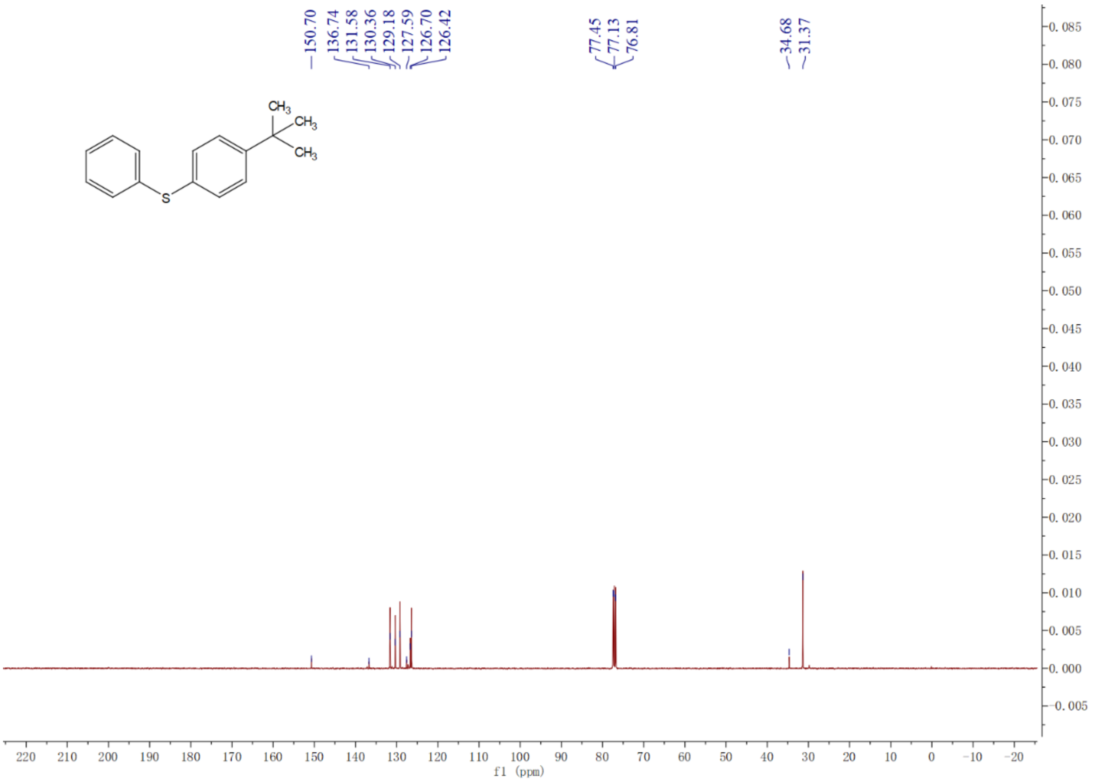

3d ^{1}H NMR

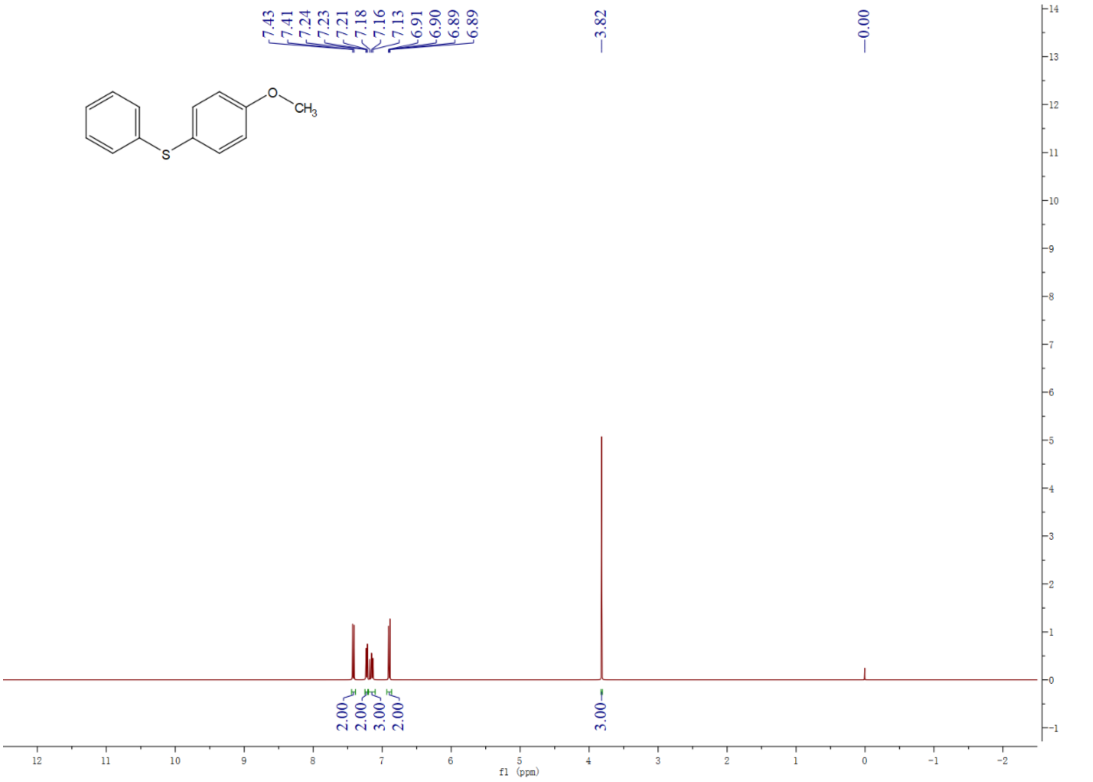
7.43
7.41
7.24
7.23
7.21
7.18
7.16
7.13
6.91
6.90
6.89
6.89
3.82
0.00
S
O
CH3
2.00
2.00
3.00
2.00
3.00
f1 (ppm)

3d ^{13}C NMR

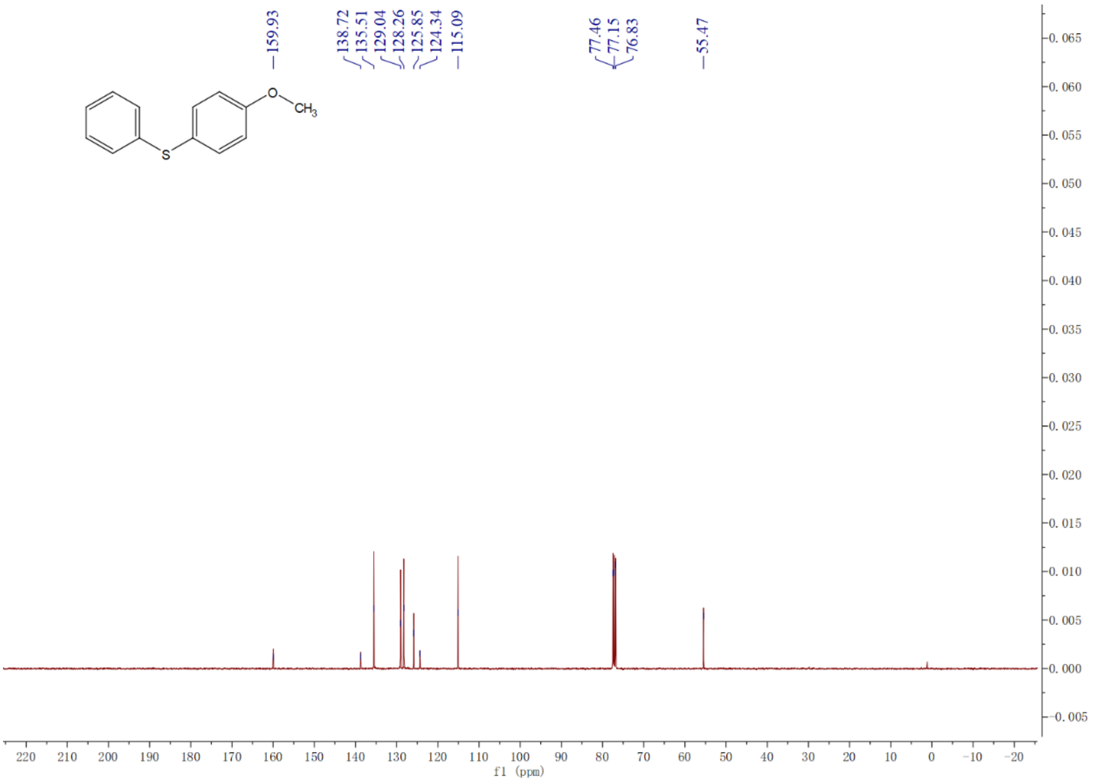
159.93
138.72
135.51
129.04
128.26
125.85
124.34
115.09
77.46
77.15
76.83
55.47
S
O
CH3
f1 (ppm)

3e ^{1}H NMR

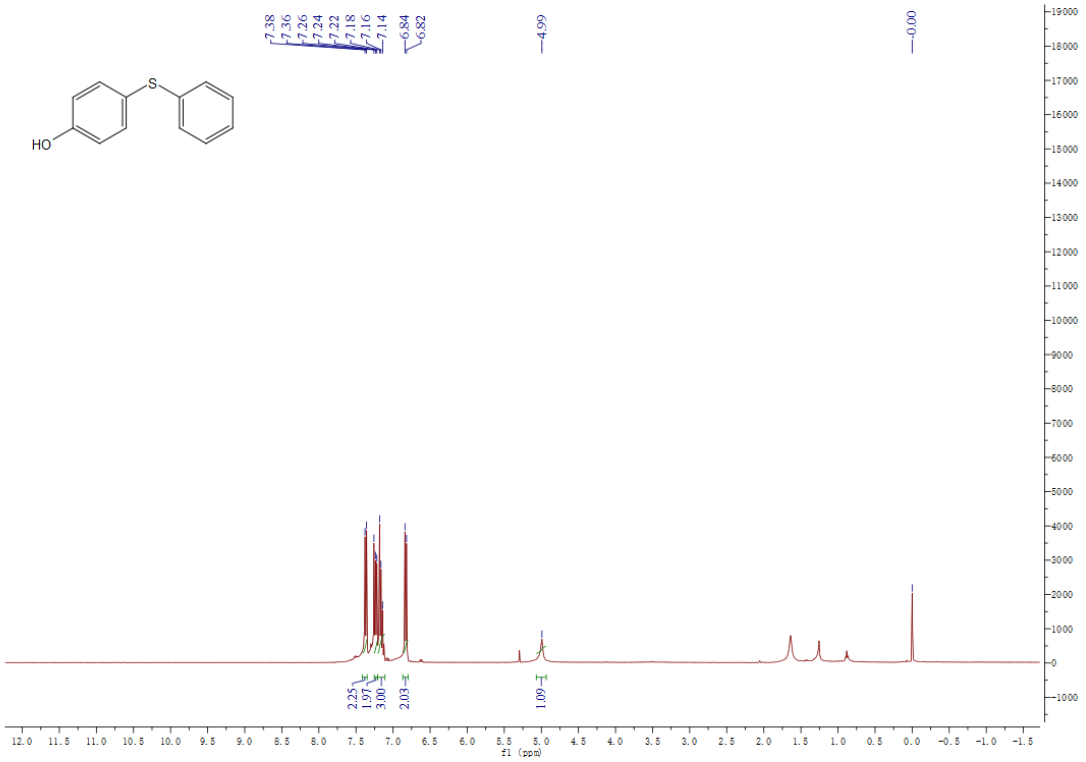

3e ^{13}C NMR

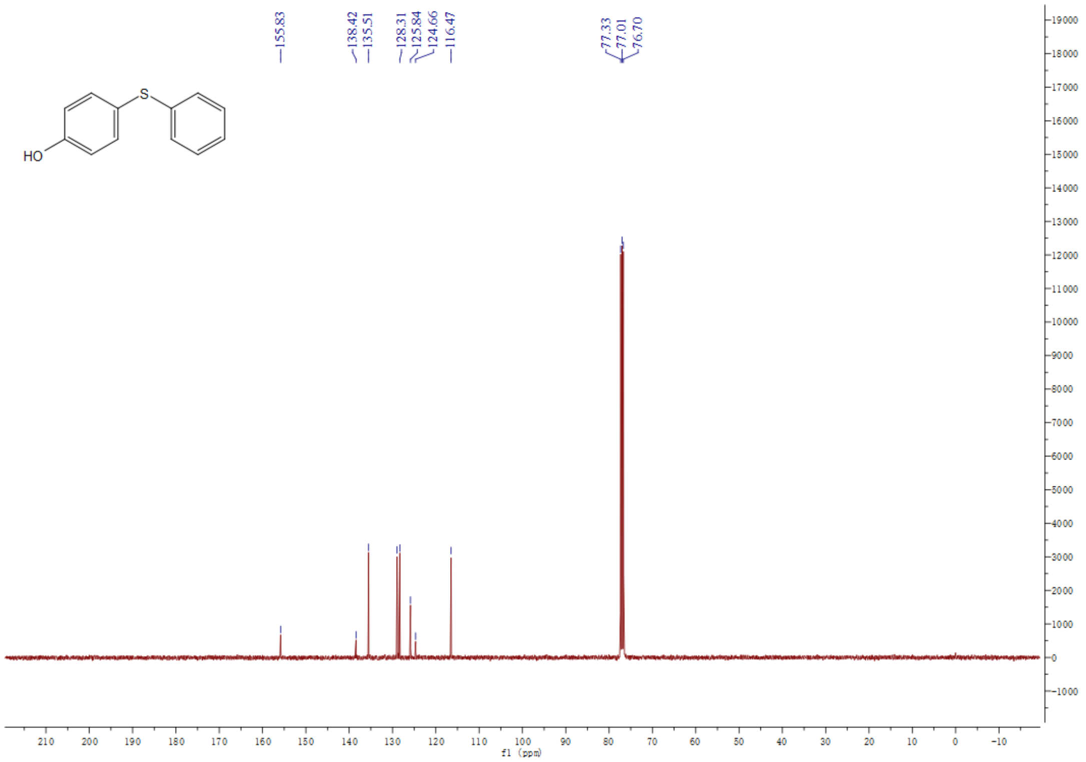

3f ^{1}H NMR

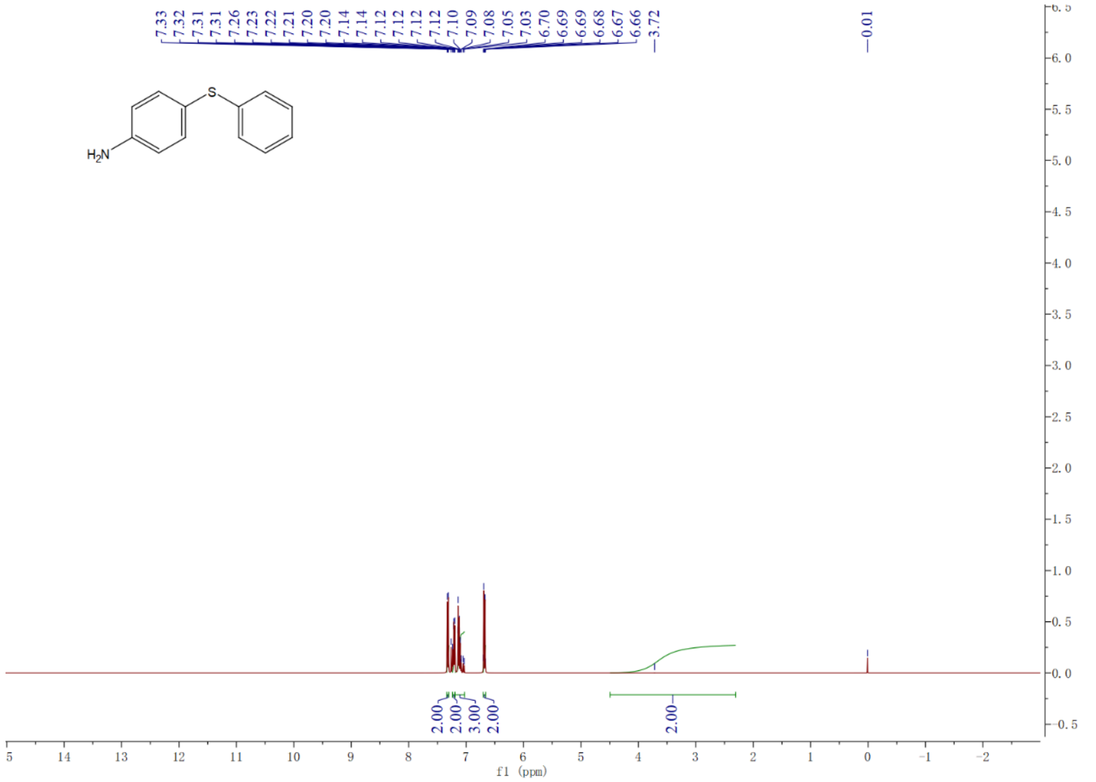

3f ^{13}C NMR

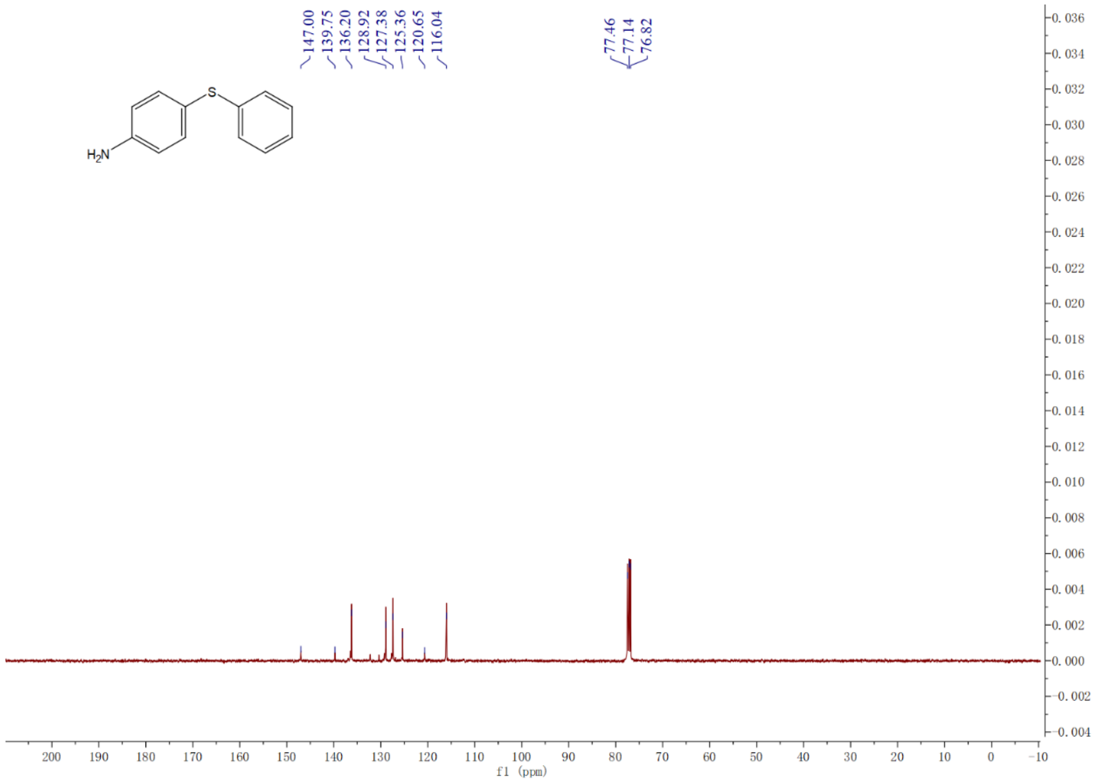

3g ^{1}H NMR

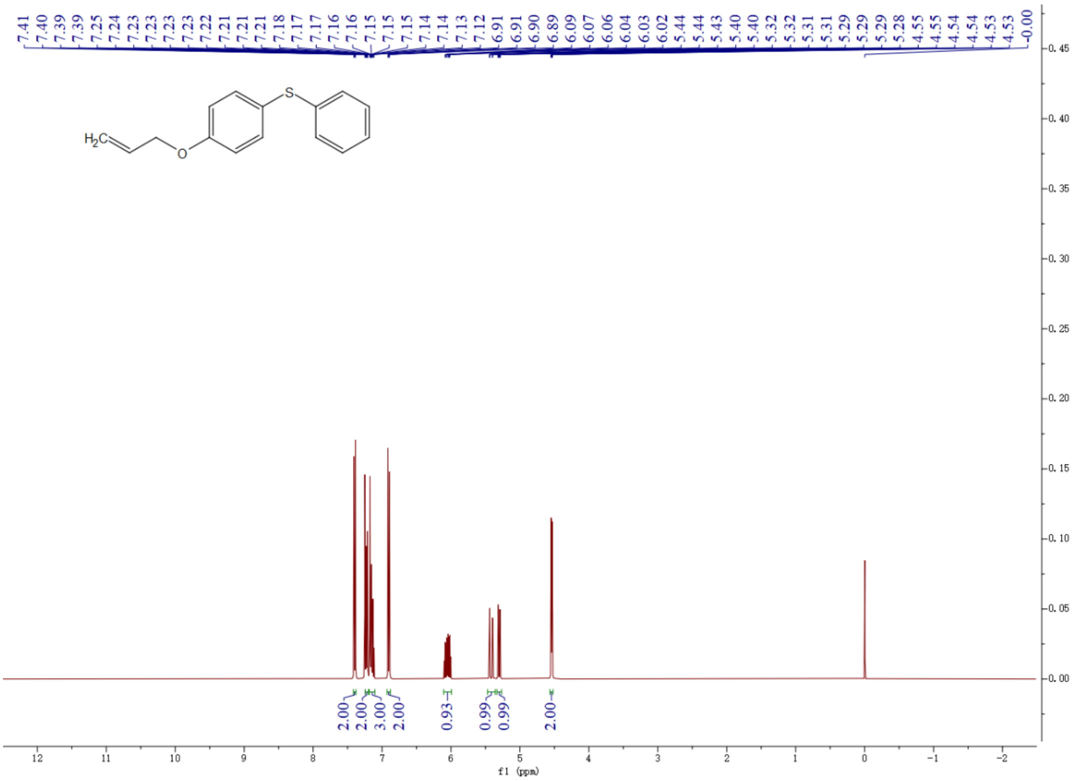

3g ^{13}C NMR

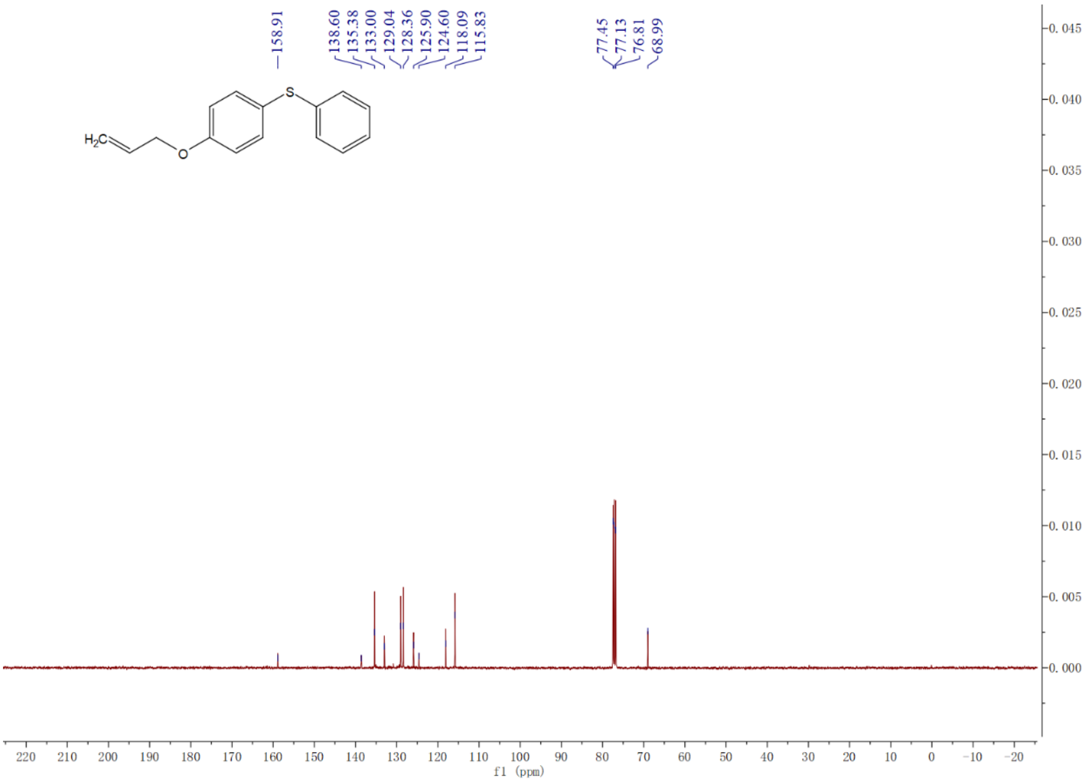

3h ^{1}H NMR

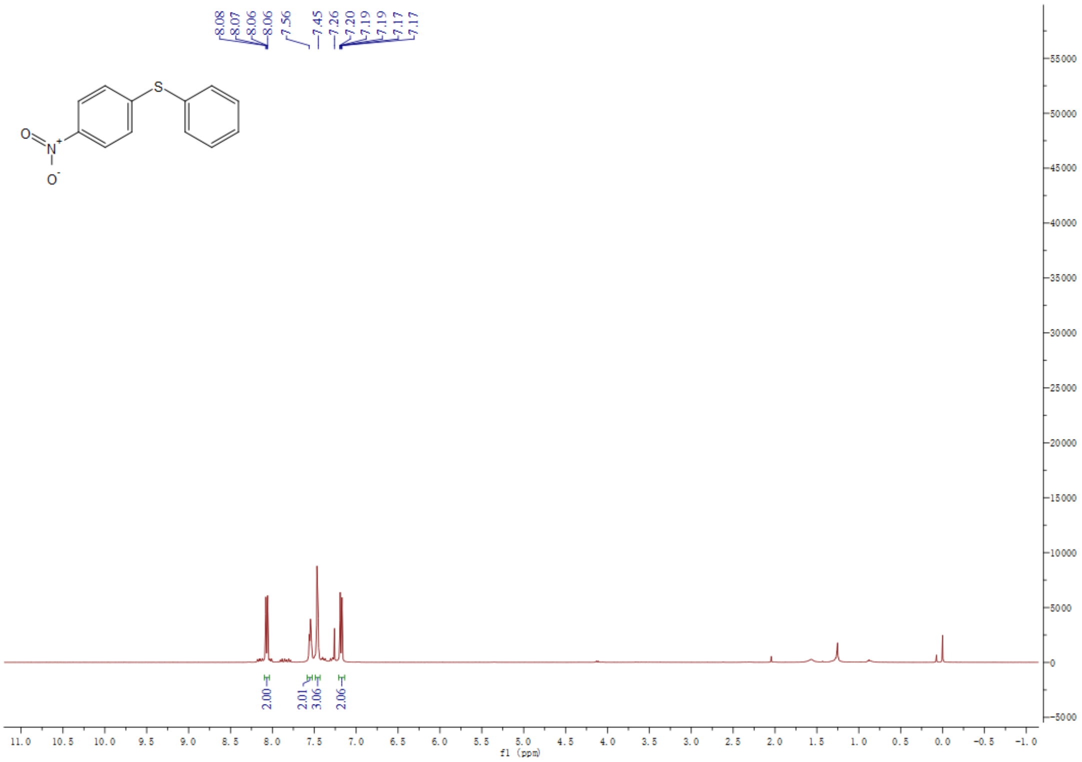

3h ^{13}C NMR

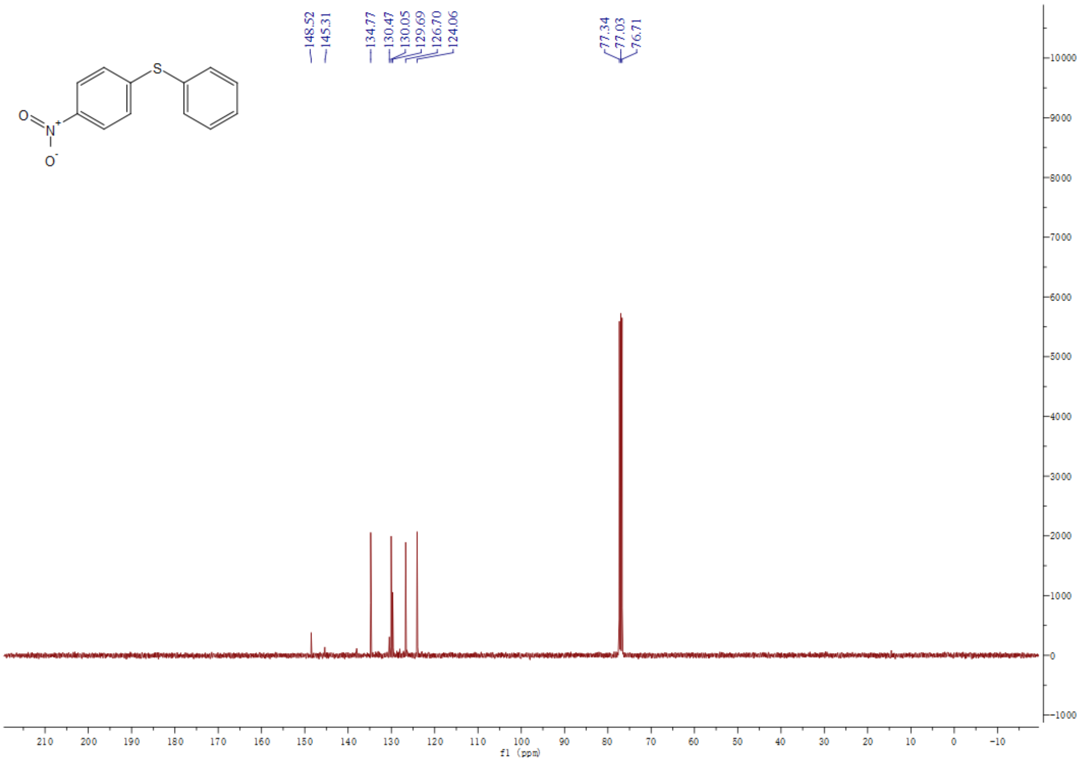

3i ^{1}H NMR

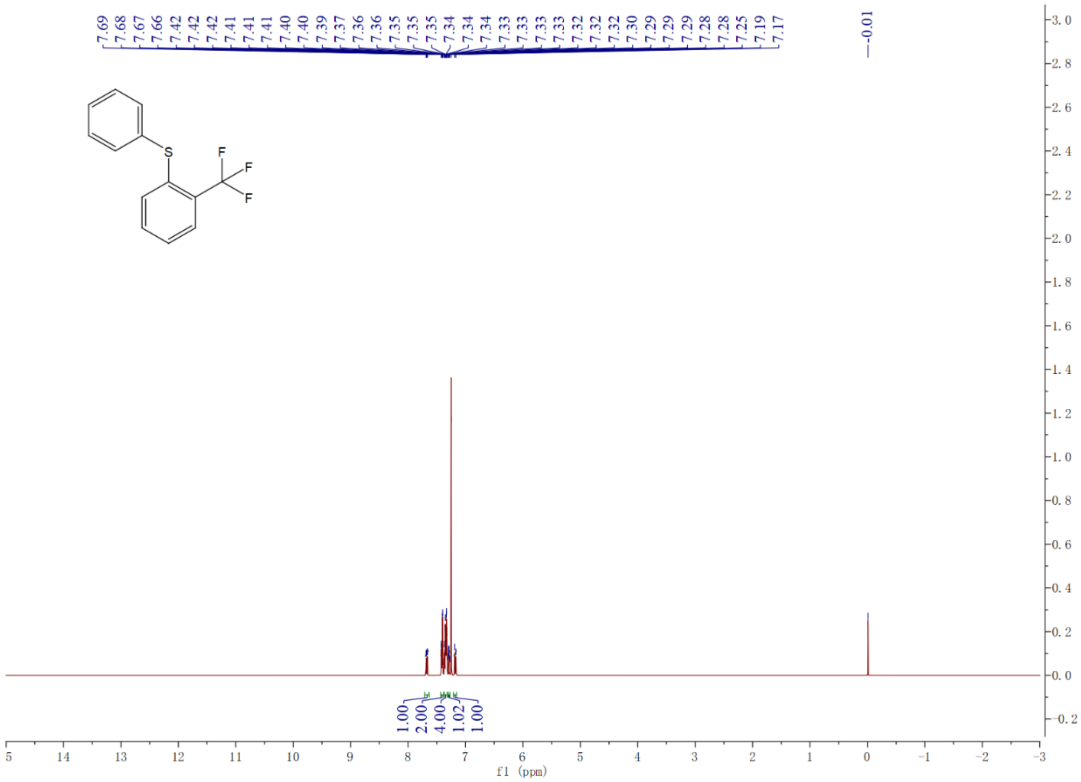

3i ^{13}C NMR

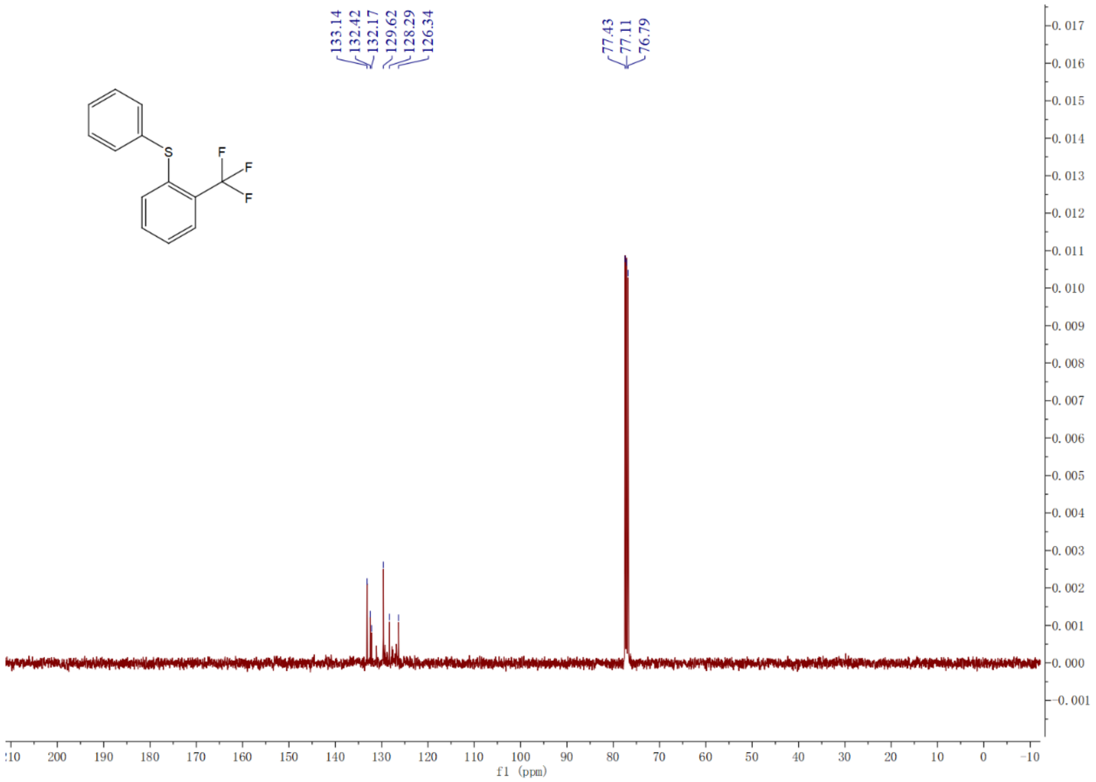

3aa ^{1}H NMR

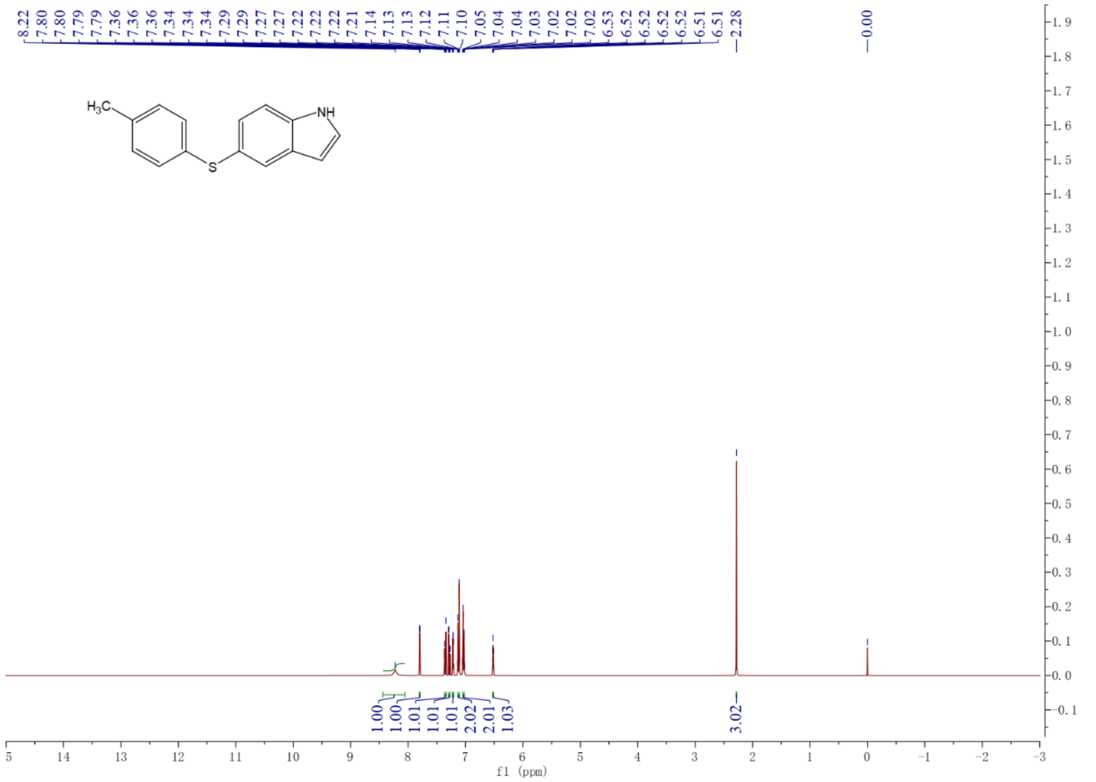

3aa ^{13}C NMR

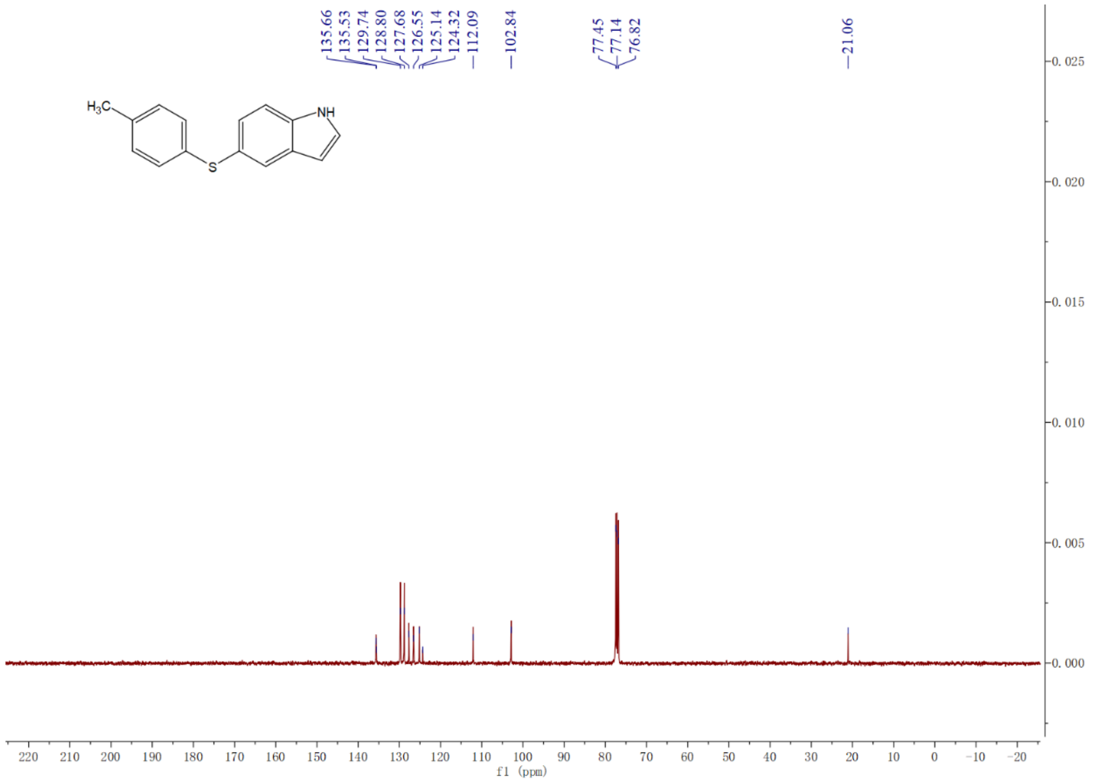

3ab ^{1}H NMR

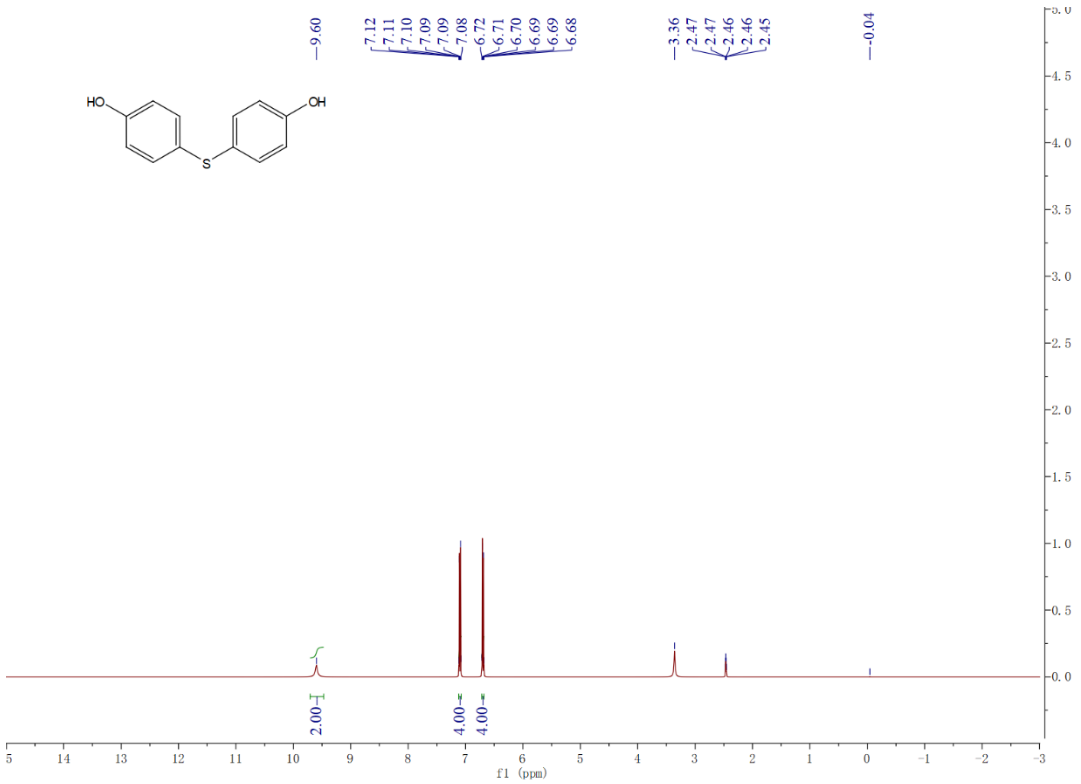

3ab ^{13}C NMR

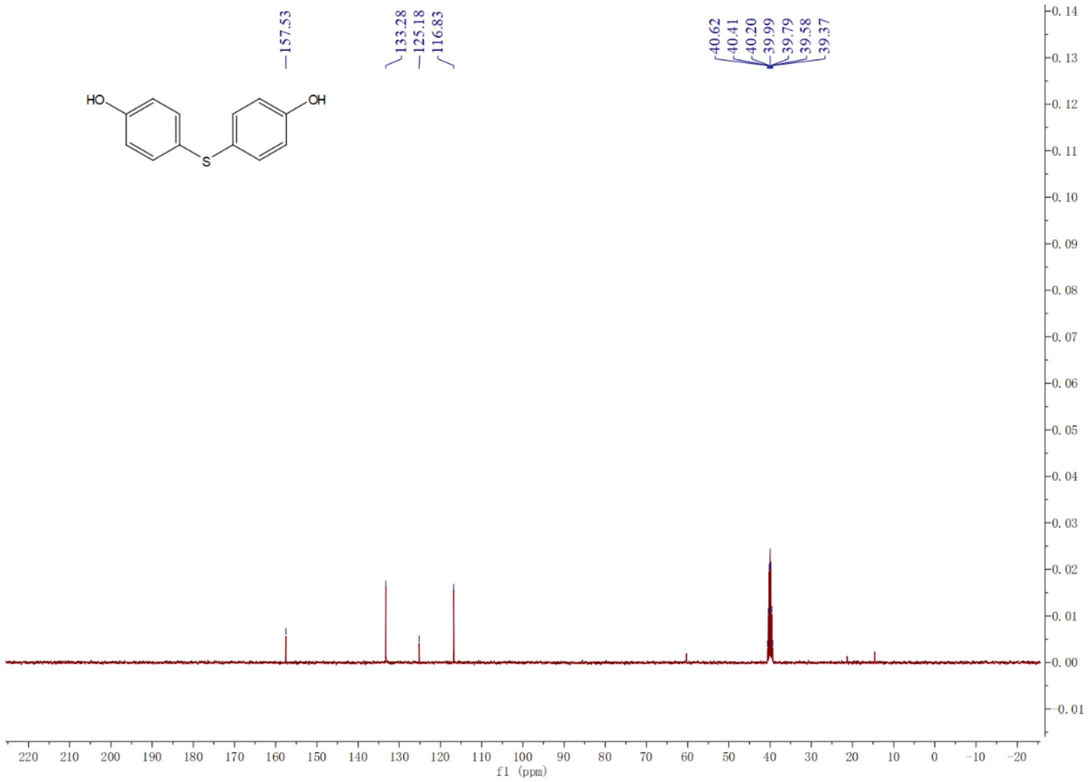

3j ^{1}H NMR

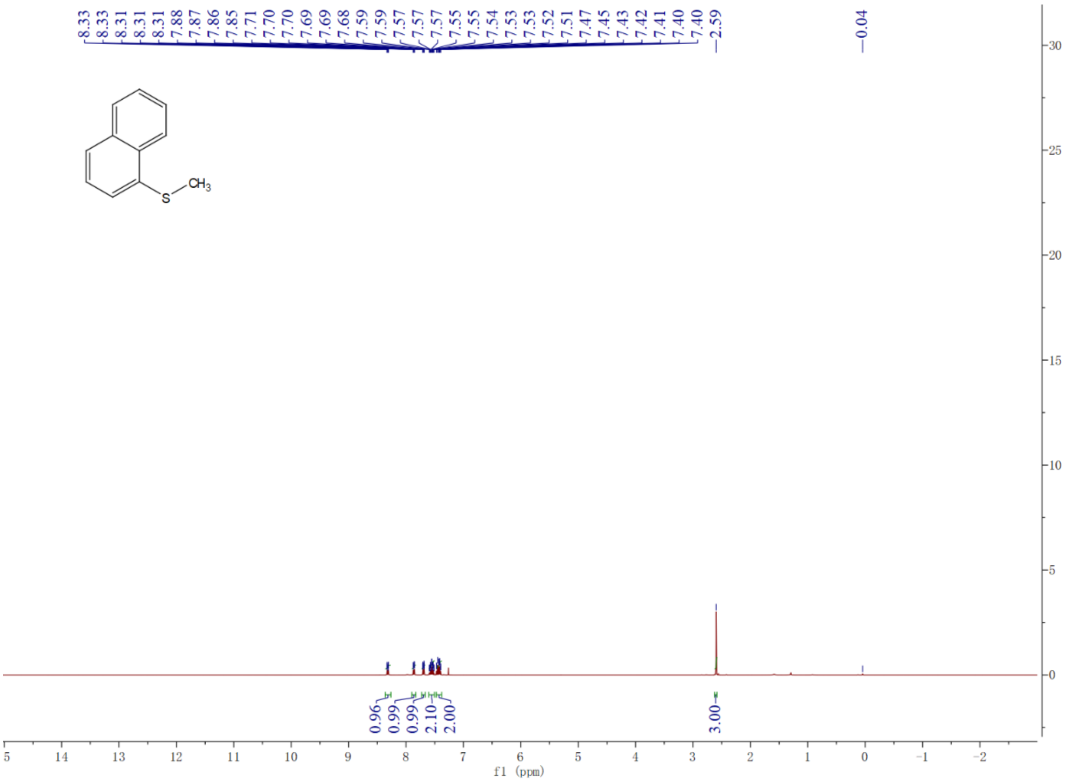

3j ^{13}C NMR

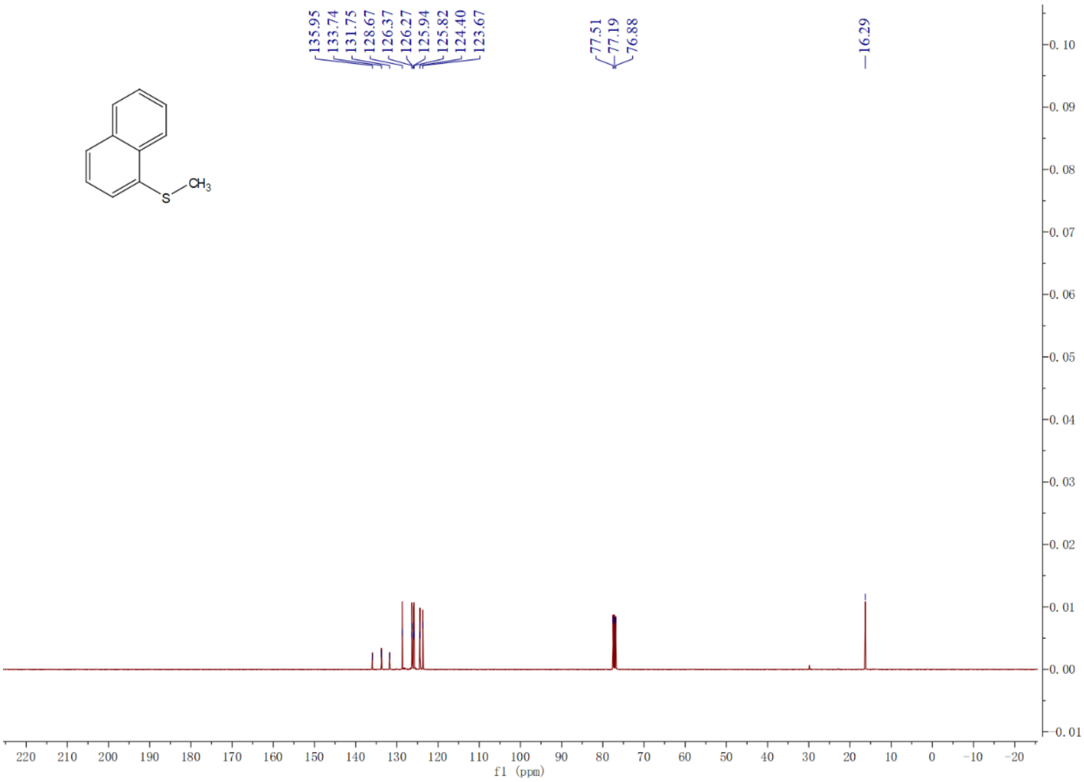

3k ^{1}H NMR

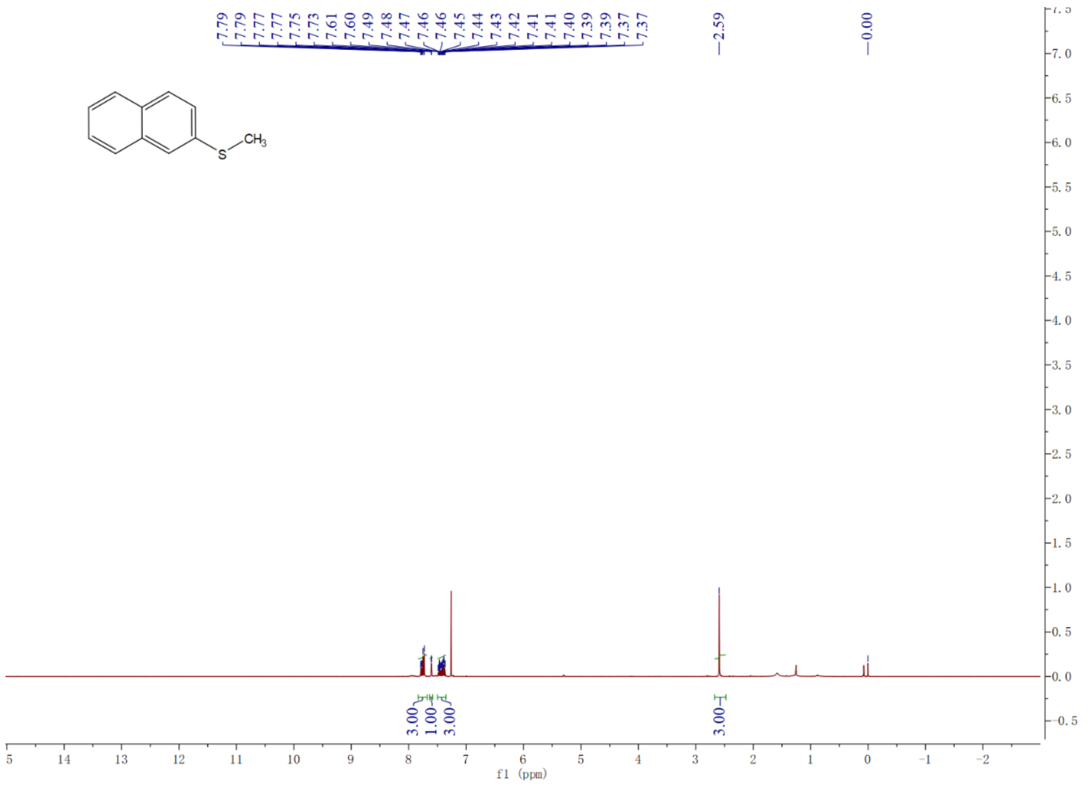

3k ^{13}C NMR

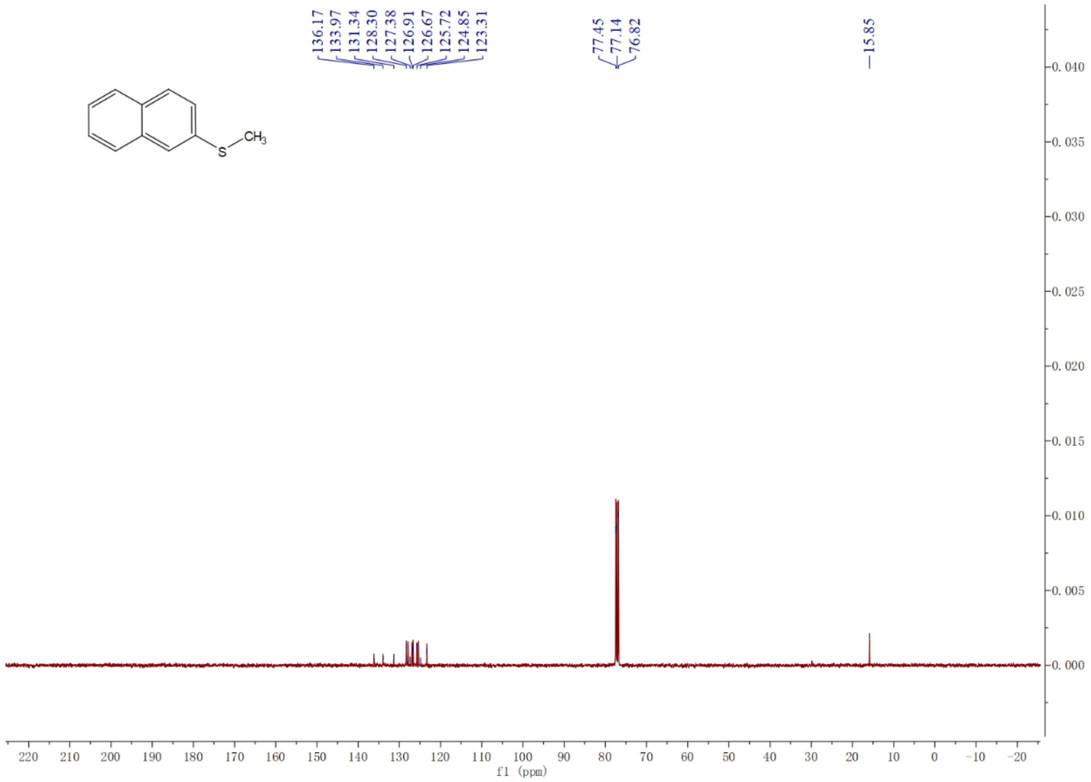

3l ^{1}H NMR

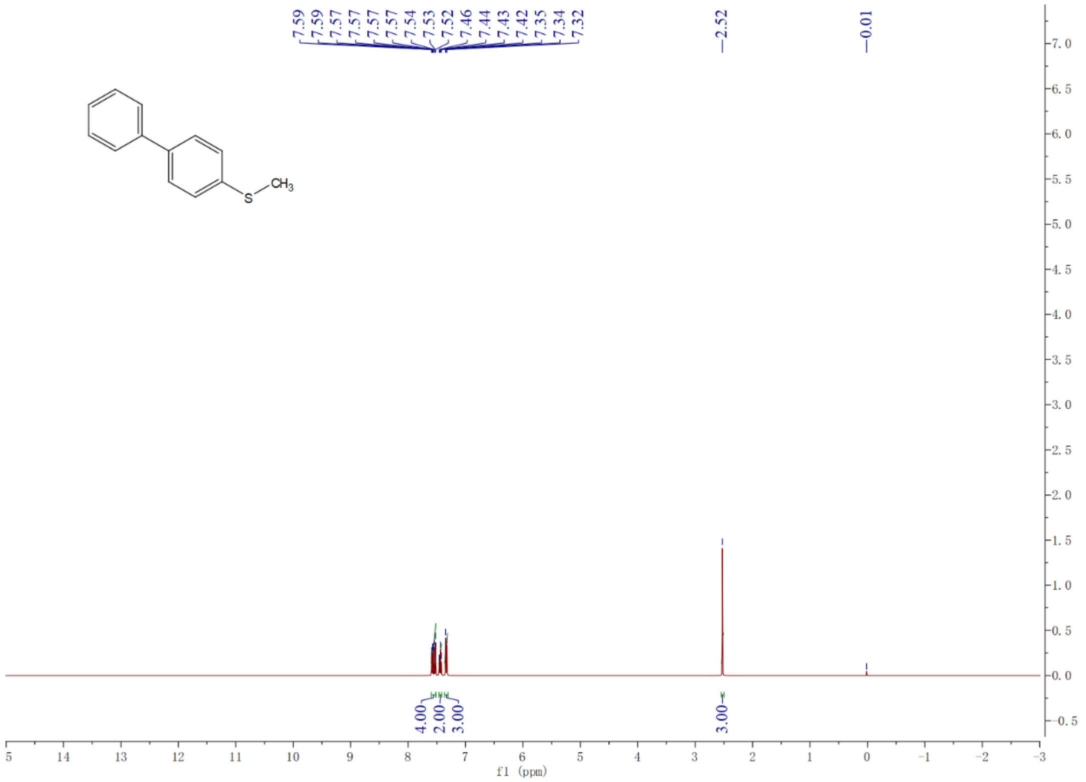

3l ^{13}C NMR

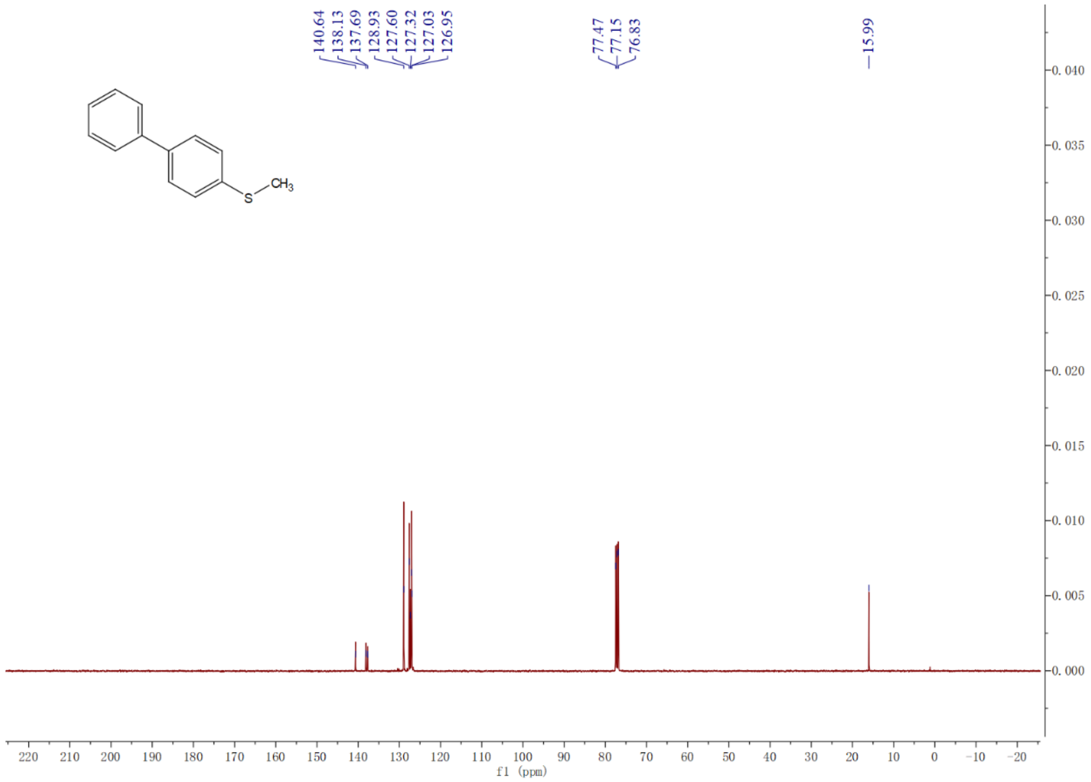

3m ^{1}H NMR

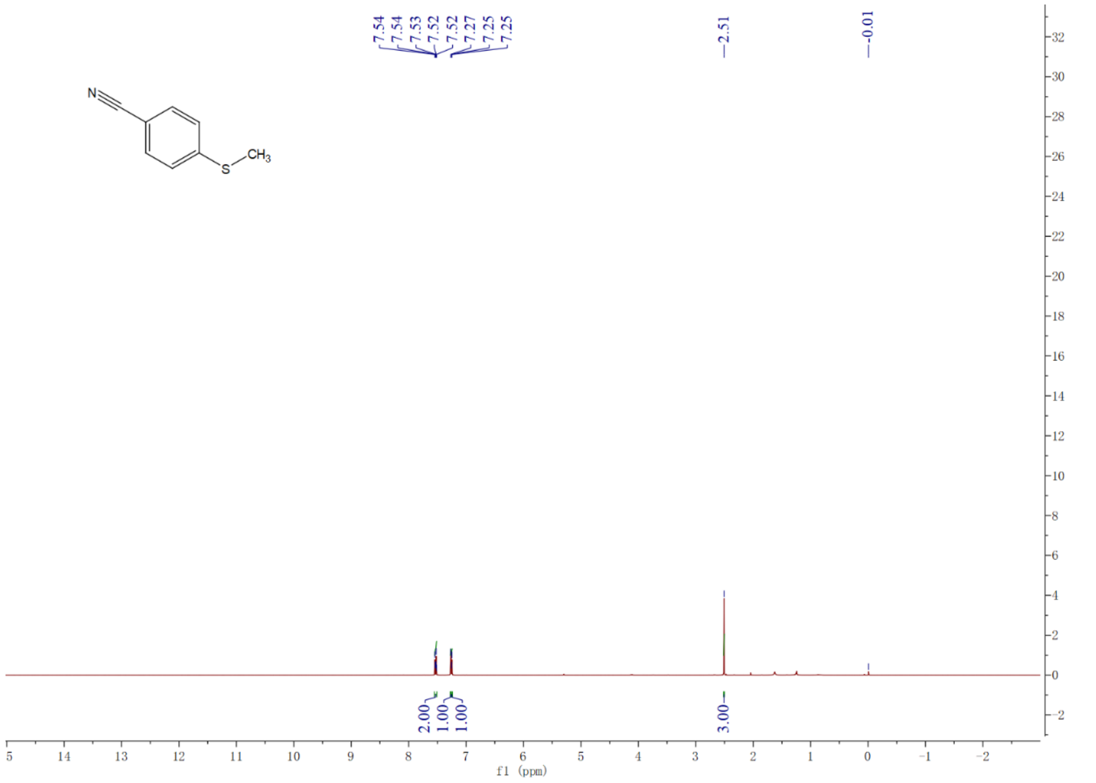

3m ^{13}C NMR

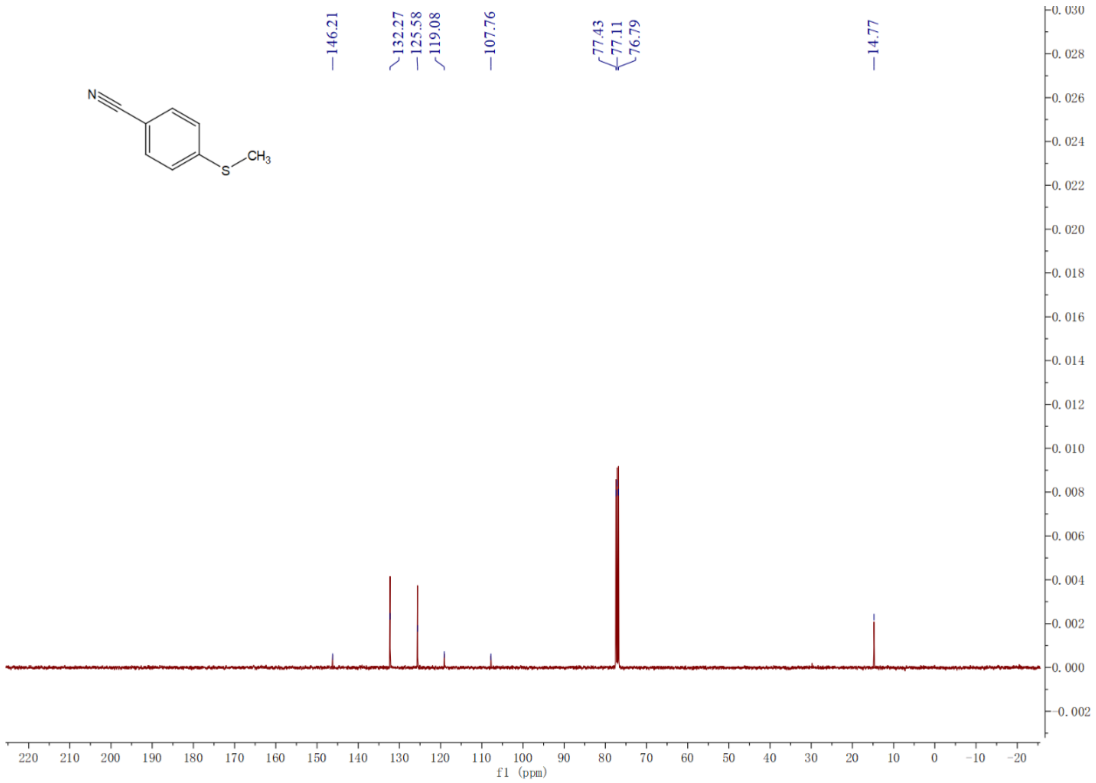

3n ^{1}H NMR

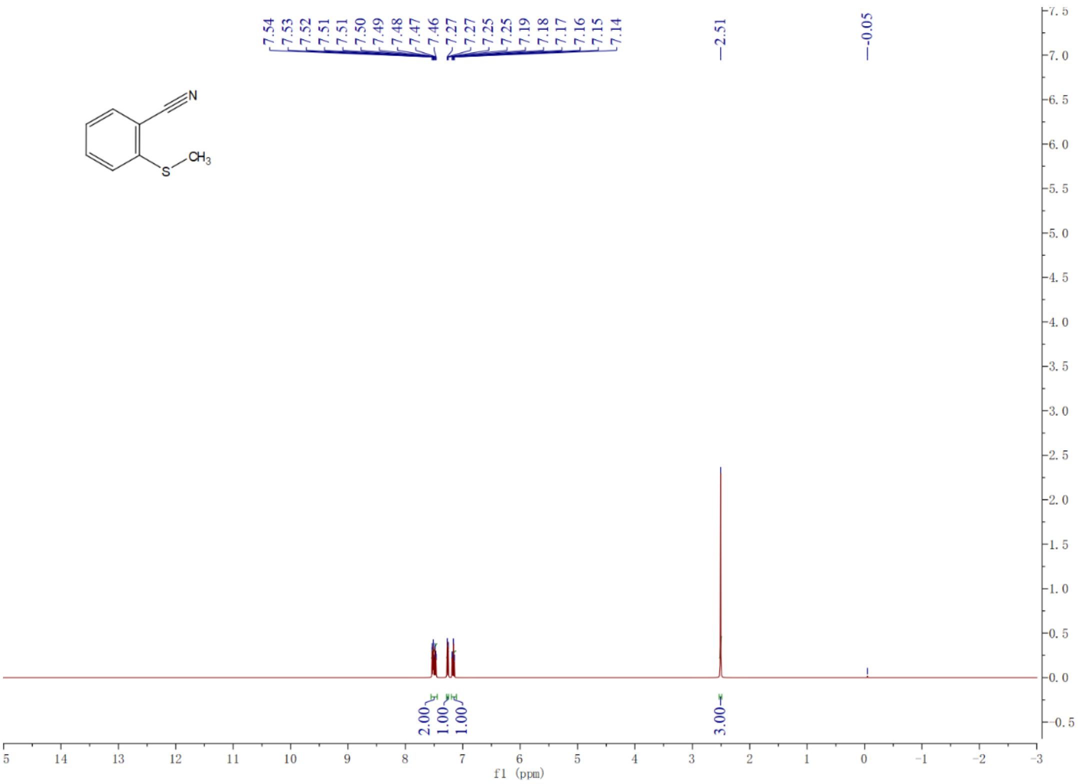

3n ^{13}C NMR

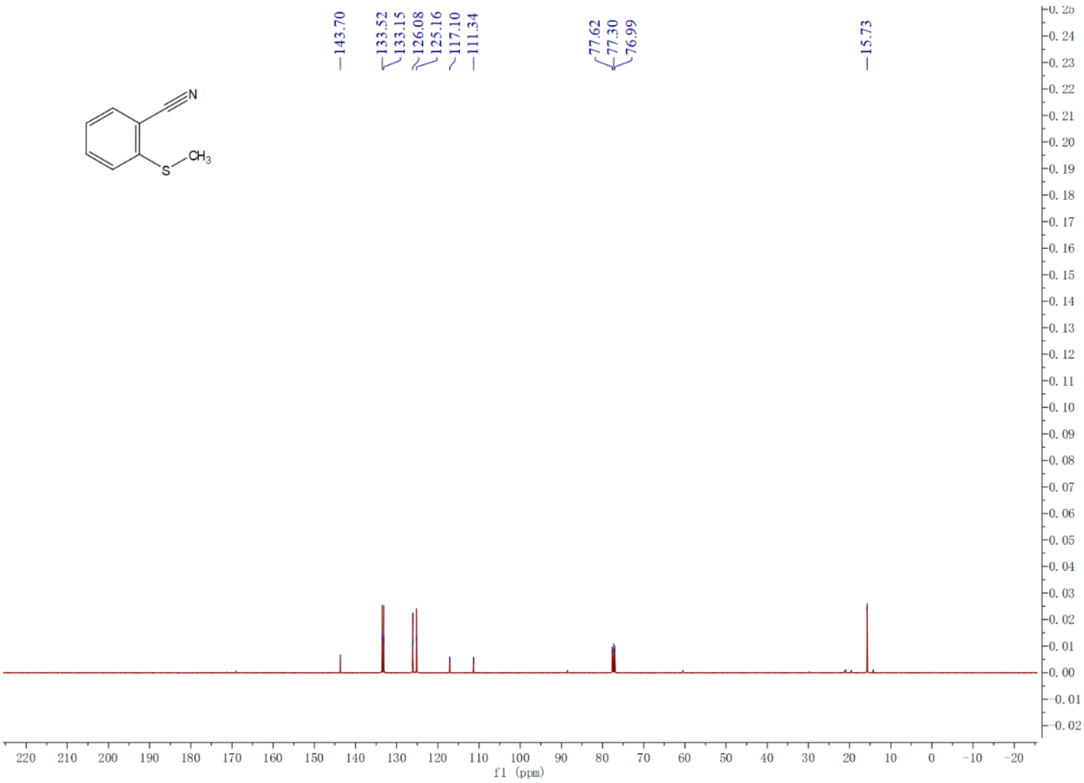

3o ^{1}H NMR

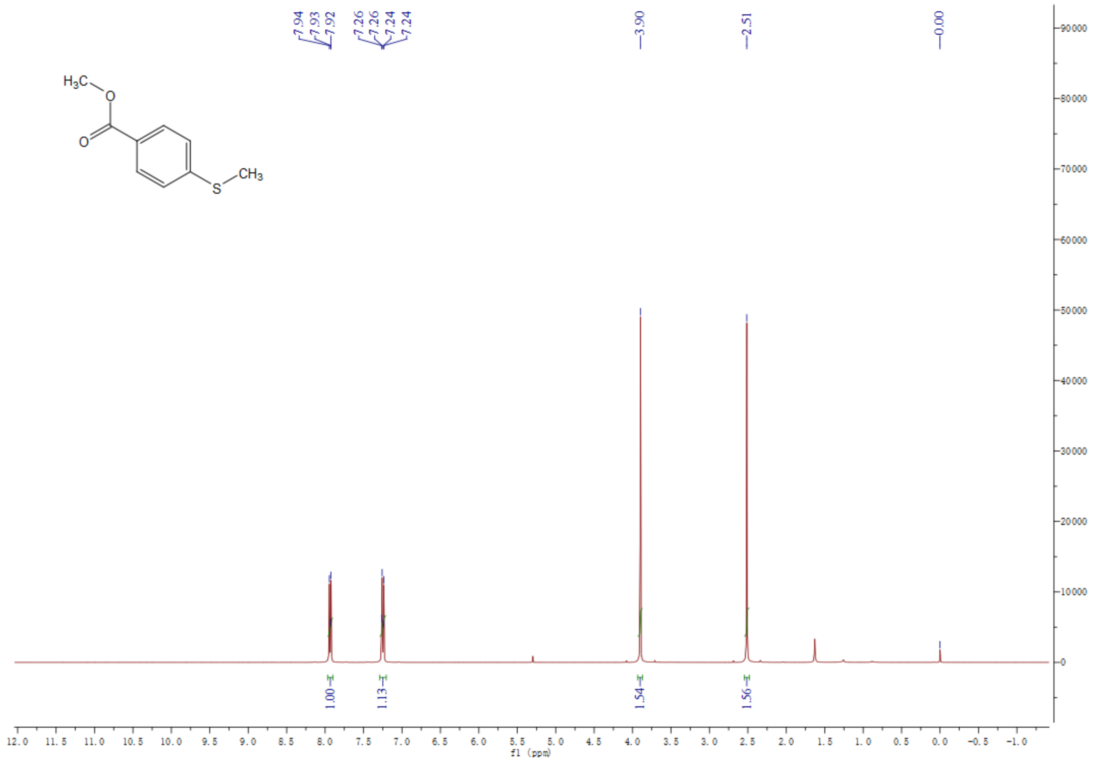

3o ^{13}C NMR

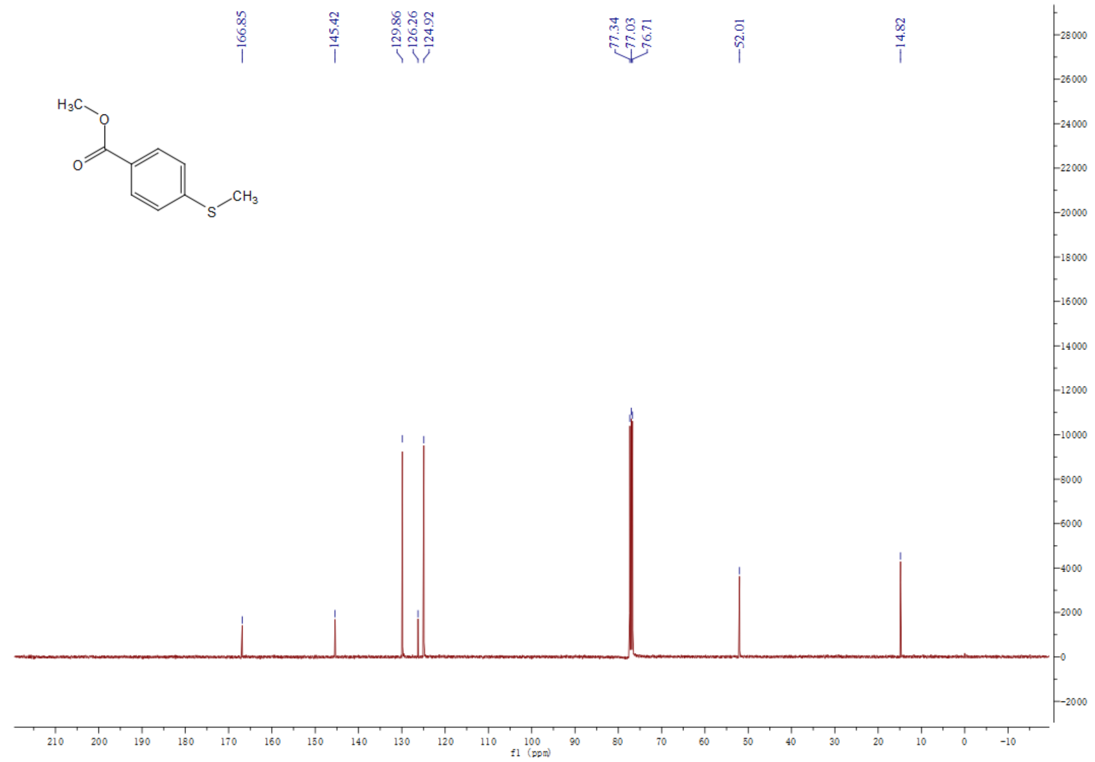

3p ^{1}H NMR

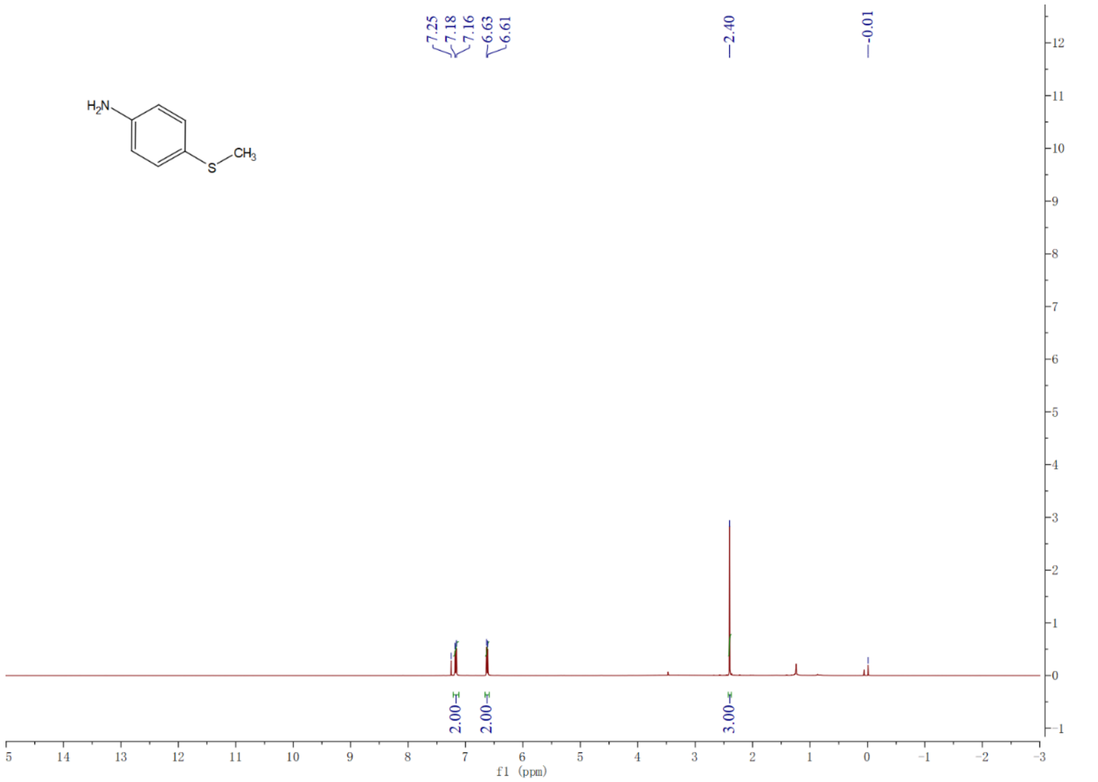

3p ^{13}C NMR

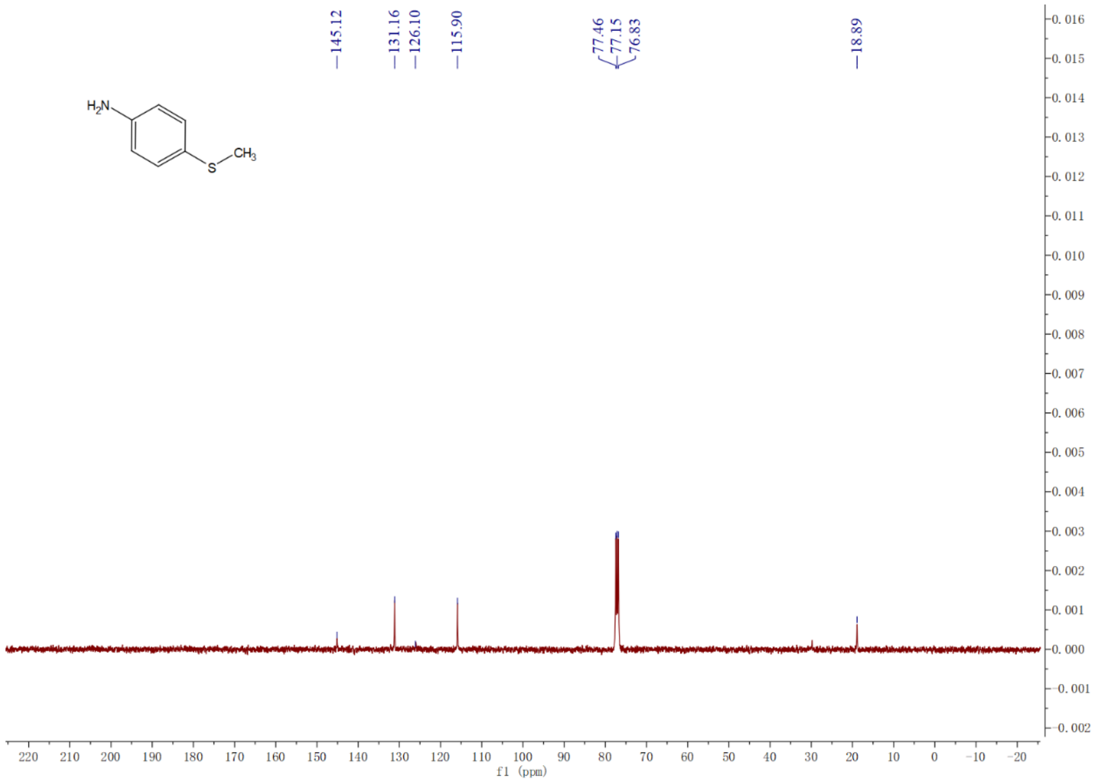

3q ^{1}H NMR

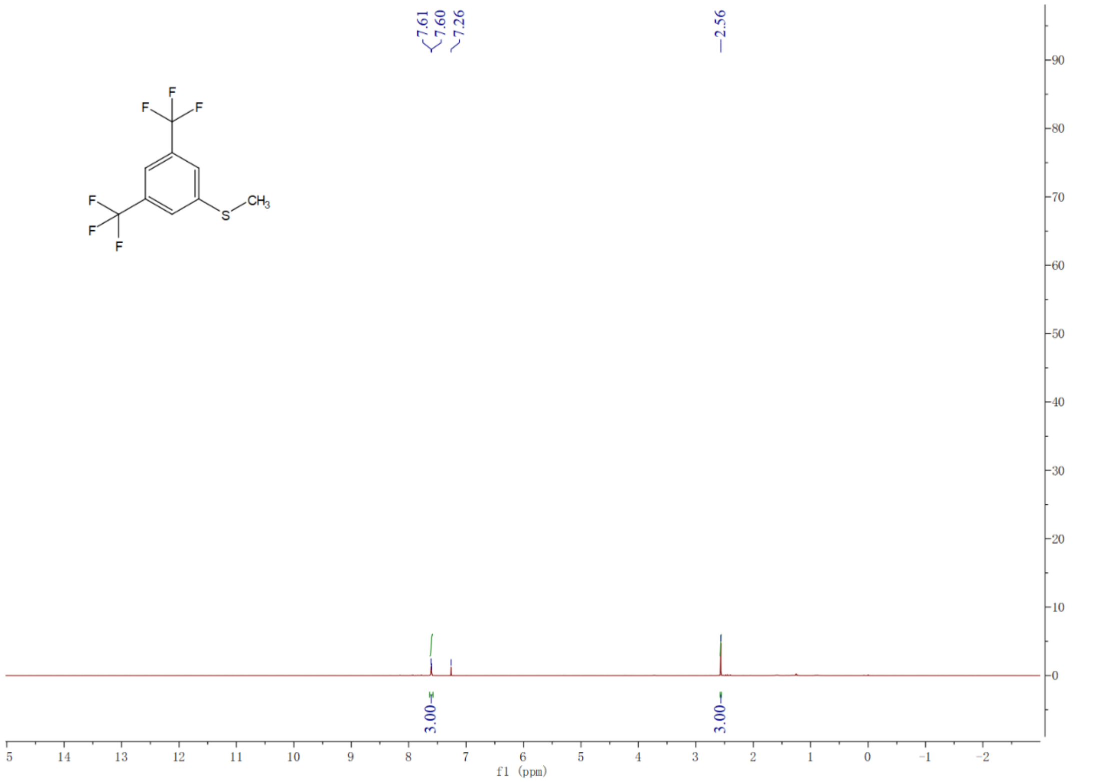

3q ^{13}C NMR

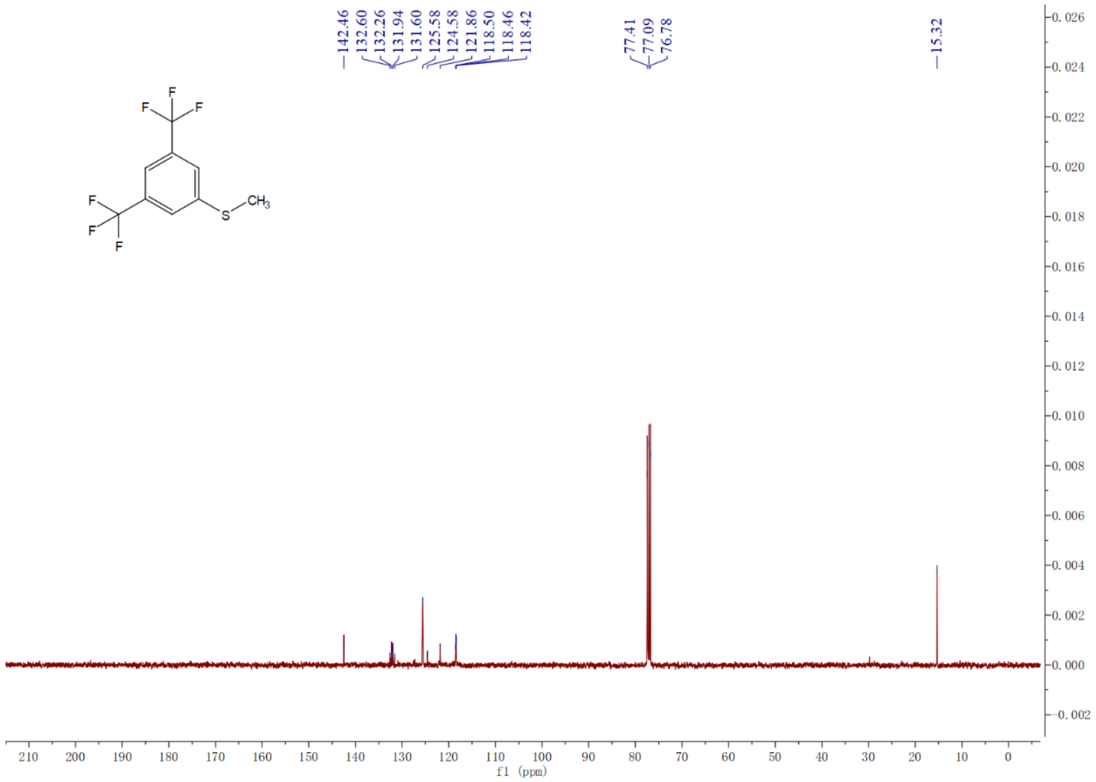

3r ^{1}H NMR

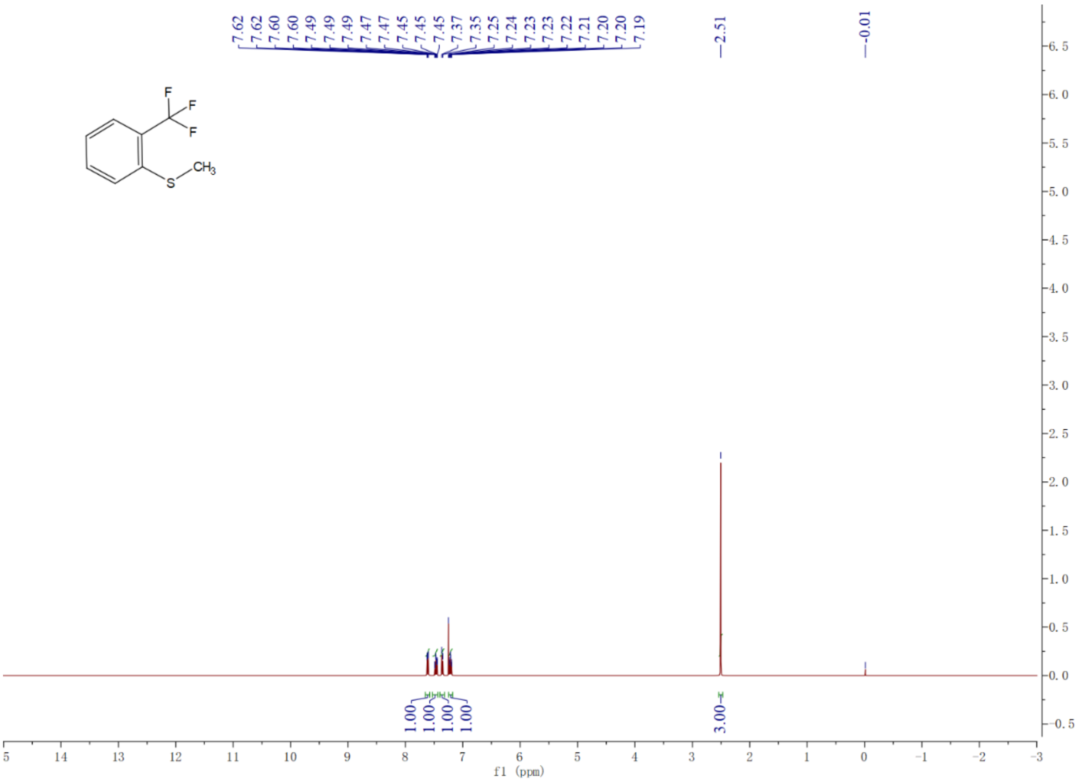

3r ^{13}C NMR

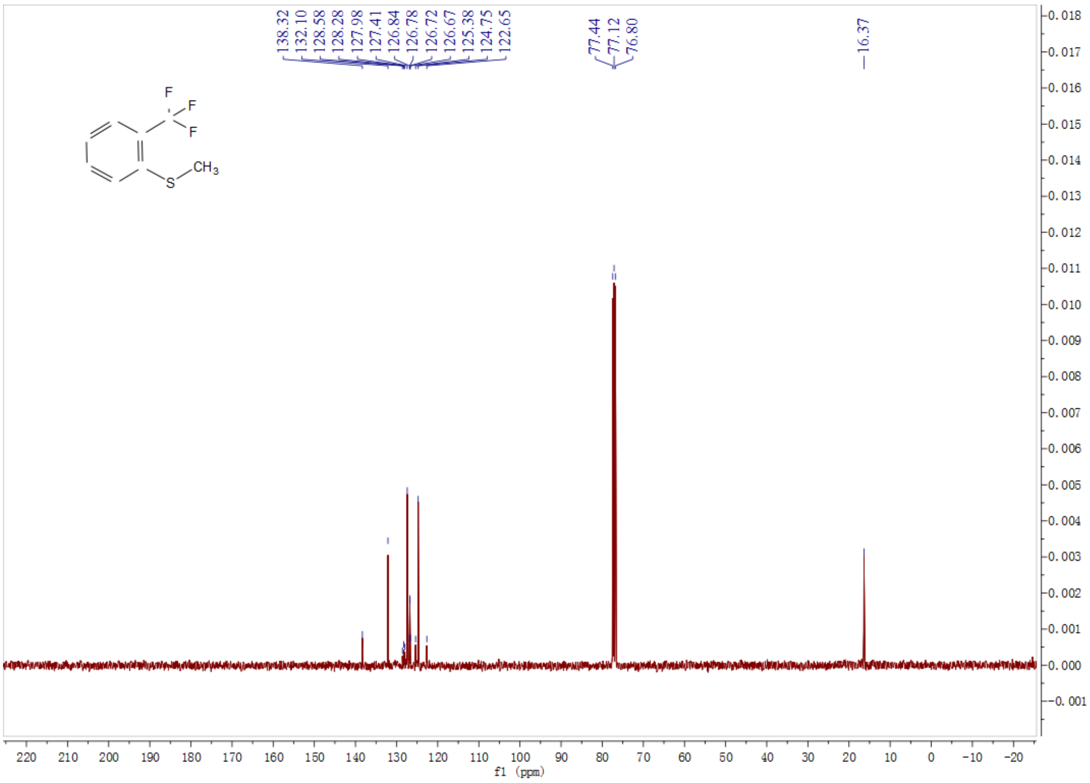

3s ^{1}H NMR

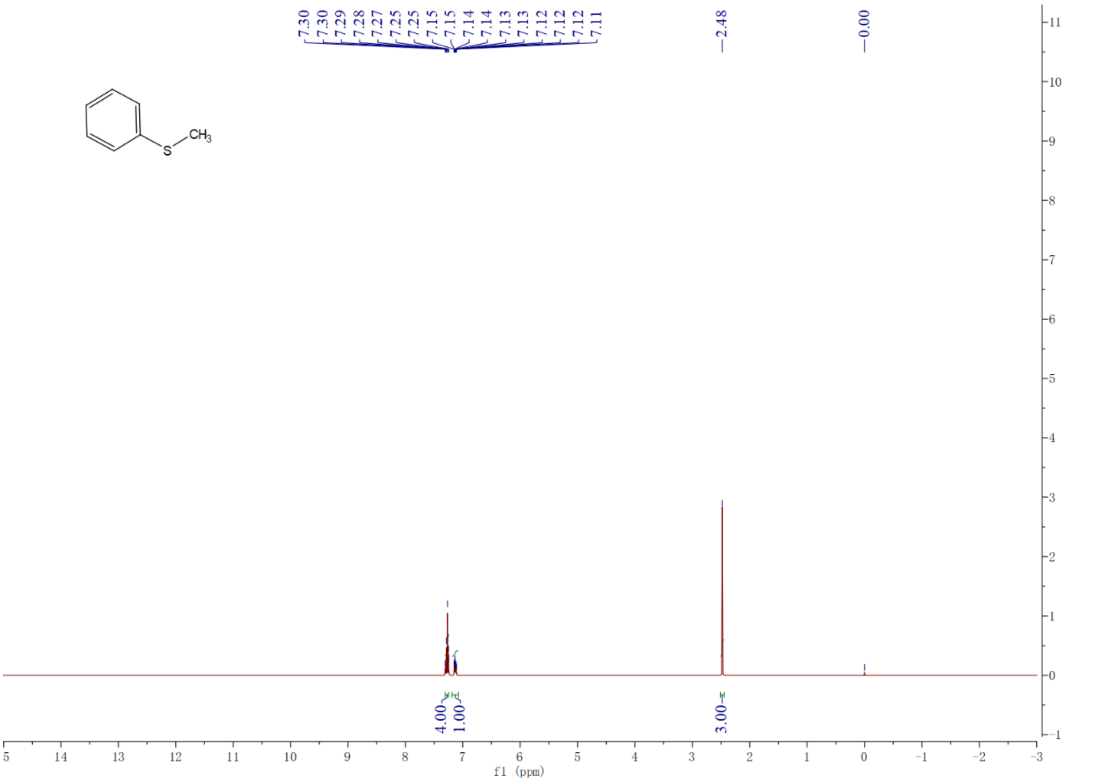

3s ^{13}C NMR

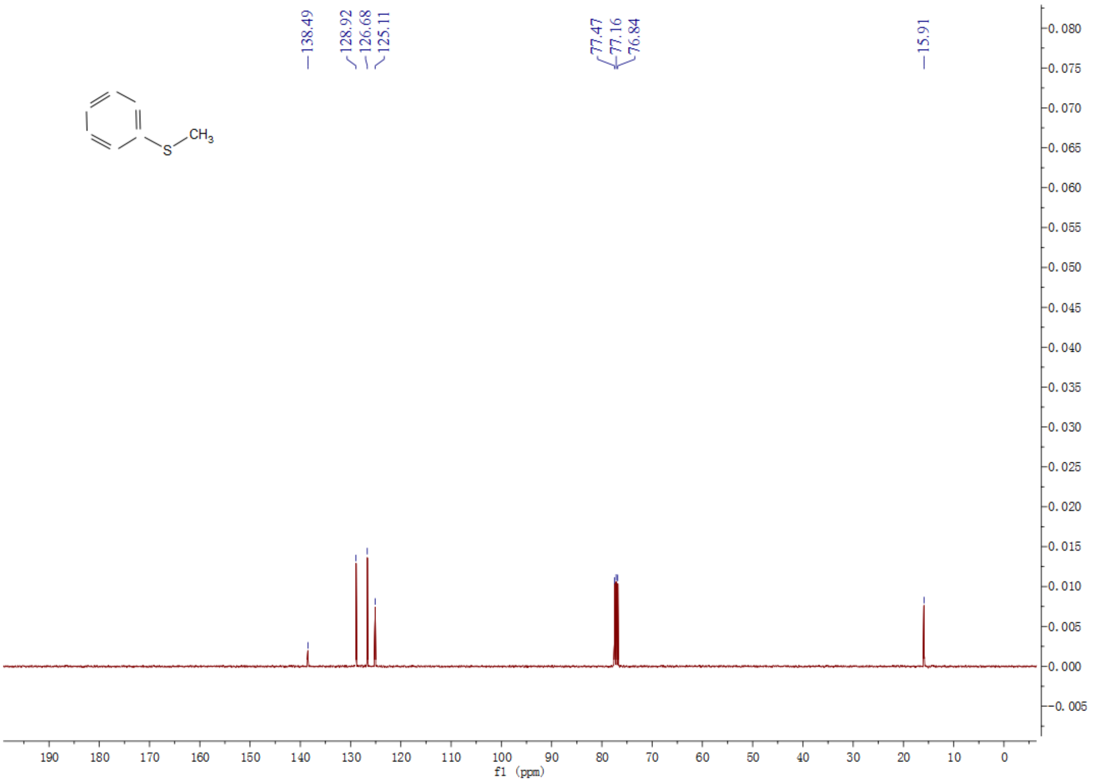

3t ^{1}H NMR

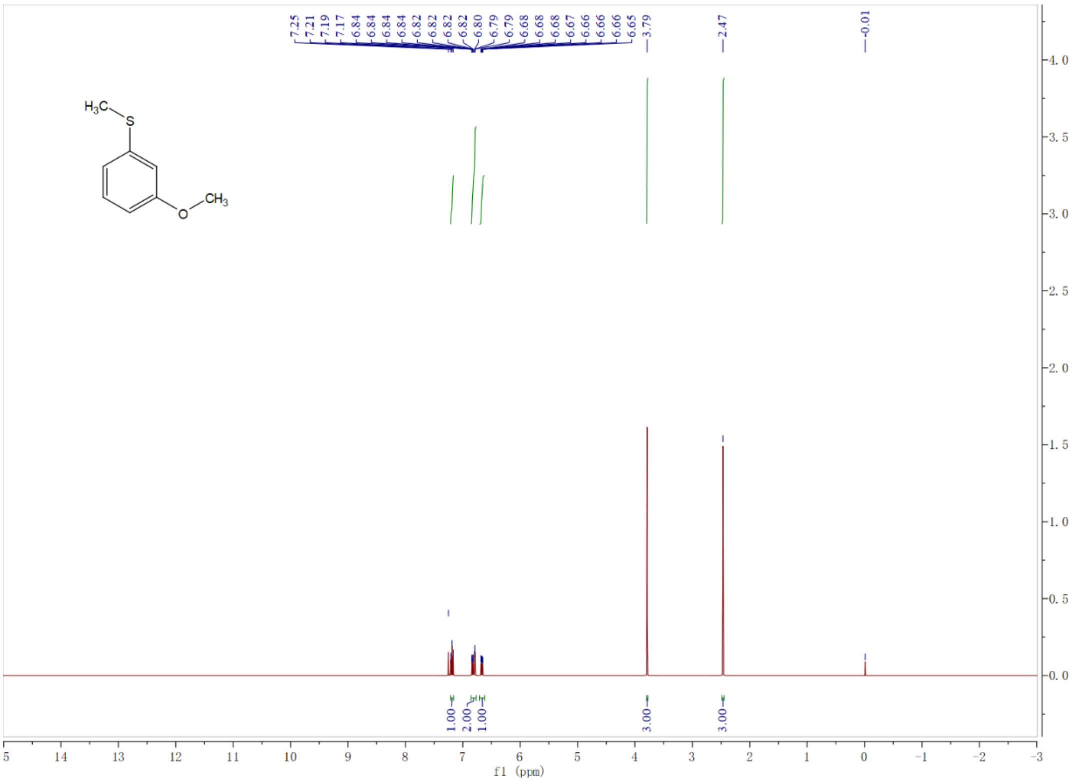

3t ^{13}C NMR

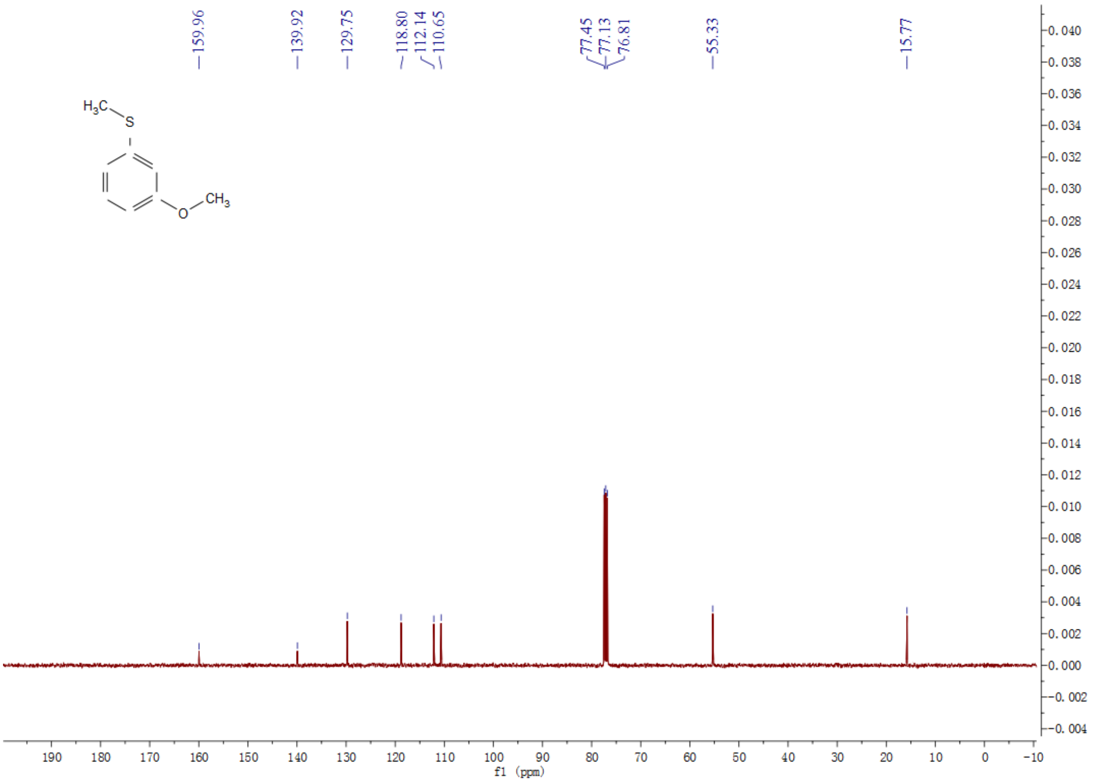

3u ^{1}H NMR

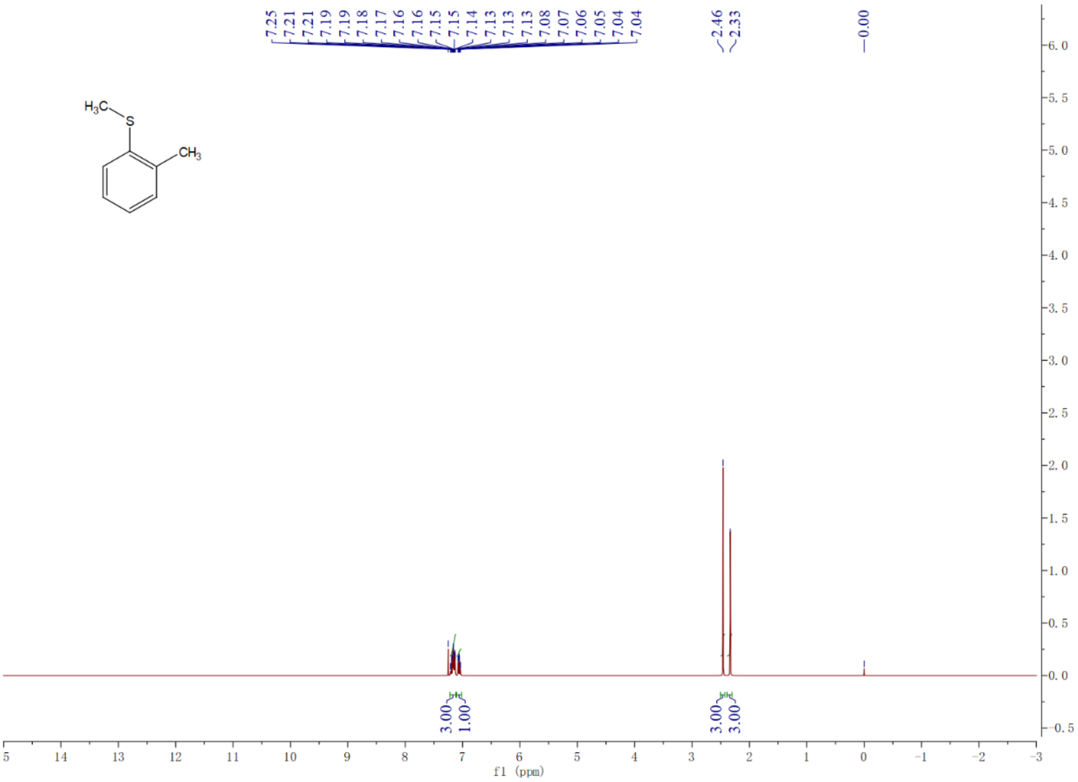

3u ^{13}C NMR

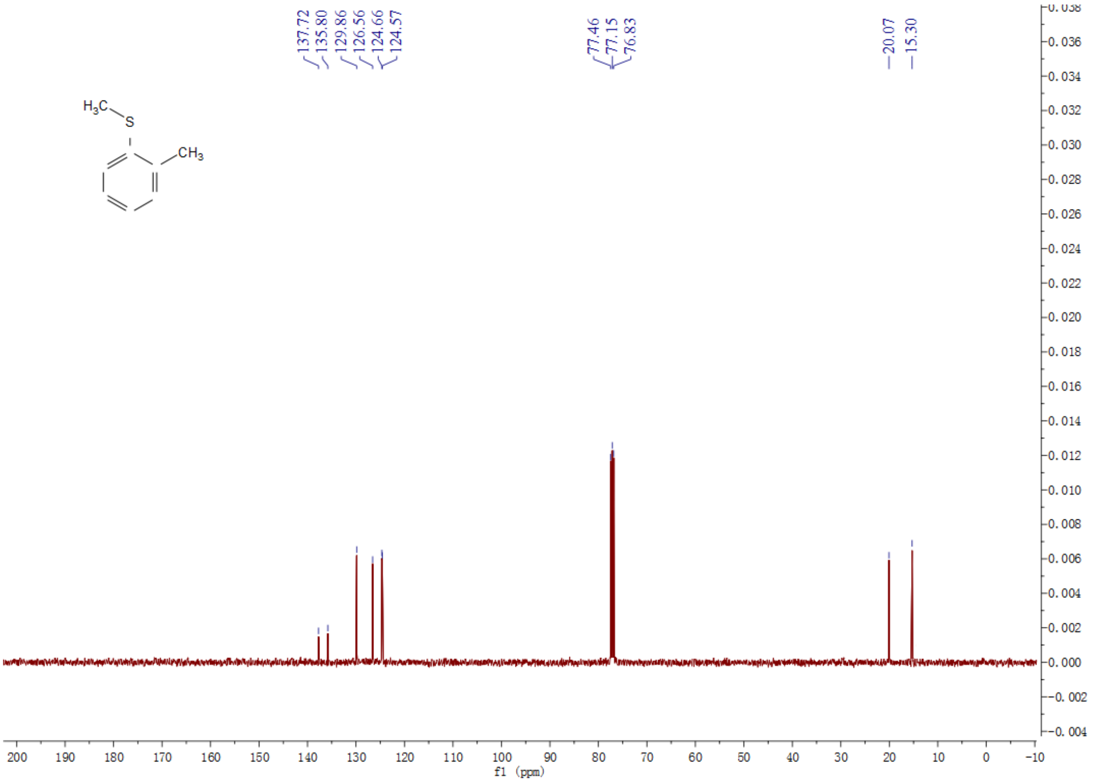

3v ^{1}H NMR

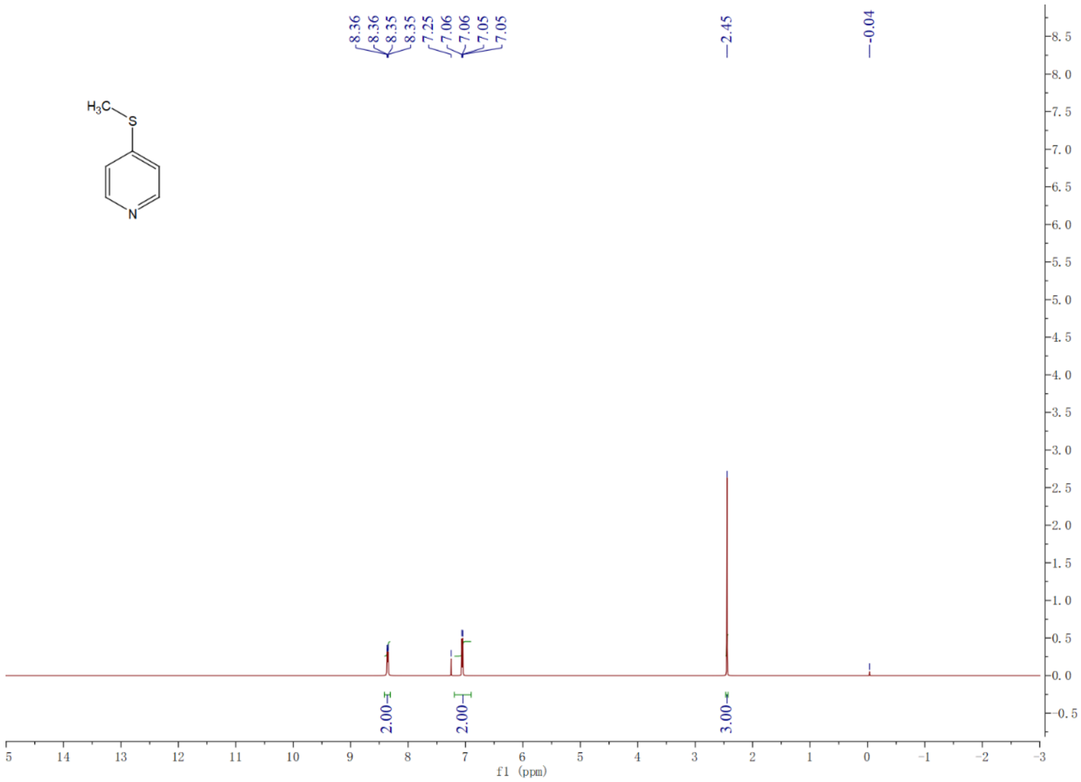

3v ^{13}C NMR

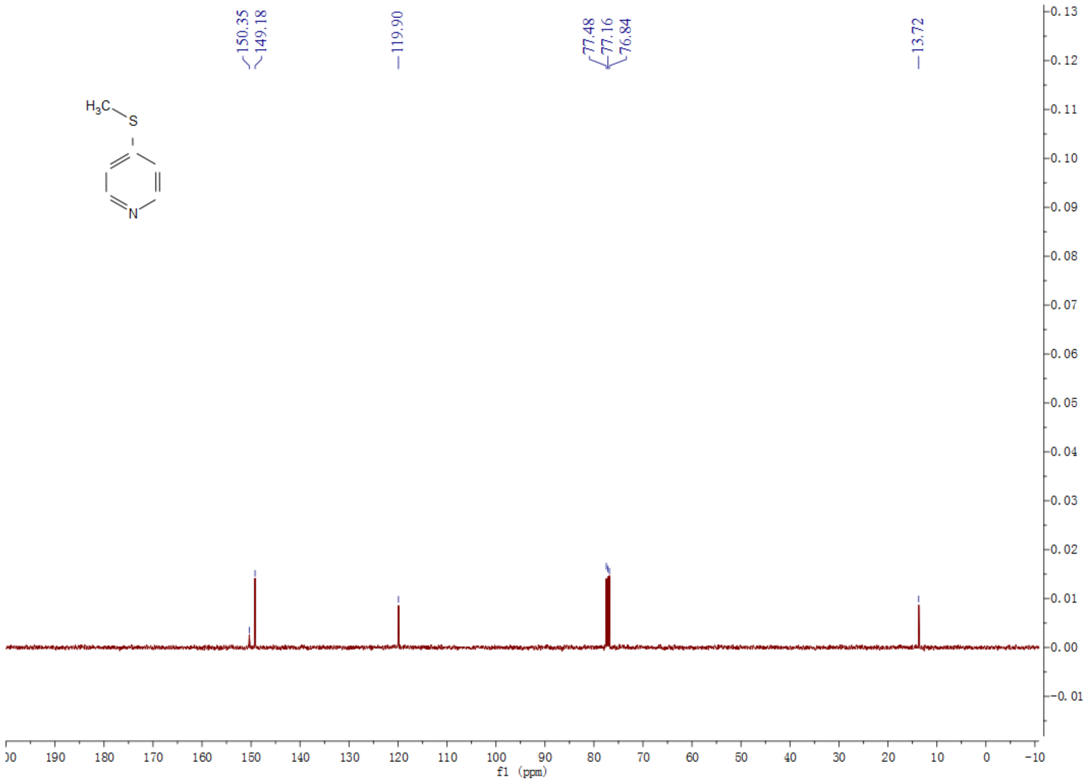

3w ^{1}H NMR

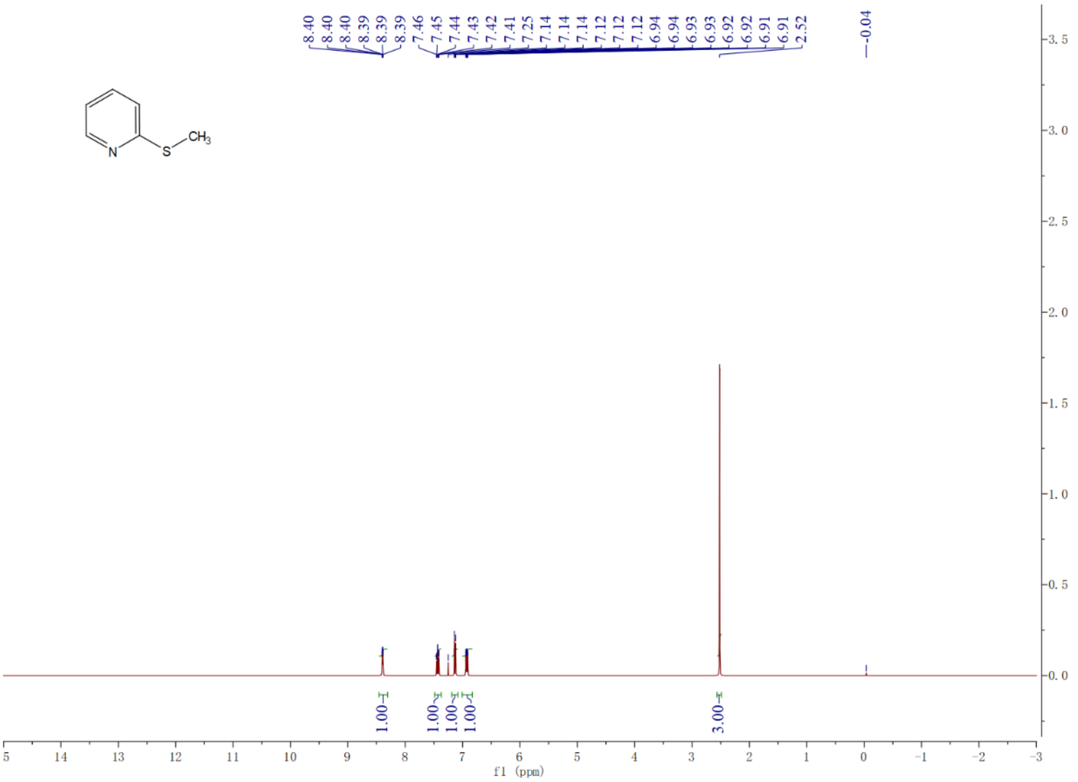

3w ^{13}C NMR

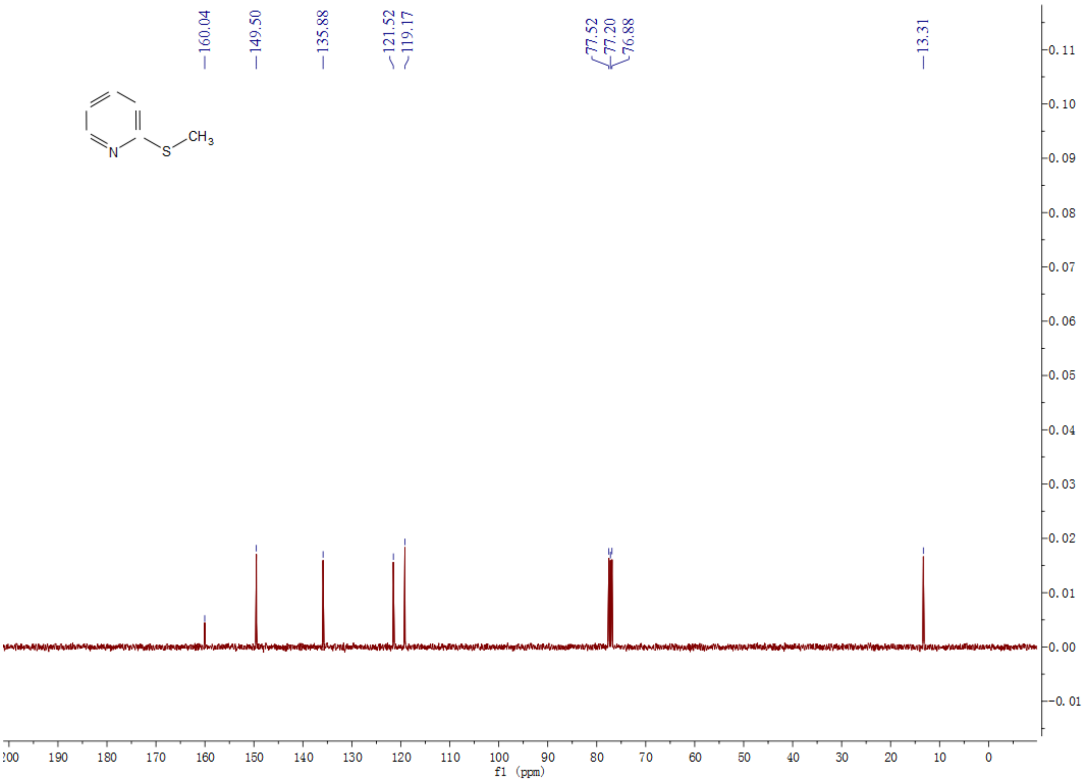

3x ^{1}H NMR

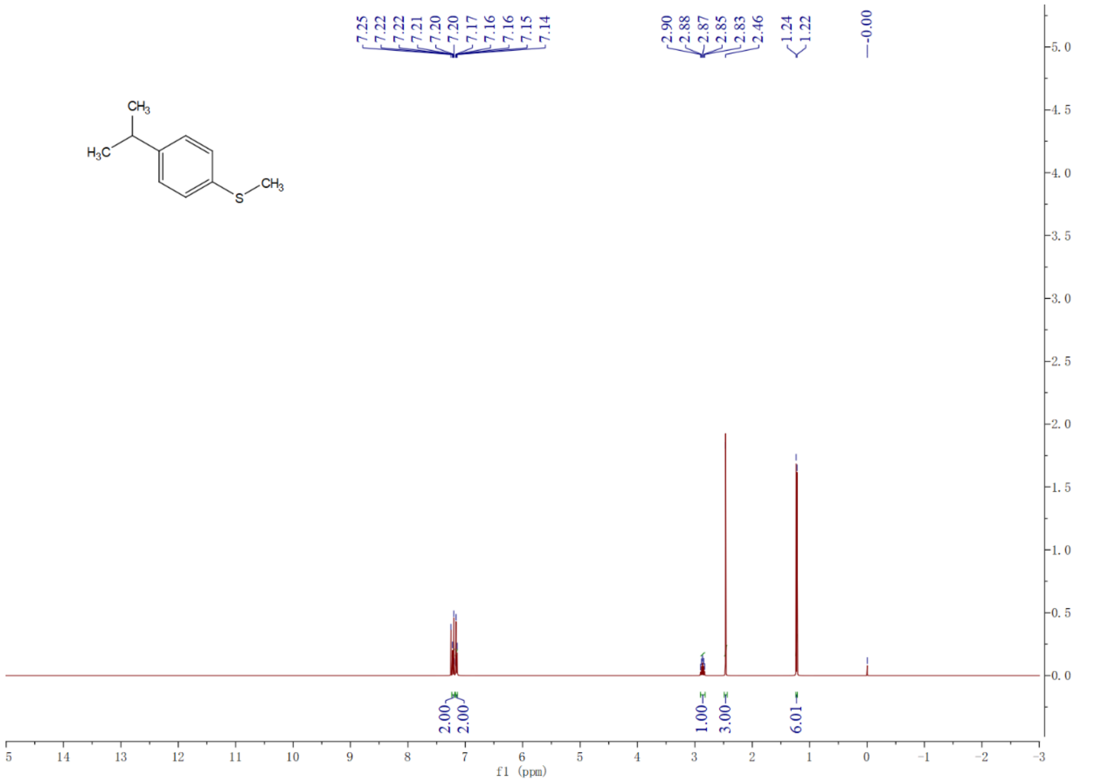

3x ^{13}C NMR

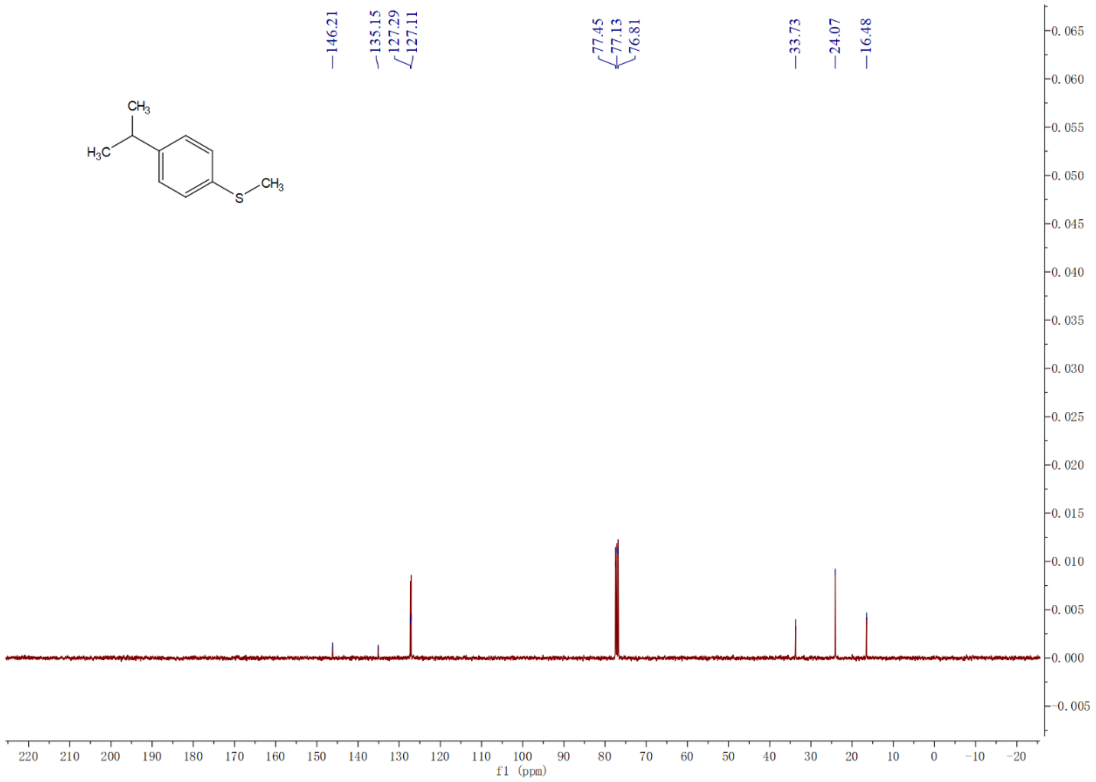

3y ^{1}H NMR

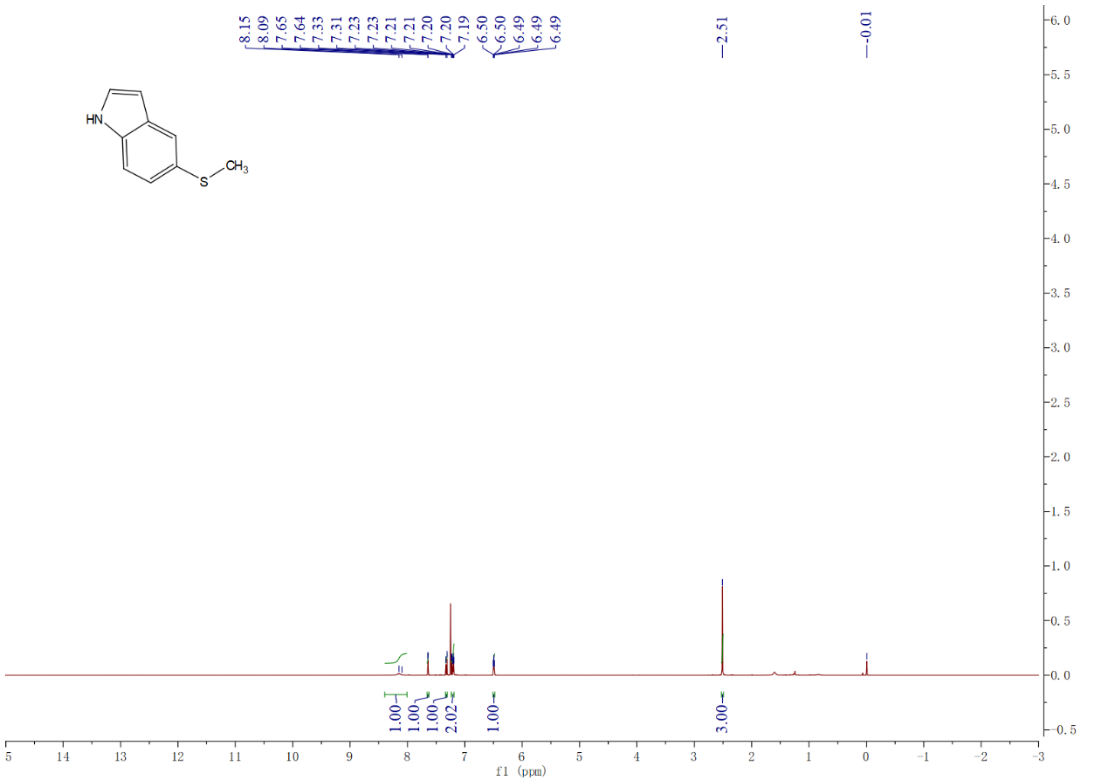

3y ^{13}C NMR

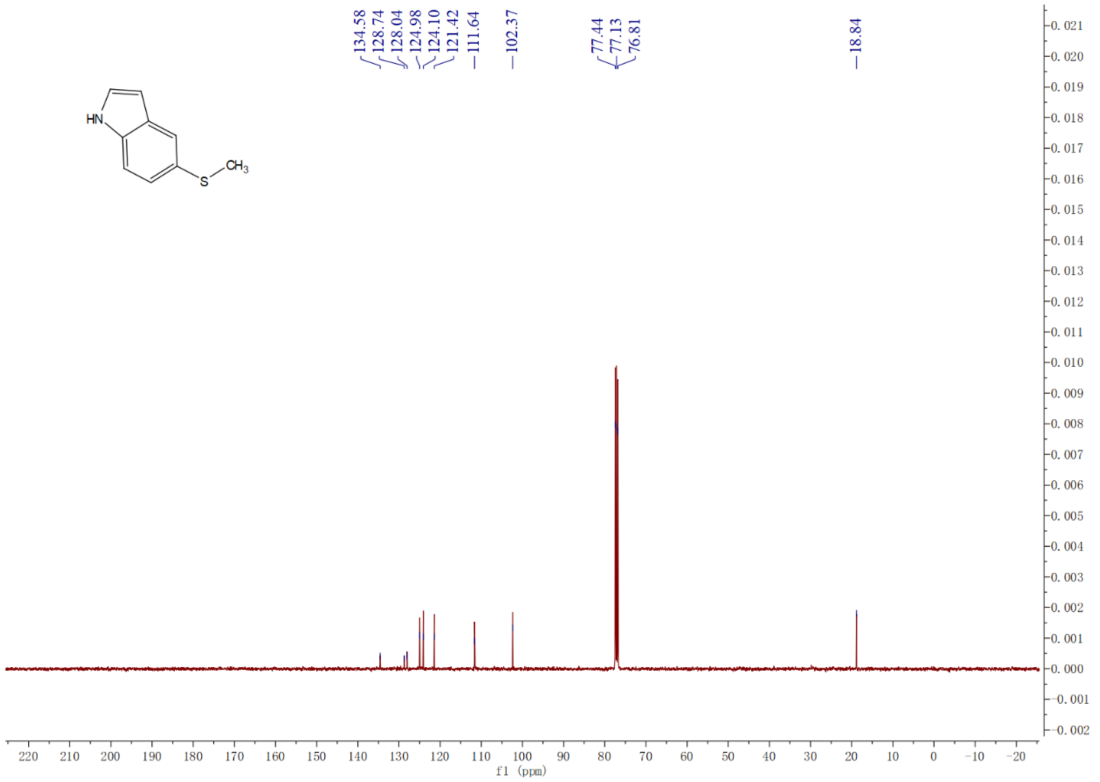

3z ^{1}H NMR

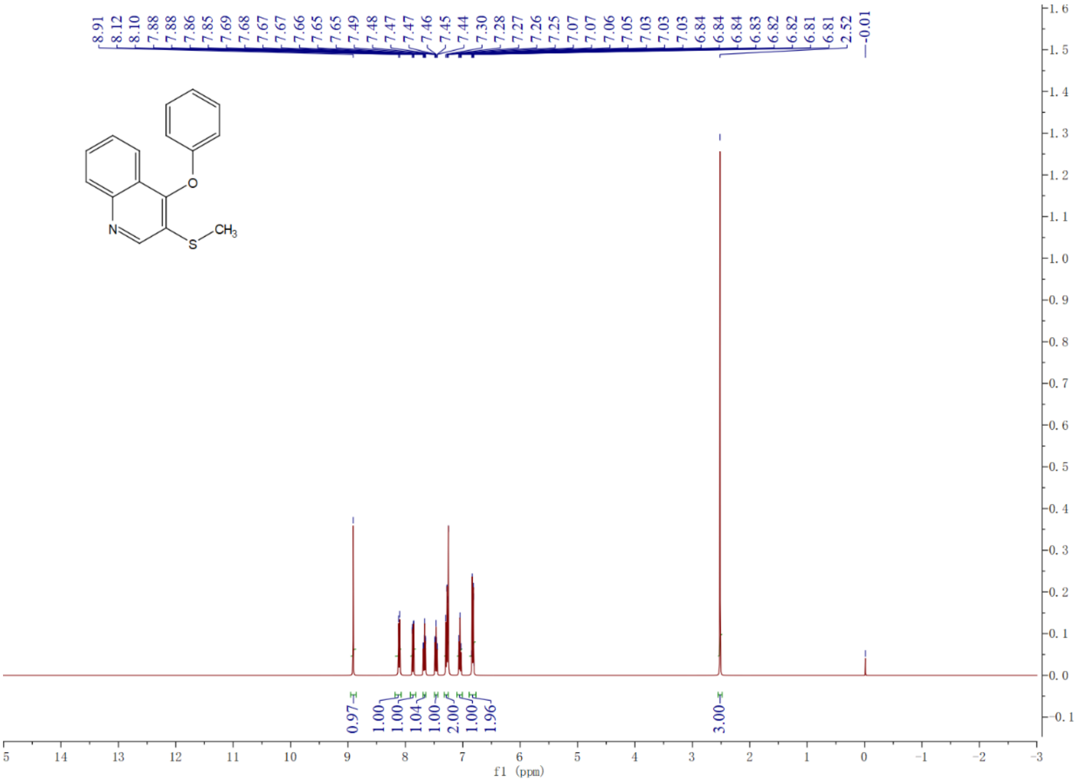

3z ^{13}C NMR

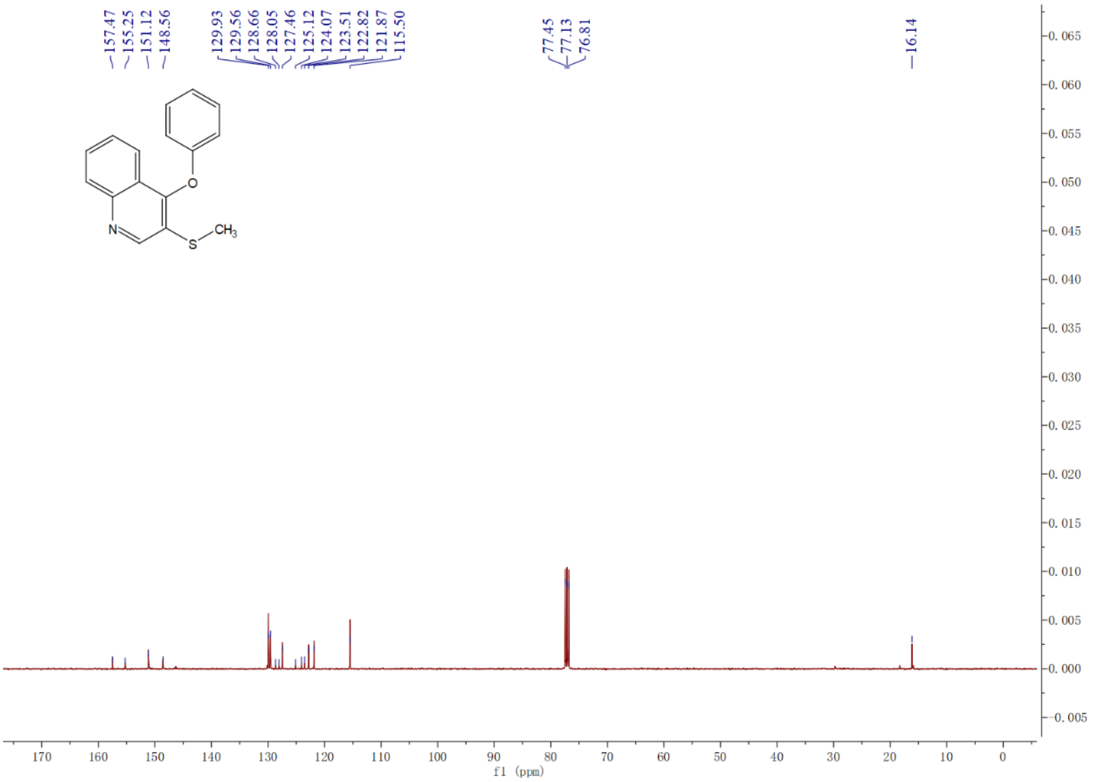

3ac ^{1}H NMR

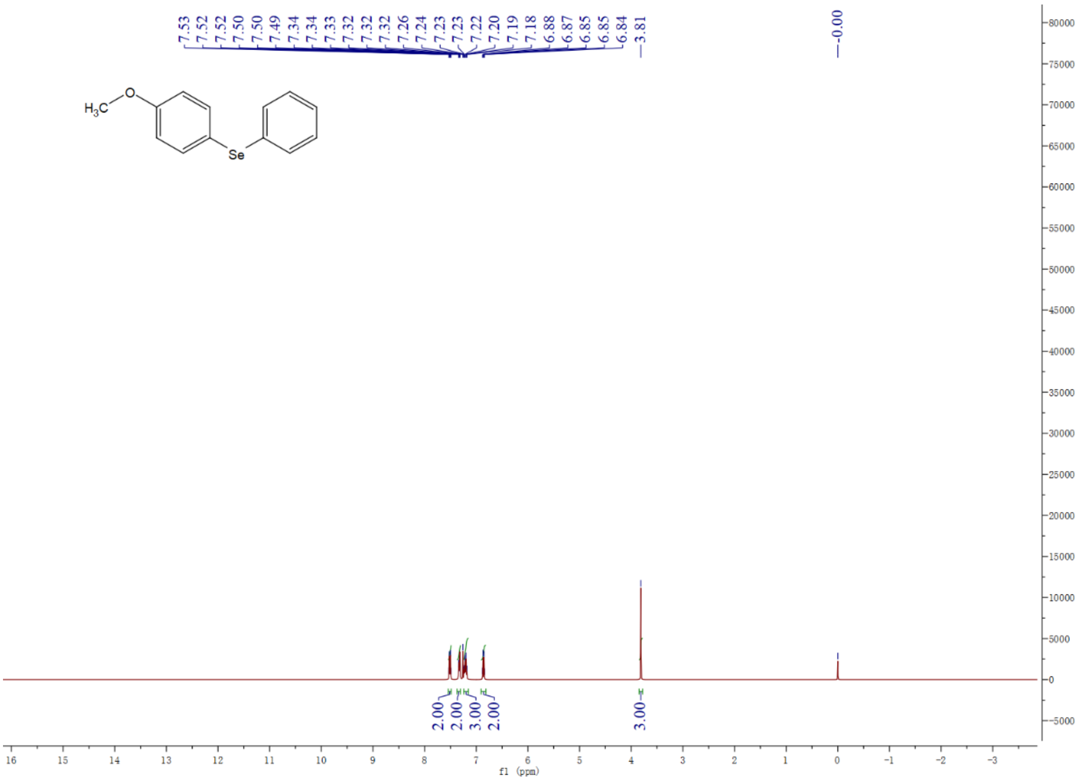

3ac ^{13}C NMR

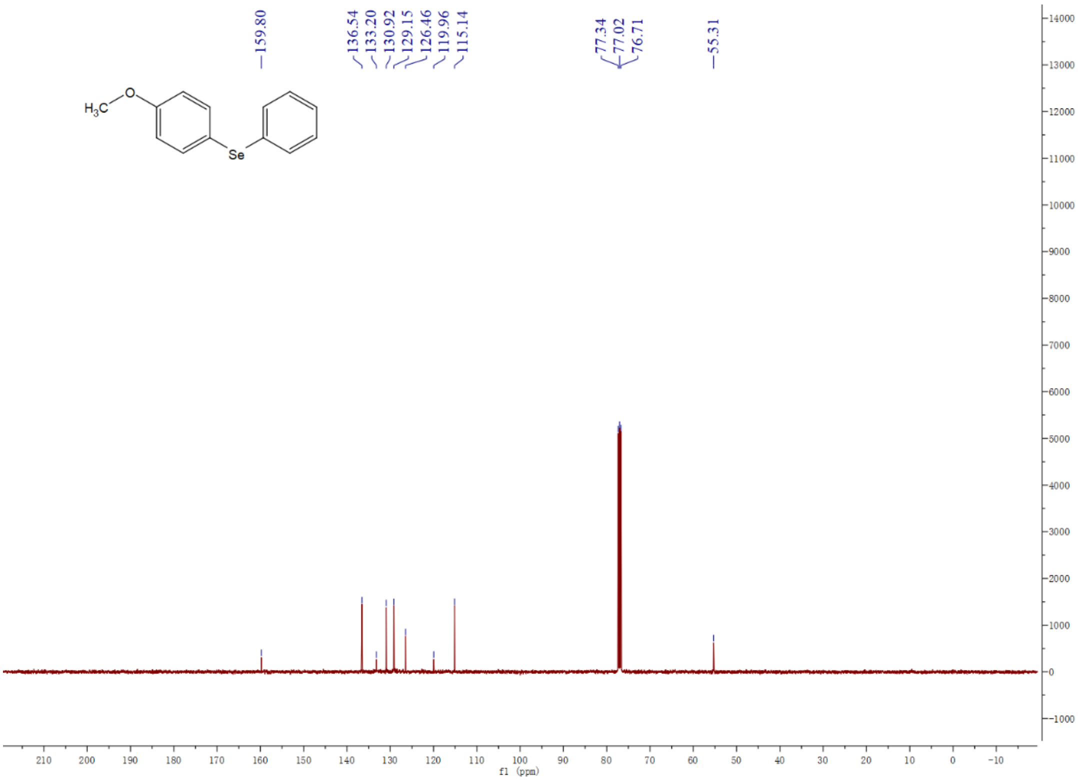

3ad ^{1}H NMR

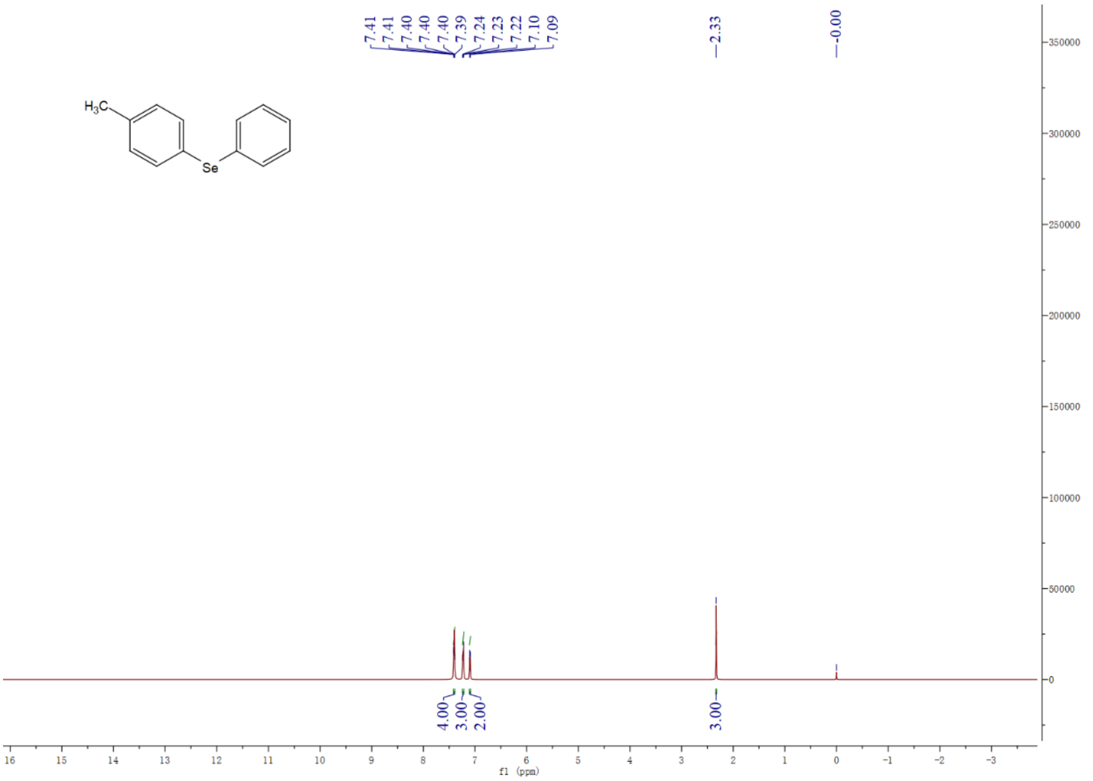

3ad ^{13}C NMR

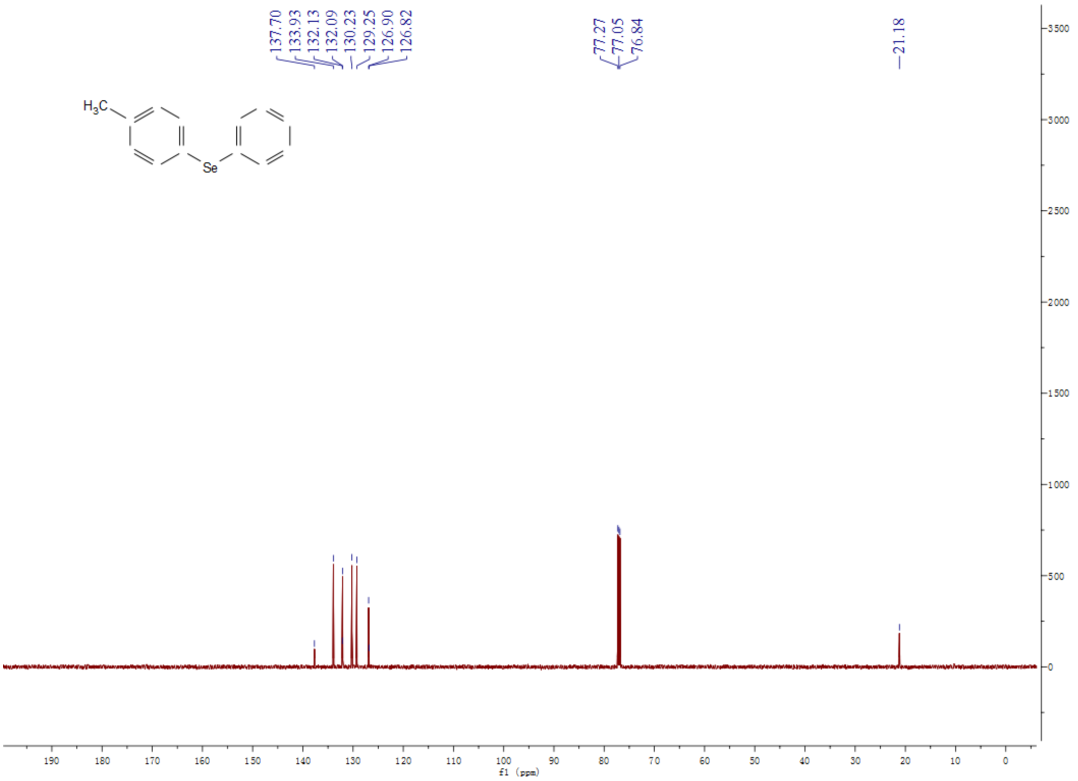

3ae ^{1}H NMR

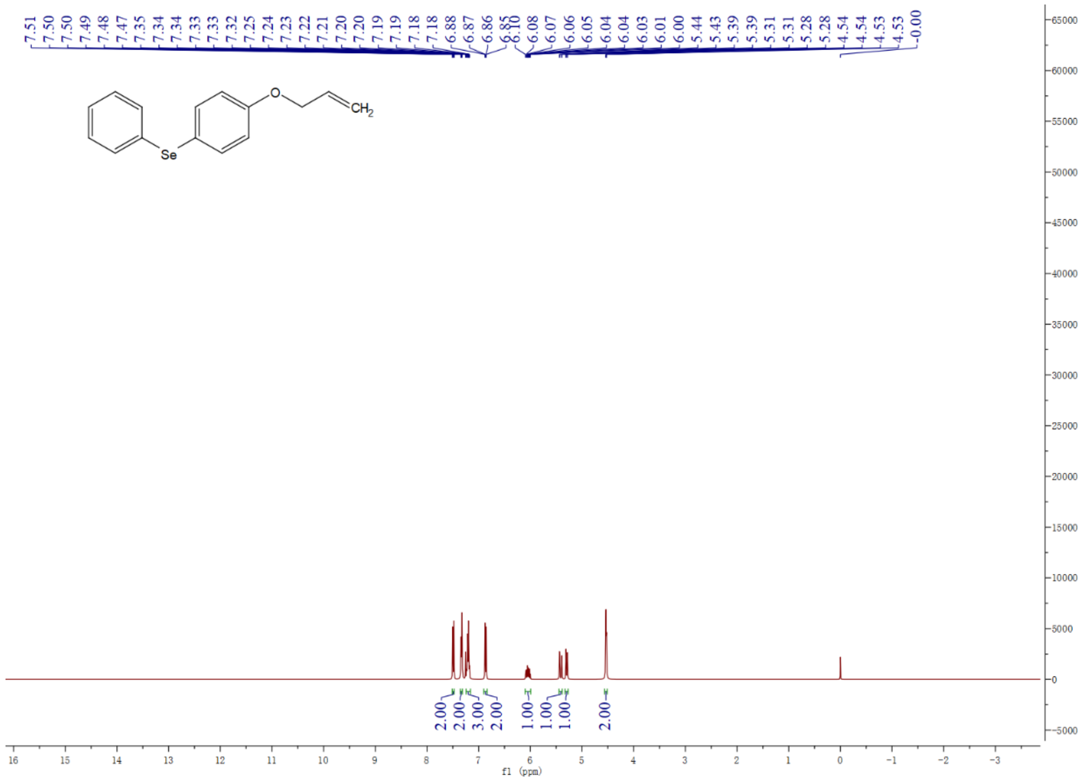

3ae ^{13}C NMR

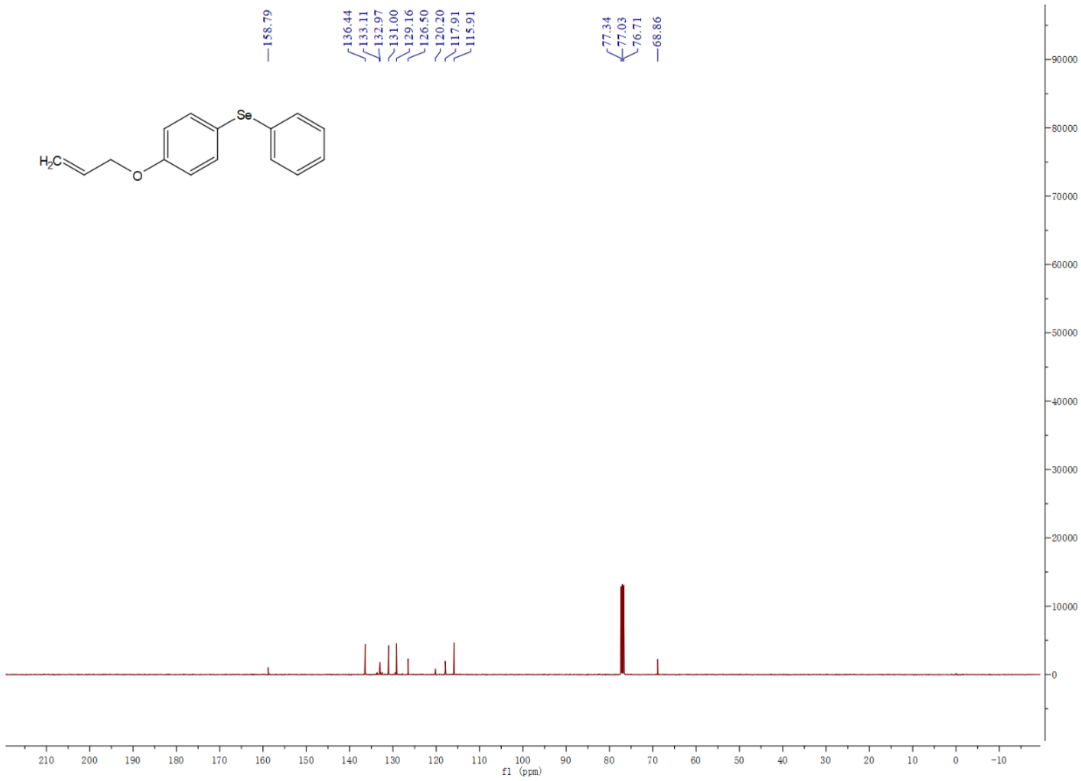

3af ^{1}H NMR

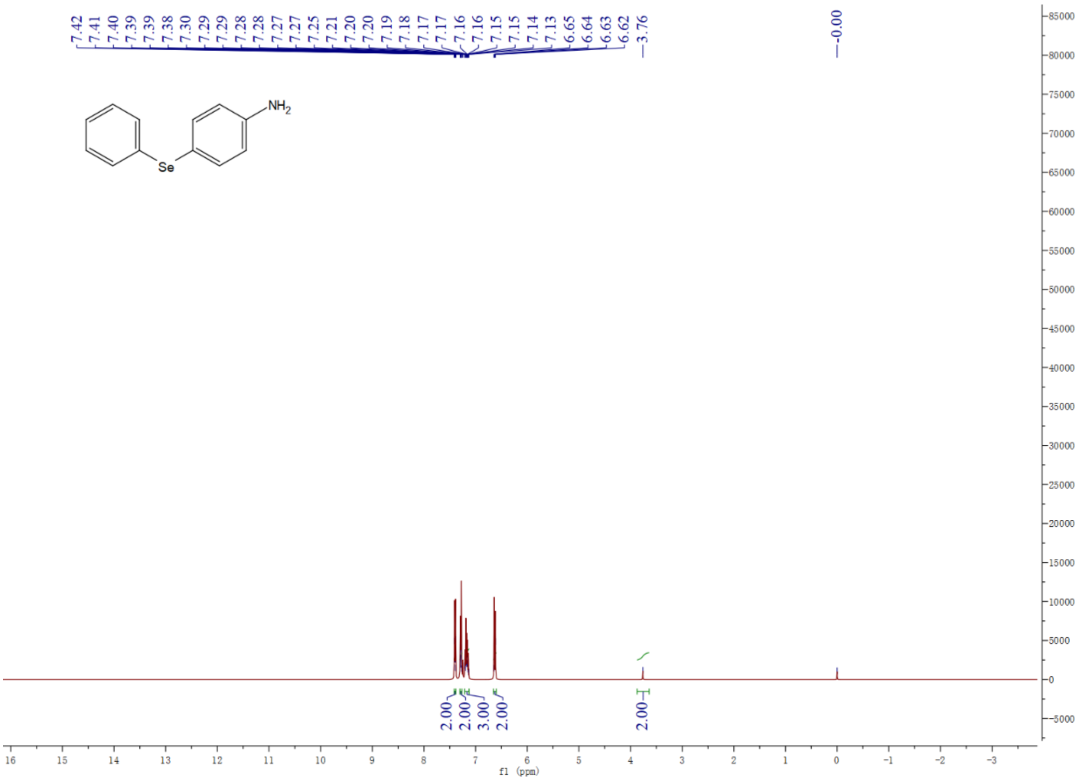

3af ^{13}C NMR

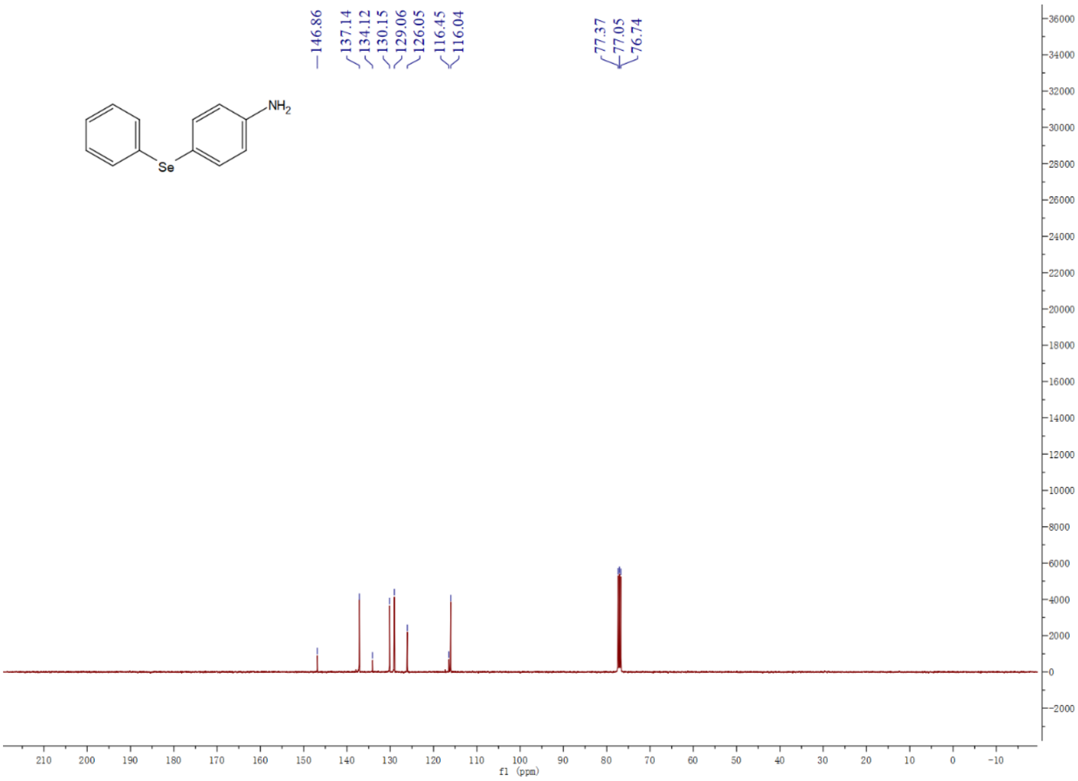

3ag ^{1}H NMR

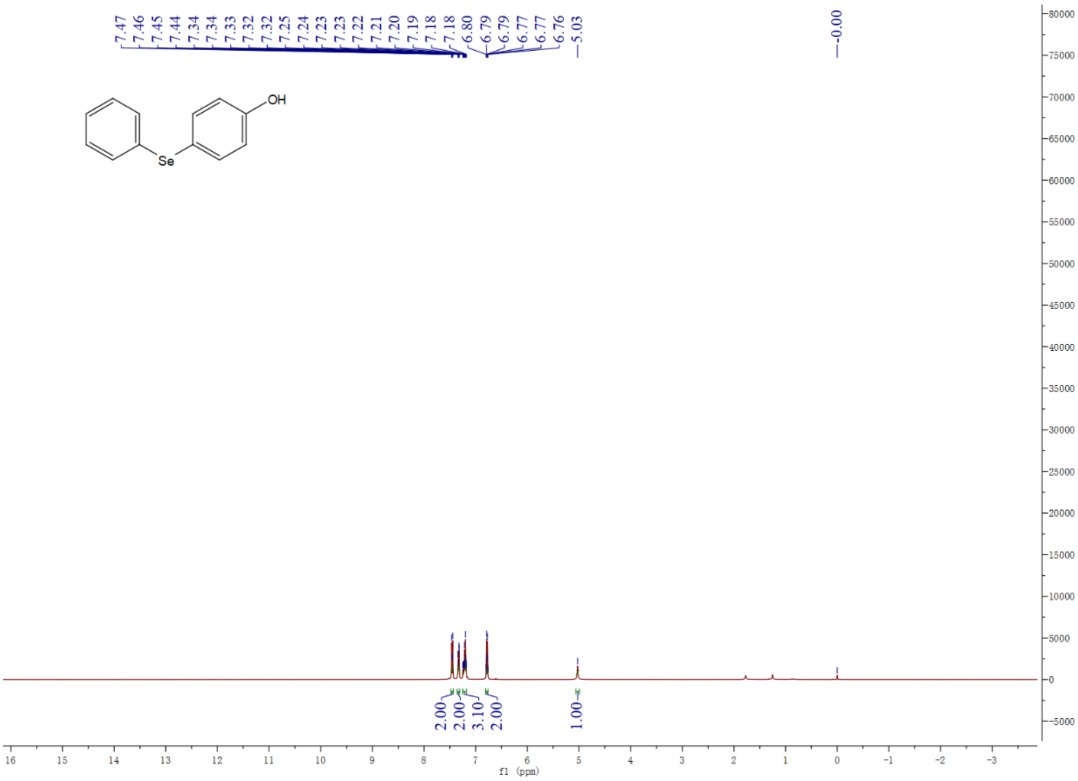

3ag ^{13}C NMR

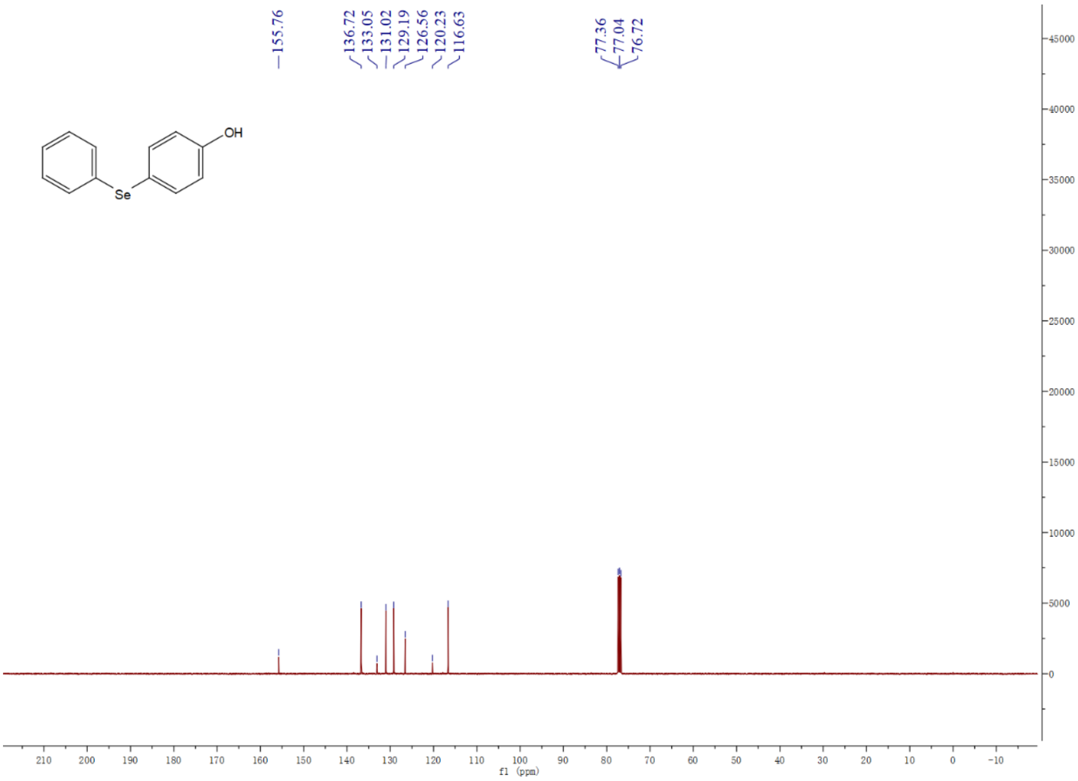

3ah ^{1}H NMR

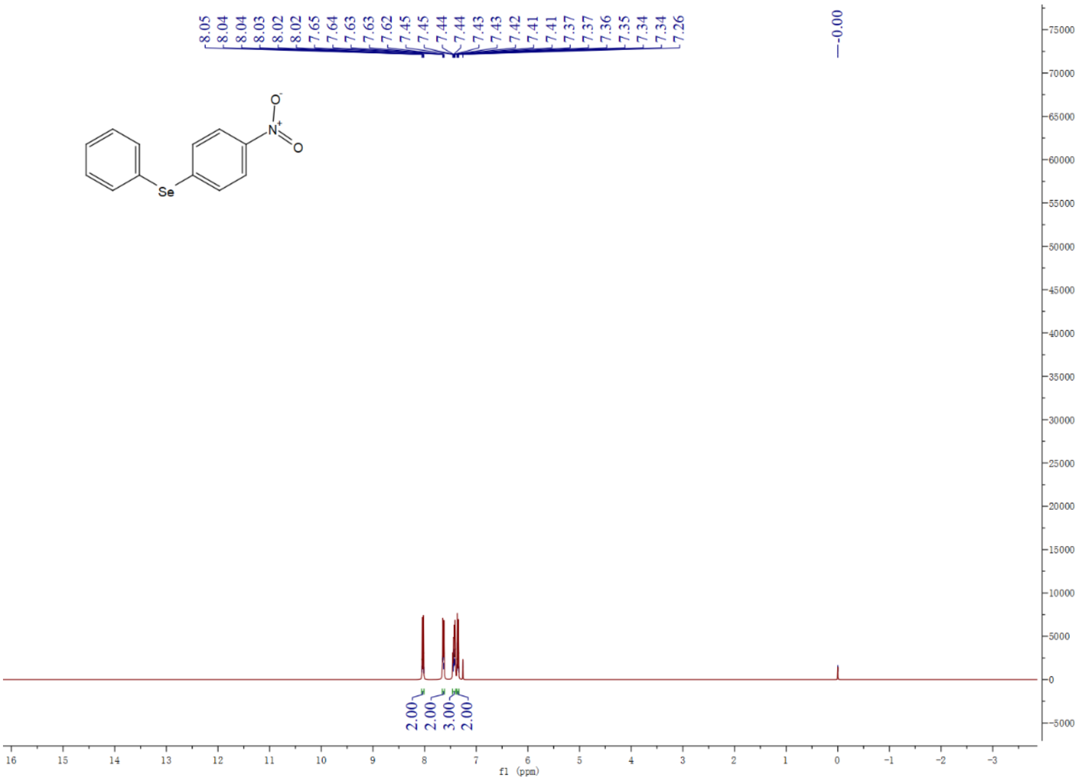

3ah ^{13}C NMR

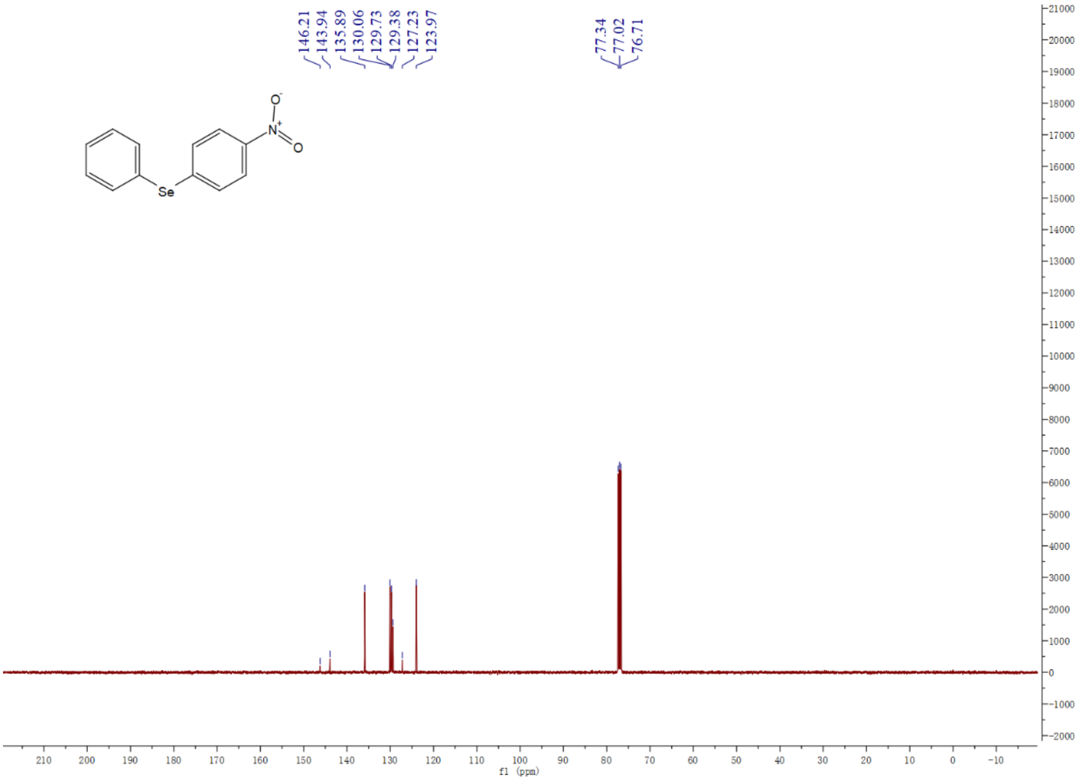

3ai ^{1}H NMR

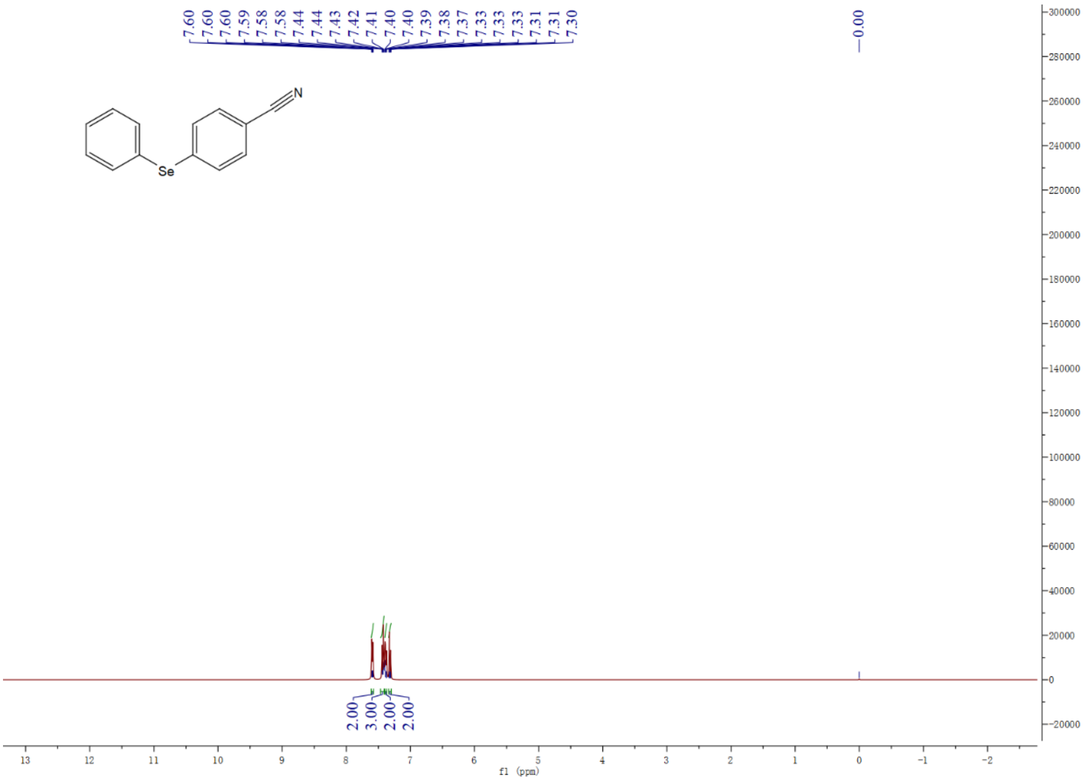

3ai ^{13}C NMR

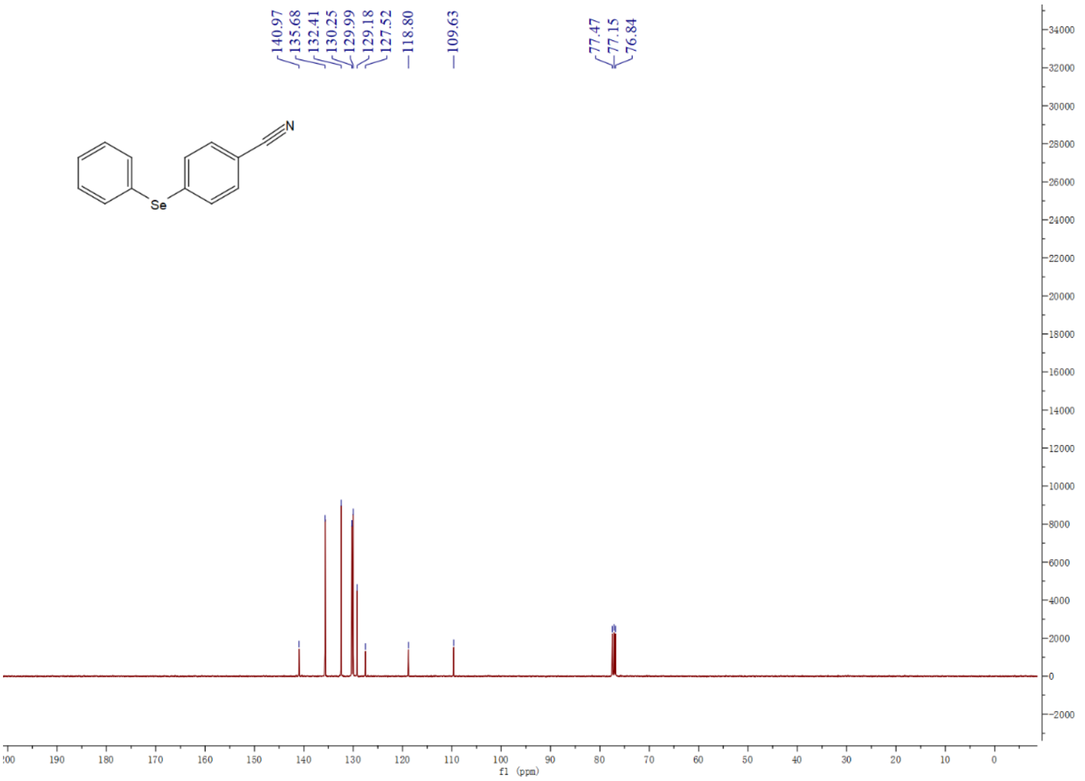

3aj ^{1}H NMR

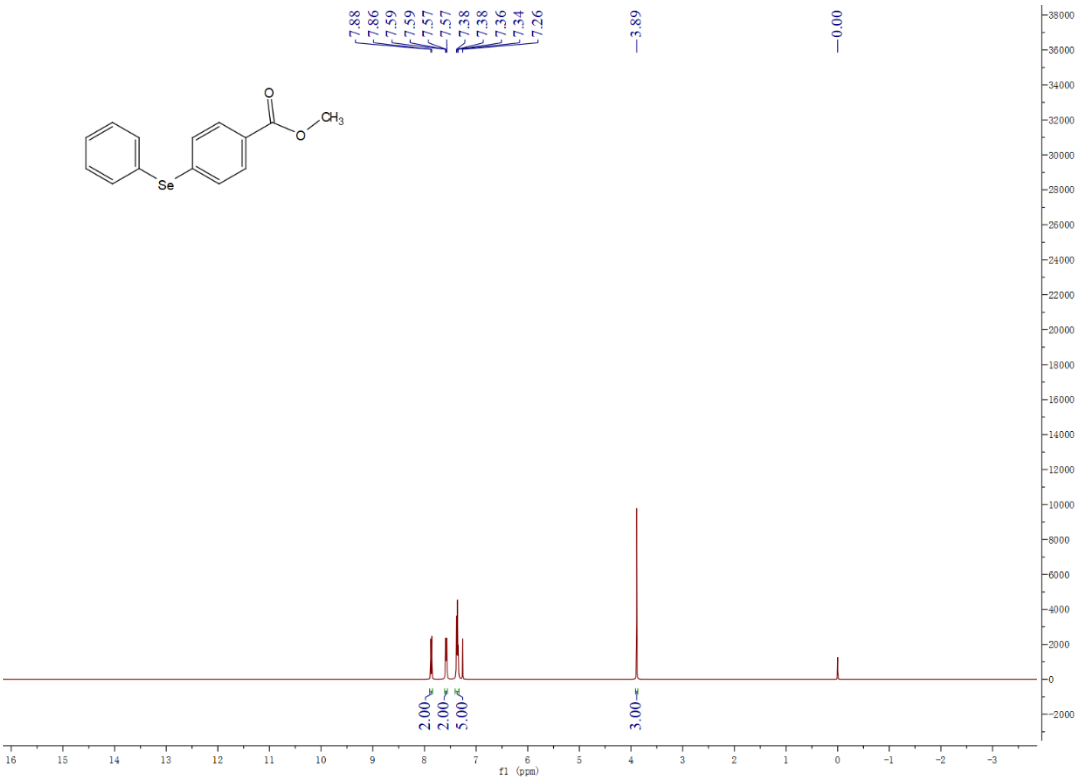

3aj ^{13}C NMR

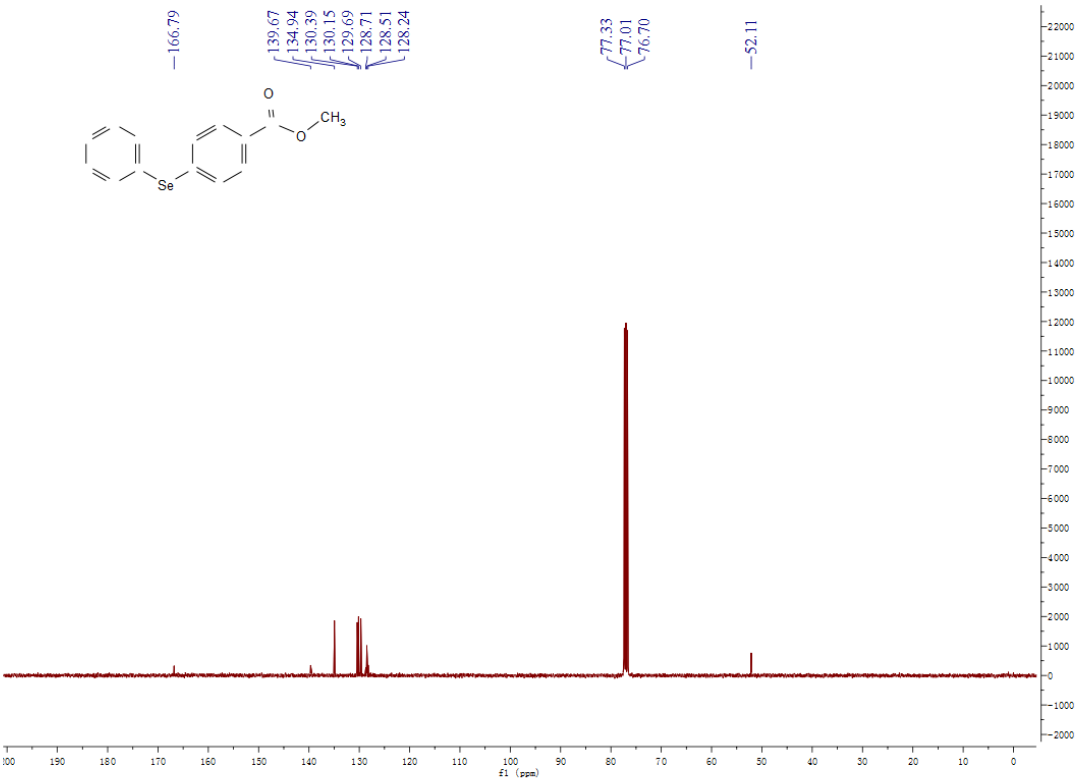

3ak ^{1}H NMR

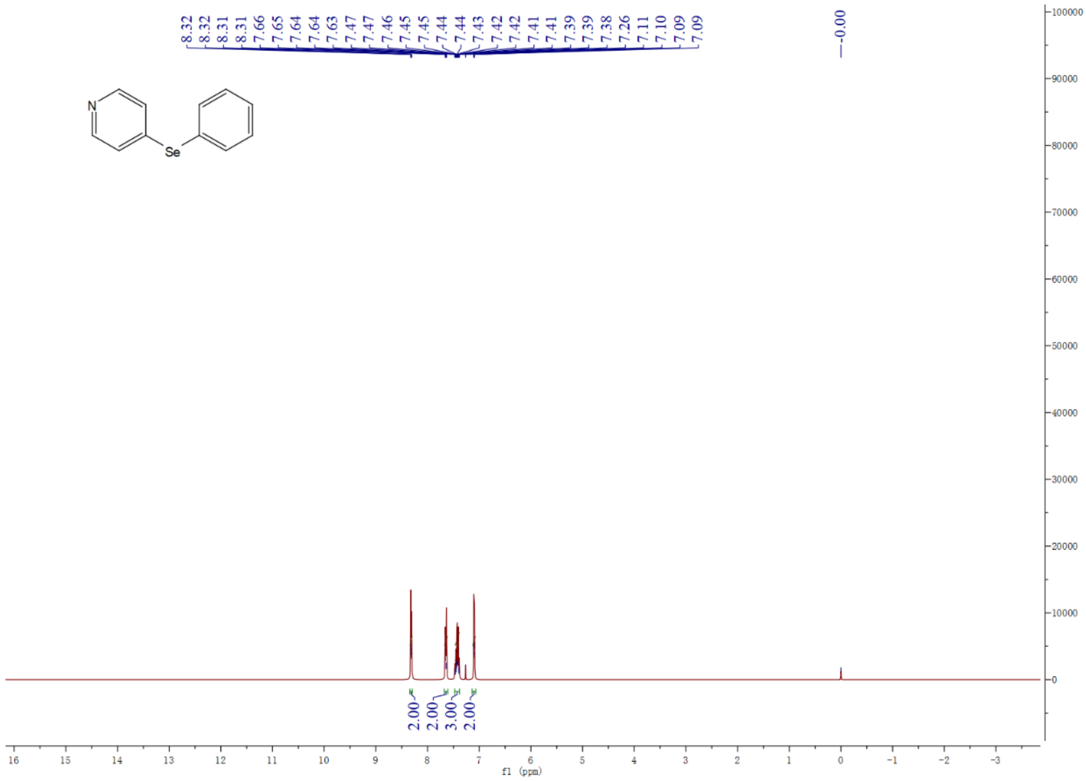

3ak ^{13}C NMR

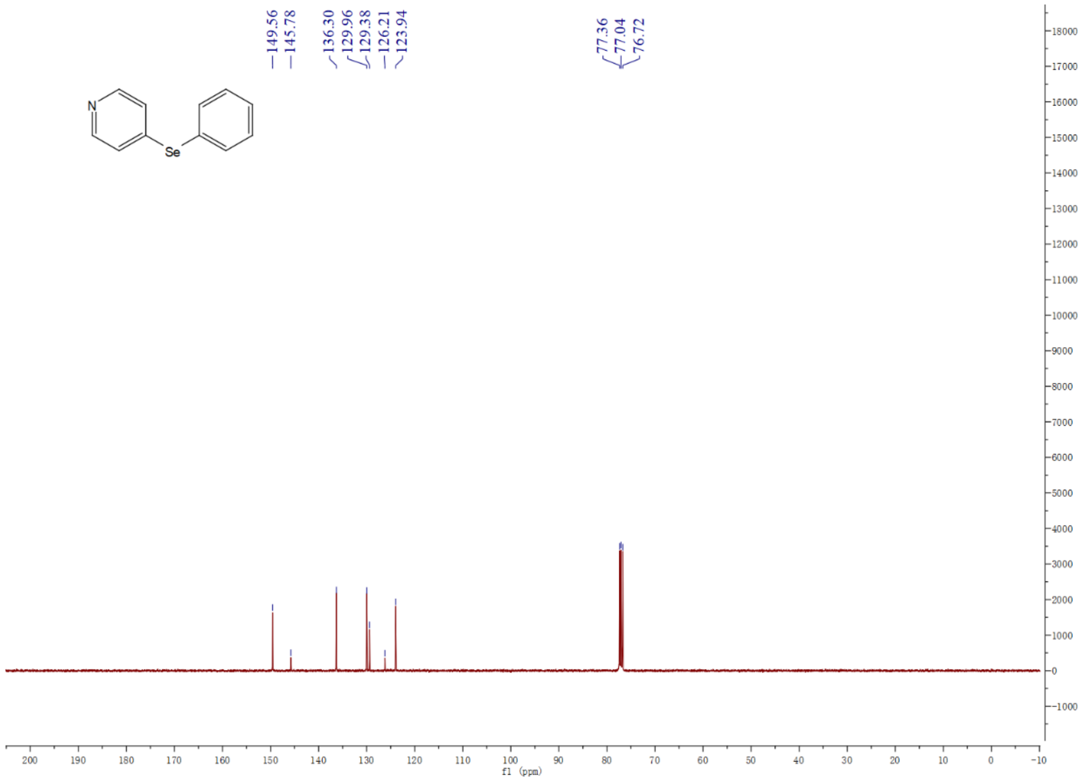

3al ^{1}H NMR

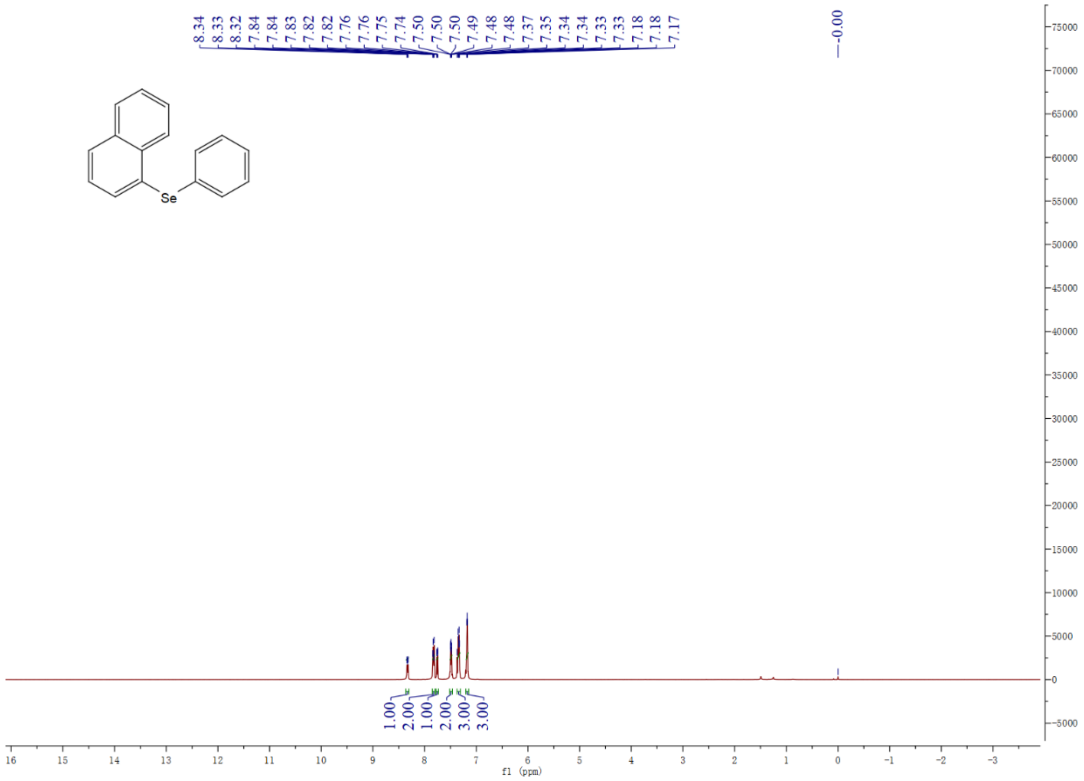

3al ^{13}C NMR

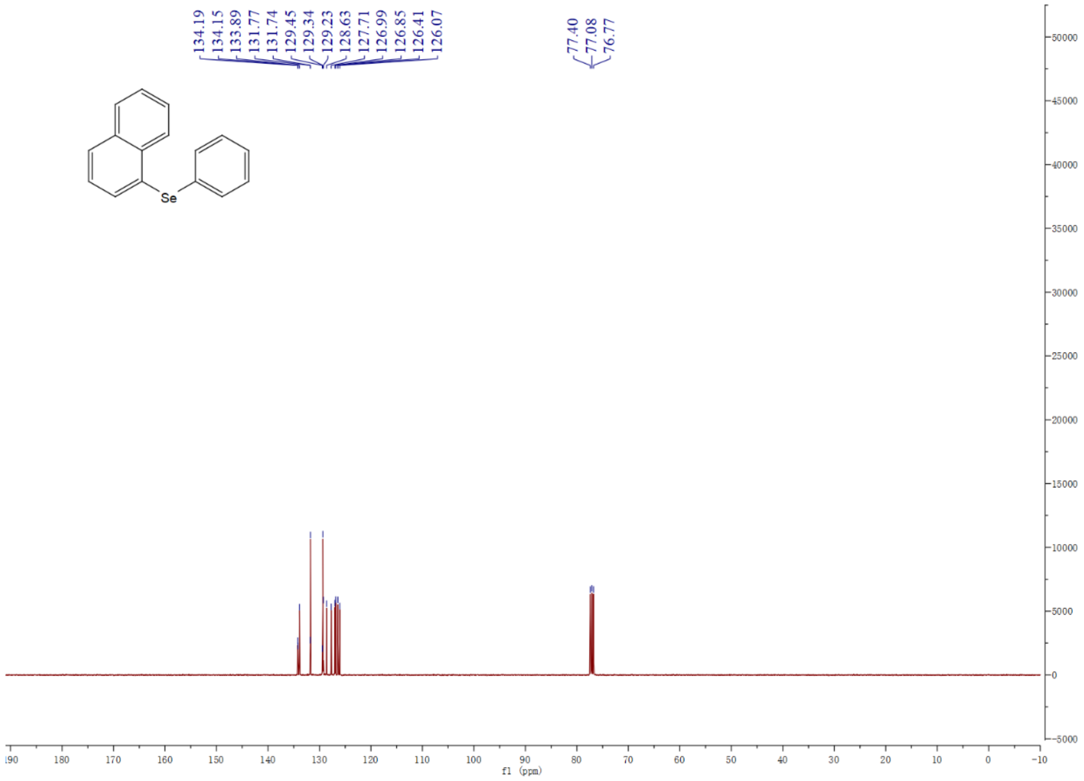

3am ^{1}H NMR

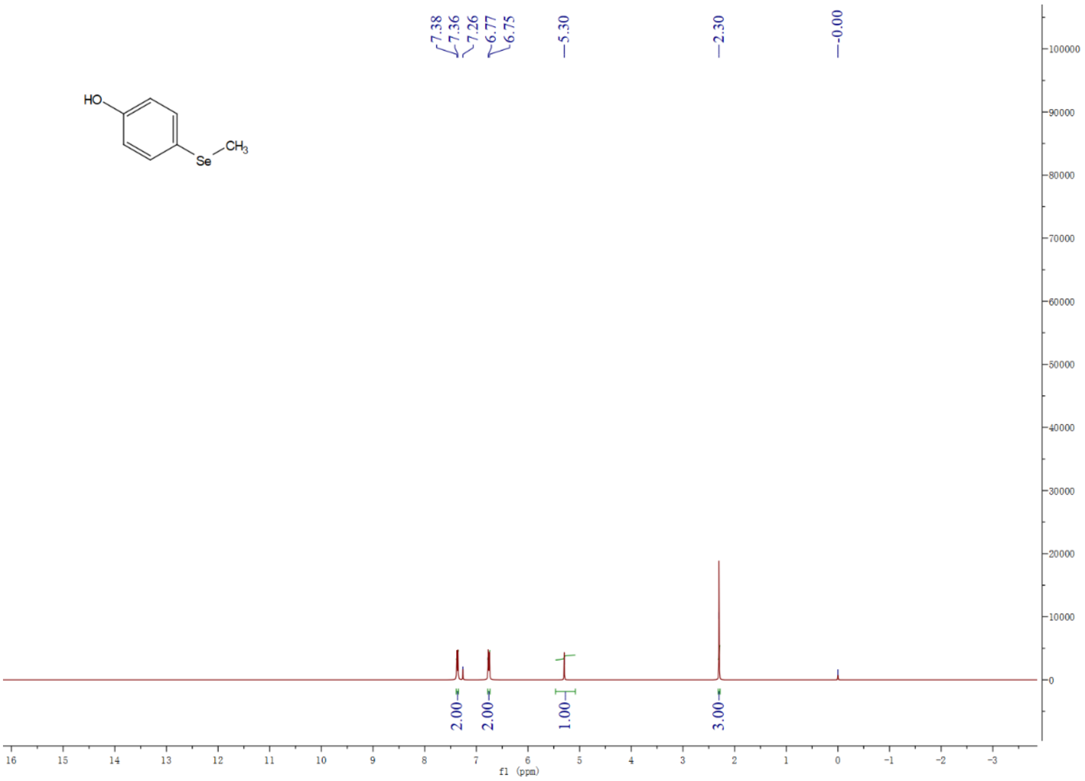

3am ^{13}C NMR

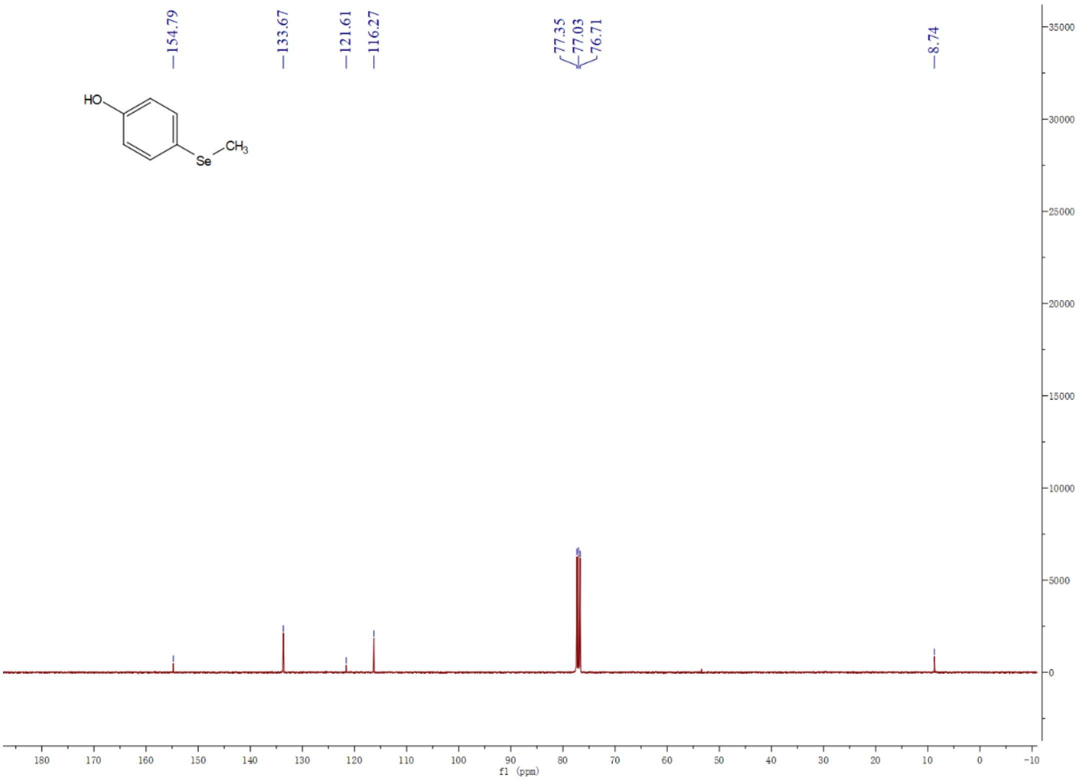

3an ^{1}H NMR

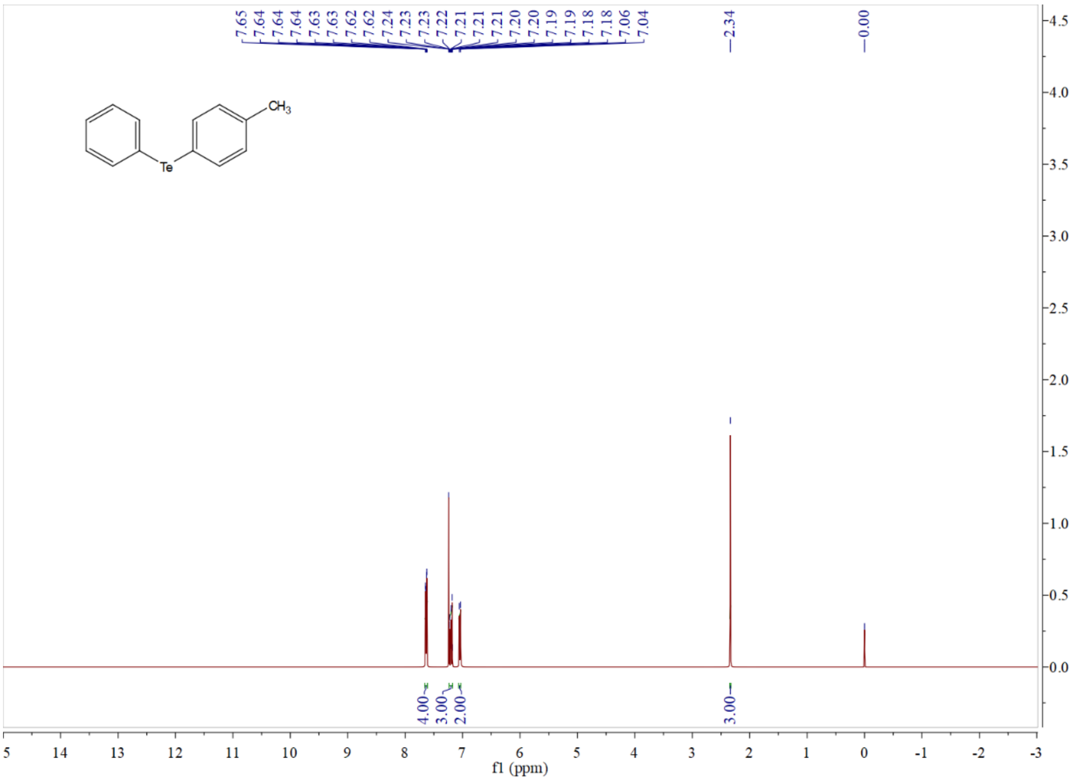

3an ^{13}C NMR

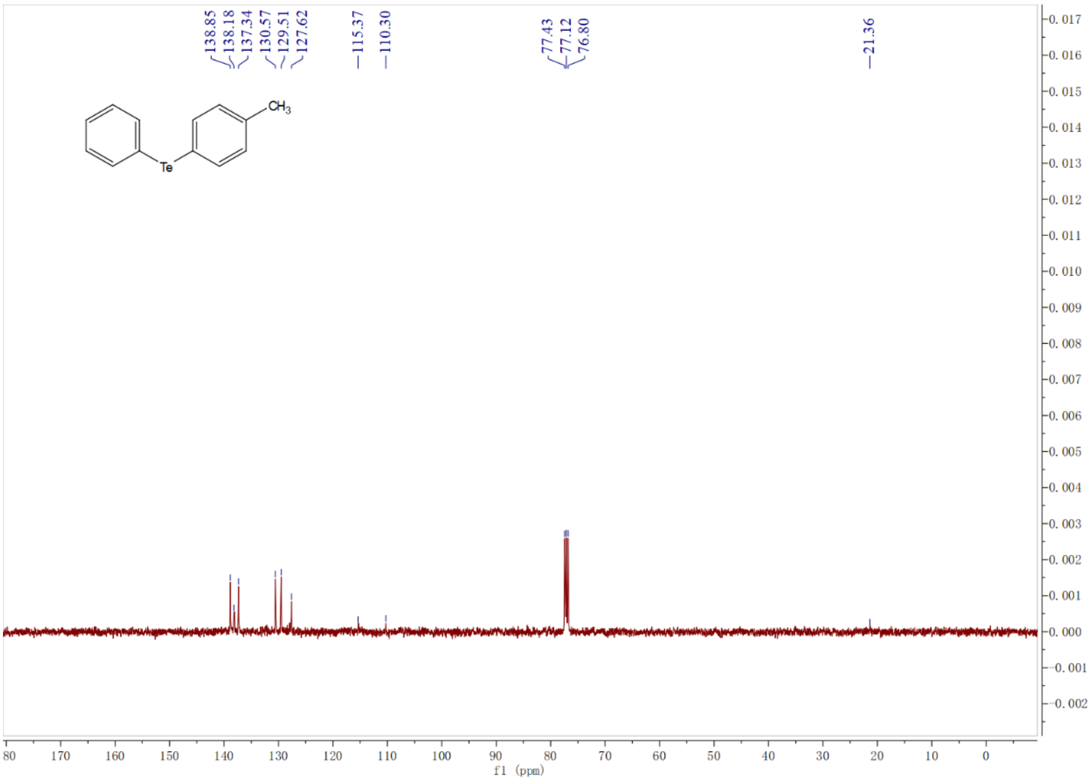

3ao ^{1}H NMR

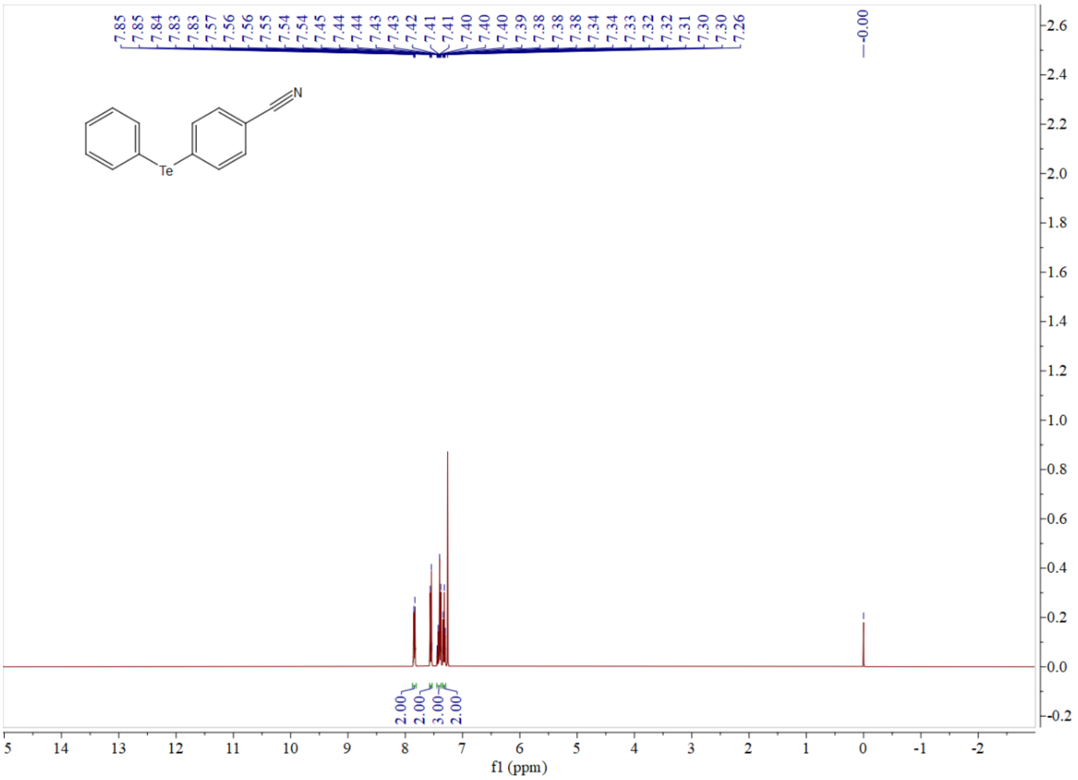

3ao ^{13}C NMR

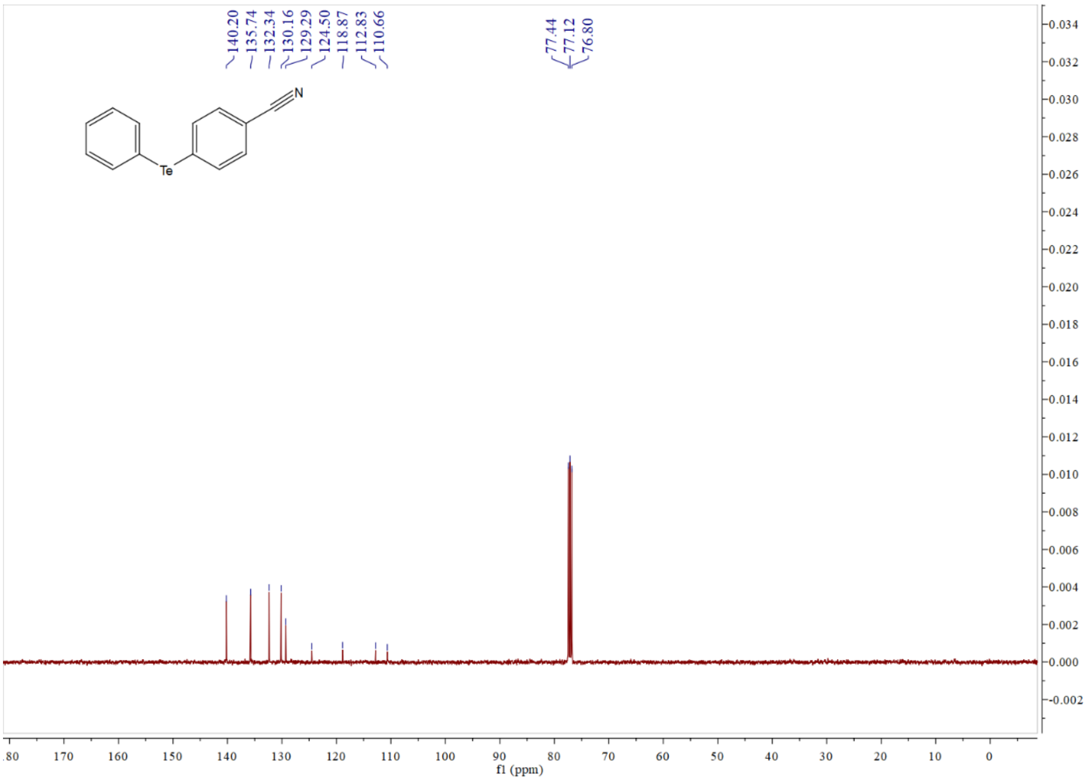

4a ^{1}H NMR

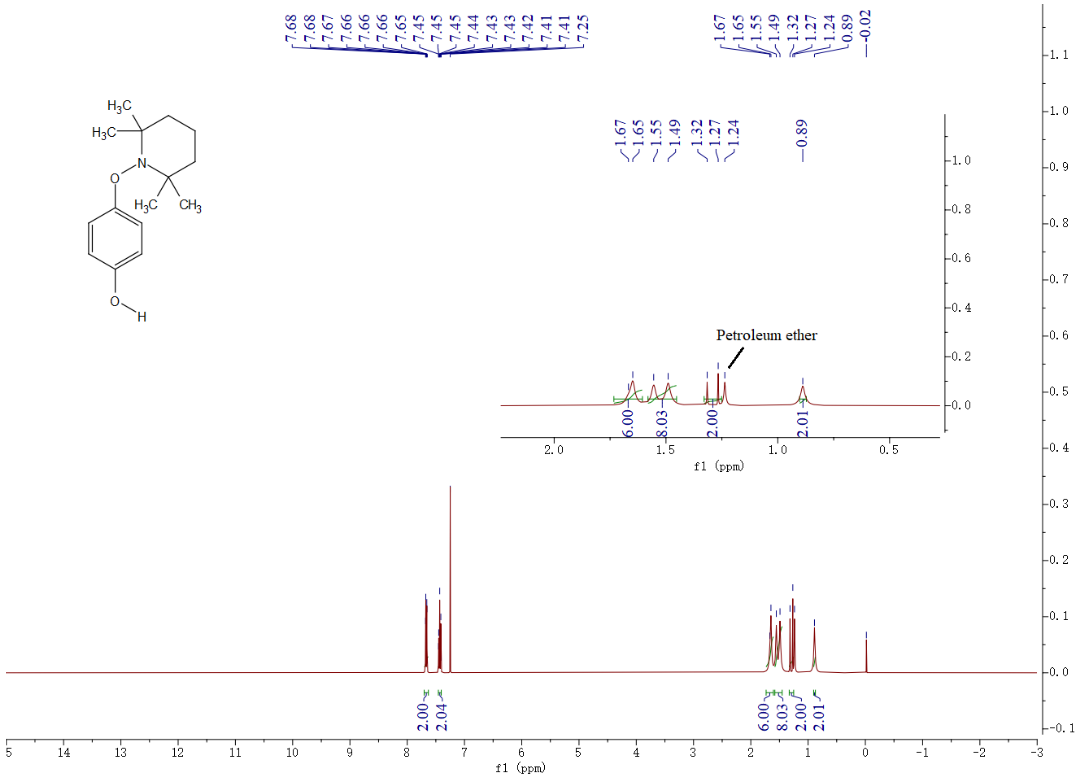

4a ^{13}C NMR

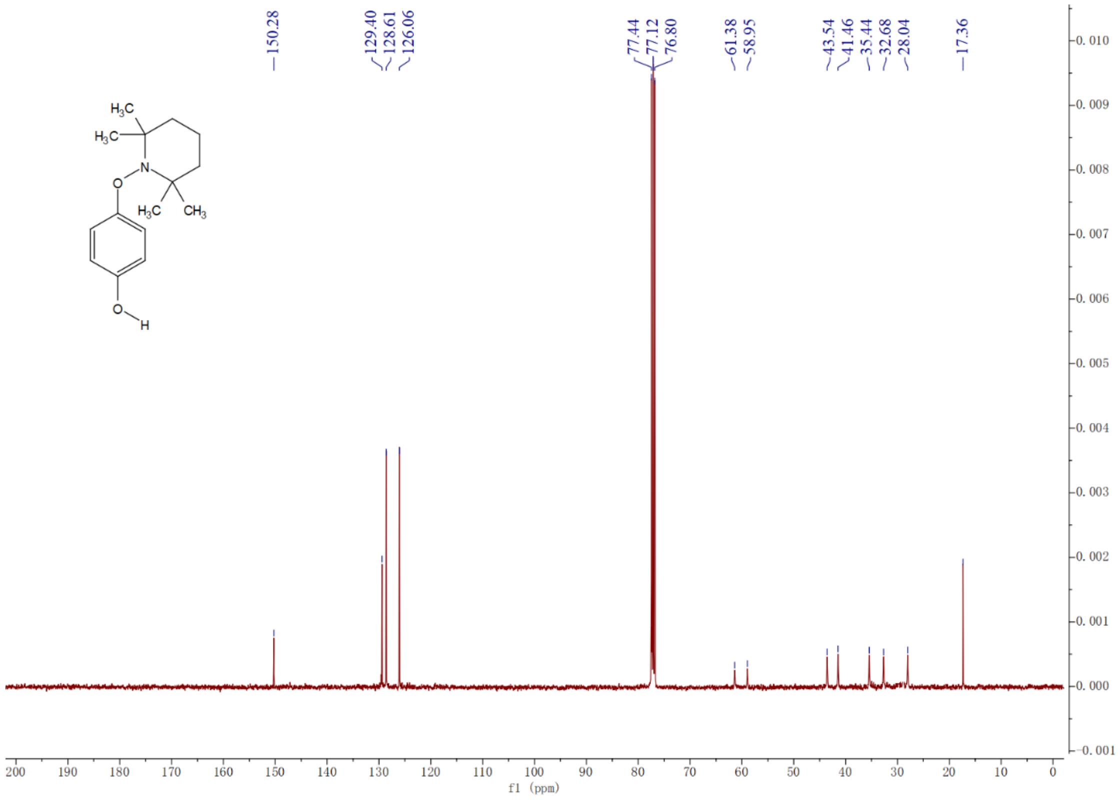

Supplement: Supplementary file 1 [file DataSheet1.PDF]
